# A Genetic History of the Balkans from Roman Frontier to Slavic Migrations

**DOI:** 10.1016/j.cell.2023.10.018

**Published:** 2023-12-07

**Authors:** Iñigo Olalde, Pablo Carrión, Ilija Mikić, Nadin Rohland, Swapan Mallick, Iosif Lazaridis, Matthew Mah, Miomir Korać, Snežana Golubović, Sofija Petković, Nataša Miladinović-Radmilović, Dragana Vulović, Timka Alihodžić, Abigail Ash, Miriam Baeta, Juraj Bartík, Željka Bedić, Maja Bilić, Clive Bonsall, Maja Bunčić, Domagoj Bužanić, Mario Carić, Lea Čataj, Mirna Cvetko, Ivan Drnić, Anita Dugonjić, Ana Đukić, Ksenija Đukić, Zdeněk Farkaš, Pavol Jelínek, Marija Jovanovic, Iva Kaić, Hrvoje Kalafatić, Marijana Krmpotić, Siniša Krznar, Tino Leleković, Marian M. de Pancorbo, Vinka Matijević, Branka Milošević Zakić, Anna J. Osterholtz, Julianne M. Paige, Dinko Tresić Pavičić, Zrinka Premužić, Petra Rajić Šikanjić, Anita Rapan Papeša, Lujana Paraman, Mirjana Sanader, Ivana Radovanović, Mirjana Roksandic, Alena Šefčáková, Sofia Stefanović, Maria Teschler-Nicola, Domagoj Tončinić, Brina Zagorc, Kim Callan, Francesca Candilio, Olivia Cheronet, Daniel Fernandes, Aisling Kearns, Ann Marie Lawson, Kirsten Mandl, Anna Wagner, Fatma Zalzala, Anna Zettl, Željko Tomanović, Dušan Keckarević, Mario Novak, Kyle Harper, Michael McCormick, Ron Pinhasi, Miodrag Grbić, Carles Lalueza-Fox, David Reich

**Affiliations:** 1 BIOMICs Research Group, Department of Zoology and Animal Cell Biology, University of the Basque Country UPV/EHU; Vitoria-Gasteiz, Spain.; 2 Ikerbasque—Basque Foundation of Science; Bilbao, Spain.; 3 Department of Human Evolutionary Biology, Harvard University; Cambridge, MA, USA.; 4 Institute of Evolutionary Biology, CSIC-Universitat Pompeu Fabra; Barcelona, Spain.; 5 Institute of Archaeology; Belgrade, Serbia.; 6 Department of Genetics, Harvard Medical School; Boston, MA, USA.; 7 Howard Hughes Medical Institute, Harvard Medical School; Boston, MA, USA.; 8 Broad Institute of MIT and Harvard; Cambridge, MA, USA.; 9 Archaeological Museum Zadar; Zadar, Croatia.; 10 Independent Researcher.; 11 Slovak National Museum–Archaeological Museum; Bratislava, Slovak Republic.; 12 Centre for Applied Bioanthropology, Institute for Anthropological Research; Zagreb, Croatia.; 13 Palisada Ltd.; Split, Croatia.; 14 School of History, Classics and Archaeology, University of Edinburgh; Edinburgh, UK.; 15 Archaeological Museum in Zagreb; Zagreb, Croatia.; 16 Faculty of Humanities and Social Sciences, University of Zagreb; Zagreb, Croatia.; 17 Division for Archaeological Heritage, Croatian Conservation Institute; Zagreb, Croatia.; 18 Center of Bone Biology, Faculty of Medicine, University of Belgrade; Belgrade, Serbia.; 19 Museum of Vojvodina; Novi Sad, Serbia.; 20 Institute of Archaeology; Zagreb, Croatia.; 21 Department for Archaeology, Croatian Conservation Institute; Zagreb, Croatia.; 22 Archaeology Division, Croatian Academy of Sciences and Arts; Zagreb, Croatia.; 23 Museum of Croatian Archaeological Monuments; Split, Croatia.; 24 Department of Anthropology and Middle Eastern Cultures, Mississippi State University; Starkville, MS, USA.; 25 Department of Anthropology, University of Nevada; Las Vegas, NV, USA.; 26 Kaducej Ltd.; Split, Croatia.; 27 Vinkovci Municipal Museum; Vinkovci, Croatia; 28 Trogir Town Museum; Trogir, Croatia; 29 Department of Anthropology, University of Kansas; Lawrence, KS, USA; 30 Department of Anthropology, University of Winnipeg; Manitoba, Canada; 31 Department of Anthropology, Slovak National Museum–Natural History Museum; Bratislava, Slovak Republic; 32 Laboratory for Bioarchaeology, Faculty of Philosophy, University of Belgrade; Belgrade, Serbia; 33 Department of Evolutionary Anthropology, University of Vienna; Vienna, Austria; 34 Department of Anthropology, Natural History Museum Vienna; Vienna, Austria; 35 Servizio di Bioarcheologia, Museo delle Civiltà; Rome, Italy; 36 Research Centre for Anthropology and Health (CIAS), Department of Life Sciences, University of Coimbra; Coimbra, Portugal; 37 Faculty of Biology, University of Belgrade; Belgrade, Serbia; 38 Serbian Academy of Sciences and Arts; Belgrade, Serbia; 39 Department of Classics and Letters, University of Oklahoma; Norman, OK, USA; 40 Santa Fe Institute; Santa Fe, NM, USA; 41 Department of History, Harvard University; Cambridge, MA, USA; 42 Max Planck-Harvard Research Center for the Archaeoscience of the Ancient Mediterranean, Harvard University; 43 Human Evolution and Archaeological Sciences, University of Vienna; Vienna, Austria; 44 Department of Biology, University of Western Ontario; London, ON, Canada; 45 Department of Agriculture and Food, Universidad de La Rioja; Logroño, Spain; 46 Museu de Ciències Naturals de Barcelona; Barcelona, Spain

**Keywords:** Balkan peninsula, Cosmopolitanism, the Roman Empire, Great Migration Period, demographic changes, population dynamics

## Abstract

The rise and fall of the Roman Empire was a socio-political process with enormous ramifications for human history. The Middle Danube was a crucial frontier and a crossroads for population and cultural movement. Here we present genome-wide data from 136 Balkan individuals dated to the 1^st^ millennium CE. Despite extensive militarization and cultural influence, we find little ancestry contribution from peoples of Italic descent. However, we trace a large-scale influx of people of Anatolian ancestry during the Imperial period. Between ~250-550 CE, we detect migrants with ancestry from Central/Northern Europe and the steppe, confirming that “barbarian” migrations were propelled by ethnically diverse confederations. Following the end of Roman control, we detect the large-scale arrival of individuals who were genetically similar to modern Eastern European Slavic-speaking populations, who contributed 30-60% of the ancestry of Balkan people, representing one of the largest permanent demographic changes anywhere in Europe during the Migration Period.

## Introduction

At its height in the 2^nd^ century CE, the Roman Empire stretched from Mesopotamia and Arabia in the east to Britain in the west, from the Rhine and Danube rivers in the north, to the Sahara Desert in the south^1^. The massive extraction and mobilization of resources from western Britain to the eastern desert of Egypt by the imperial polity stimulated the movement of humans, via both coercive and consensual processes, effectively restructuring populations across this vast zone.

The Balkan Peninsula has been a historic crossroads of eastern and western Mediterranean cultures, as well as continental European influences from the north and Mediterranean from the south. From the 1^st^ to the 6^th^ centuries CE, the Roman Empire’s Middle Danube frontier in present-day Croatia and Serbia was a zone of defense, confrontation, and exchange with populations living north of the frontier. This region was also a source of significant mineral wealth and a crucial hinge in a ~2000 km long corridor of military and communications infrastructure linking the Black Sea to the Black Forest^2^. Following the establishment of Roman control in the early 1^st^ century CE, the region became increasingly urbanised and culturally “Romanised”. Between ca. 268 and 610 CE, more than half of all Roman emperors belonged to families originating in the Middle Danube^3^. In late antiquity, the region experienced numerous invading groups labelled by historical sources as Goths, Huns, Gepids, Avars, Heruls, Lombards, or Slavs^4^; non-Romans also were increasingly recruited into the Roman army from peoples across the northern frontier. Various Germanic groups settled in the Danubian region, and some late antique cultural artifacts (and associated human remains) have been attributed to “Germanic”-related influence^4^. Nevertheless, the Roman Empire retained some control over this frontier zone into the second half of the 6^th^ century. But over the later 6^th^ and 7^th^ centuries, as the Roman Empire (ruled from Constantinople, ~1000 km away) was confronted by pandemic plague and environmental, political, and military challenges, Roman control over this frontier was lost^5,6^. The end of imperial hegemony in the Balkans coincided with further population movements patchily attested in the historical record, including the arrival of the Slavs, whose migration to the region was, much like the arrival of Germanic groups in post-Roman Britain, significant enough to have a particularly lasting impact, reflected in the south Slavic languages widely spoken in the Balkans today^7^. Slavic-associated ancestry^8^ in present-day populations has been identified as far as the Peloponnese (the southern tip of the Balkan Peninsula in present-day Greece), but the degree, timing and character of permanent demographic impacts across the region have been poorly understood.

While historians have explored Roman imperialism through the lenses of geopolitics, institutions, cultures, and economics, the scale of the Roman Empire’s impact on the population history of its constituent territories is only now becoming understood through the recovery and analysis of ancient DNA. Ancient DNA can complement or challenge conventional archaeological and textual evidence, offering direct insights into individual histories and processes of population change, including social groups whose movements have hitherto been mostly invisible in elite-dominated sources. In fact, archaeogenetic studies are starting to confirm the hints preserved in the documentary record of the empire’s remarkable capacity to foster mobility and mixture^9-11^. For instance, a man from Roman York in northern England (ancient *Eboracum*) showed affinities to modern Middle East populations^12^ and individuals with a high proportion of North African ancestry were found in southern Iberia^13^. A study of 48 skeletons from Rome’s hinterland in the Imperial period showed that at the height of the Empire, genetic ancestry became much more heterogeneous than in previous periods and shifted towards Near Eastern populations^14,15^ and a similarly dramatic shift was shown to extend deep into central Italy^16^. Archaeological DNA is also being used to trace the timing, nature, and extent of migrations and population change in post-Roman Europe, from Anglo-Saxon England^17^ to Lombard Italy^18^. The Middle Danube frontier, a crucial axis for the Roman Empire, has not been systematically characterized using archaeogenetic data.

To explore the population history of the Balkans (bounded by the Adriatic, the Central Mediterranean, the Aegean Seas and, to the north, by the Middle and Lower Danube and Sava rivers) in the high Imperial (ca. 1-250 CE), late Imperial (ca. 250-550 CE), and post-Roman (ca. 550-1000 CE) periods, we present new genomic data from 136 ancient individuals from present-day Croatia and Serbia, and 6 from Austria, the Czech Republic and Slovakia, along with information on the archaeological context of their burial (Data S1, section 1). This dataset furnishes insights into the population dynamics of a vital frontier zone, including changes likely associated with the introduction of Slavic languages and the making of modern Balkan populations.

## Results

### Data generation

**Figure 1.**
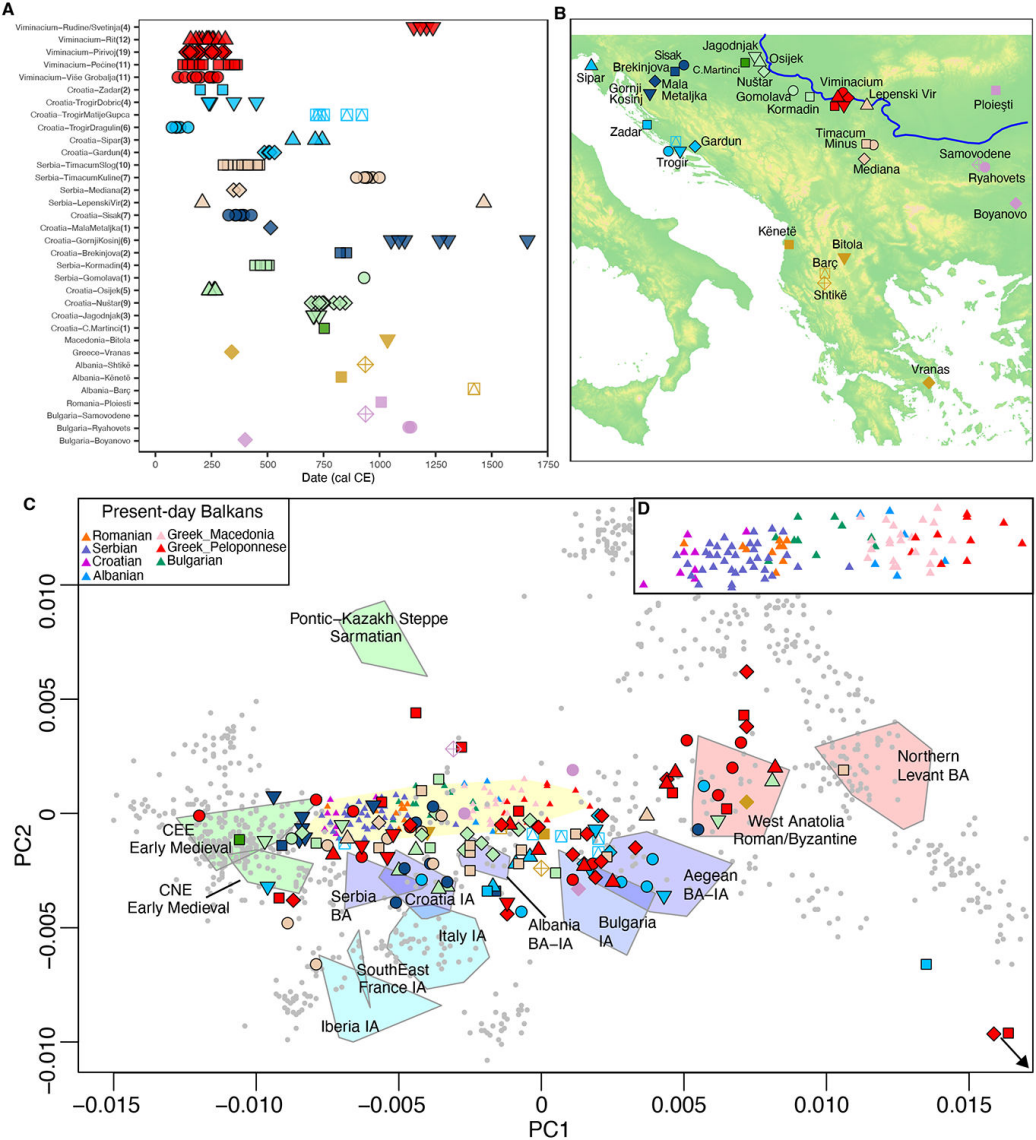
Overview of ancient Balkan individuals analyzed in this study. (A) Chronological distribution. Individuals with newly reported data are represented by symbols with a black outline. (B) Geographical location of archaeological sites. (C) PCA of the West-Eurasian genetic variability showing present-day individuals as grey circles (except present-day Balkan populations that are displayed with open colored triangles), and relevant ancient populations as colored polygons (Balkan Iron Age groups in blue, Southern European Iron Age groups in light blue, ancient Near Easter groups in red and ancient Steppe, Central, Northern and Eastern European groups in green) including all individuals in each population (Data S2, Table 3). Ancient individuals were projected onto the PCs computed on present-day West Eurasians; their shape and color are the same as in panels (A) and (B). This PCA is a zoom-in version of Figure S1. (D) Closer view of the present-day Balkans genetic cline from panel (C). CNE: Central/Northern European; CEE: Central/Eastern European; BA: Bronze Age; IA: Iron Age.

We extracted DNA from 146 ancient Balkan samples (Data S2, Table 1), of which 136 yielded genome-wide data after in-solution hybridization enrichment with either the ‘1240k’^19,20^ panel of about 1.23 million single nucleotide polymorphisms (SNPs) or the “Twist” panel which targets an enlarged set of 1.43 million SNPs (the same set of core SNPs and supplementary content)^21^ (STAR Methods). The individuals were excavated from 20 different sites (Figure 1A-B) representing a variety of regions and archaeological contexts including, among others, *Viminacium* (Kostolac, Serbia), the capital of the Roman Upper Moesia province located at the confluence of the Mlava River and the Danube, where we report data from 57 individuals from 6 different necropolises^22^ (Data S1, section 1), Roman colonies such as *Iader* (Zadar, Croatia) on the Adriatic coast and *Siscia* (Sisak, Croatia) and *Mursa* (Osijek, Croatia) on the Pannonian road from the Adriatic to the Danube, military fortresses such as *Tilurium* (Gardun, Croatia) and *Timacum Minus* (Ravna, Serbia)^23^, and early medieval necropolises such as Jagodnjak (Croatia) and Nuštar-Dvorac (Croatia). To place the results in a geographic and temporal context, we also generated genome-wide data from six early medieval central European individuals from Austria, the Czech Republic and Slovakia, Affymetrix Human Origins SNP array^24^ data from modern Serbs (*n*=37) (Data S2, Table 2), and 38 new radiocarbon dates (Data S2, Table 9).

For genome-wide analyses, we filter out 13 newly reported individuals with fewer than 20,000 SNPs and/or with evidence of contamination (STAR Methods), and include 15 individuals with previously reported genomic data^25^ from present-day Croatia, Albania, North Macedonia, Greece, Romania and Bulgaria, for a total of 138 Balkan individuals mostly dated to ~1-1000 CE (Data S2, Table 1; Figure 1A-B).

### High ancestry heterogeneity

To study the 138 Balkan individuals, we performed Principal Component Analysis (PCA) by projecting them and other ancient individuals from relevant periods and regions onto the axes computed on 1036 present-day West-Eurasian (WE) individuals (Figure 1C; Figure S1) genotyped on the Affymetrix Human Origins array.

A key feature of the data is the presence of two parallel genetic clines running along PC1 (Figure 1C). The first, which we call the “Bronze-Iron Age Balkan cline”, includes southern (Aegean) Bronze and Iron Age groups on the right extreme closer to Near Easterners (larger values in PC1), and northern Bronze and Iron Age groups from modern Croatia and Serbia on the left extreme closer to Central/Northern/Eastern European populations (lower values in PC1); Bronze-Iron Age groups from Bulgaria and Albania take intermediate positions. This Bronze-Iron Age cline is paralleled by the “present-day Balkan cline”, which is shifted upwards (higher values in PC2) with respect to the Iron Age cline but displays in PC1 the same geographical pattern of southern Balkan populations such as the Greeks on the right, and northern Balkan populations such as Croatians on the left (Figure 1D). The maintenance of the same geographical pattern along PC1 in both clines points to some degree of local continuity from the Iron Age across the entire region, along with the strong impact of migration from outside the Balkans, affecting all groups from North to South over the past 2,000 years. Irrespective of modern nation-state boundaries, populations in our study area have been shaped by similar processes of migration and change.

Balkan individuals in our 1^st^ millennium CE transect showed higher ancestry heterogeneity in PCA compared to previous Iron Age Balkan populations (variances in PC1 and PC2 values are significantly different with p = 0.045 and 0.0046, respectively), with most spreading along either the present-day or the Bronze-Iron Age Balkan clines. This suggests that key demographic events involved in the formation of present-day groups had already taken place by ~1000 CE. The remaining individuals plot far beyond the two Balkan genetic clines and likely represent cases of sporadic long-distance mobility that provide evidence concerning the regions acting as demographic sources for the Balkans during this period.

Given the high ancestry heterogeneity observed in our dataset, even within the same sites and necropolises, we estimate ancestry proportions separately for each individual. We used *qpAdm* ^26,27^ with Balkan Iron Age populations as the local ancestry source, and earlier and contemporaneous populations from neighboring regions as proximate sources for newly arriving ancestries (Data S1, section 4).

### Large-scale demographic input from Western Anatolia

**Figure 2.**
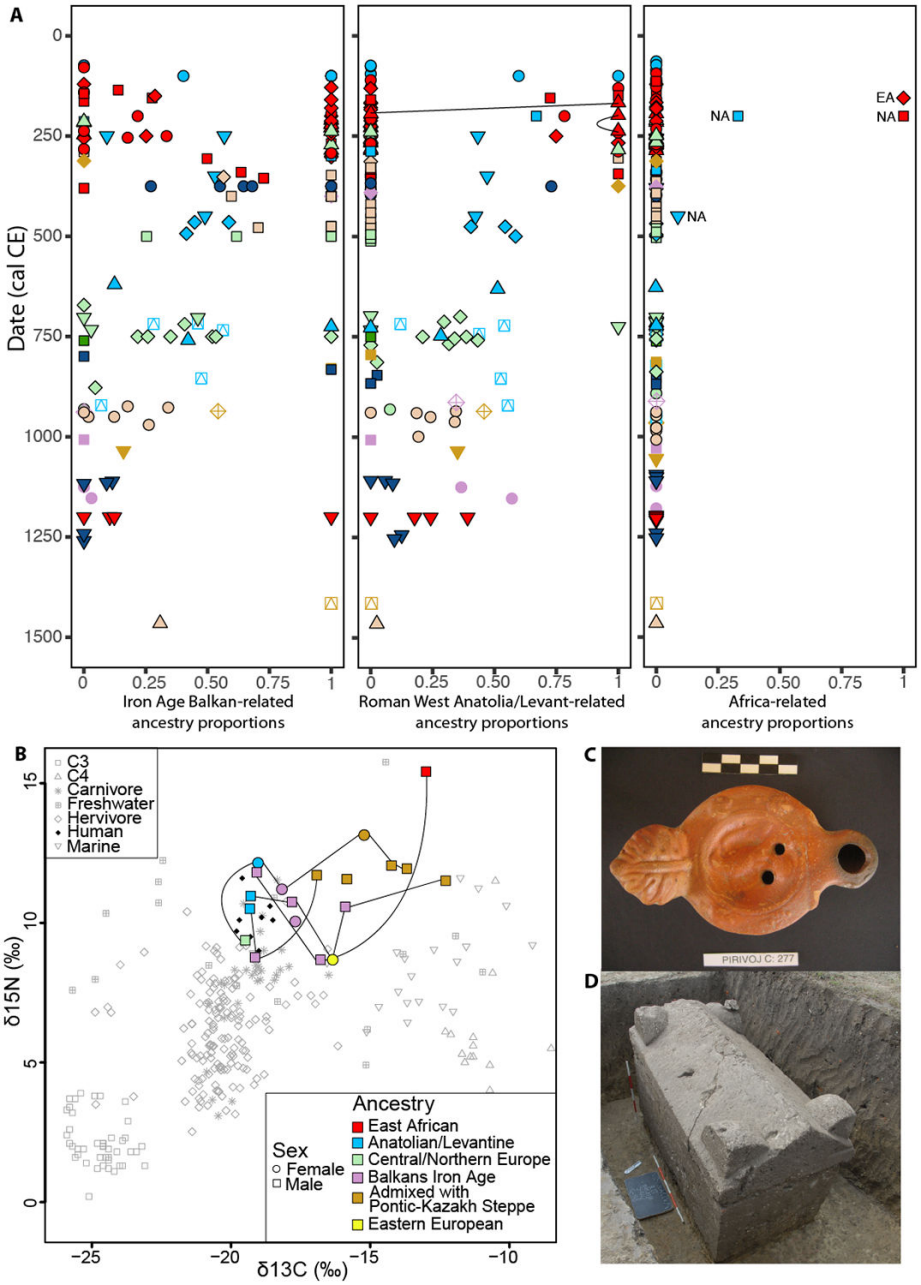
A diversity of ancestral origins. (A) By-individual estimates of Iron Age Balkan, West Anatolian/Levantine, and African-related ancestry proportions between 0-1500 CE, computed with *qpAdm*. Two pairs of individuals buried in the same sarcophagi at Rit Necropolis (*Viminacium*) are connected through black lines. (B) δ15N and δ13C stable isotope values (Data S2, Table 9) of ancient Balkan individuals between 0-500 CE obtained from tooth roots, plotted alongside published environmental data and humans from related geographic and chronological contexts^34,44-47^. Individuals buried at the same necropolis are connected through lines. (C) Oil lamp depicting an eagle found on individual G-103’s (I15499) grave at Pirivoj, *Viminacium*. (D) Sarcophagus of grave 148 at Rit Necropolis, *Viminacium*.

Around half of the 45 individuals between ~1-250 CE can be fitted with *qpAdm* models featuring only Balkan Iron Age groups (Figure 2A) and are characterized by a high frequency (5 out of 10) of Y-chromosome lineage E-V13 (Data S1, section 2), which has been hypothesized to have experienced a Bronze-to-Iron Age expansion in the Balkans^28^. These individuals, sampled from Roman towns such as *Viminacium*, *Tragurium* (Trogir) and *Mursa* (Osijek), are consistent with being direct descendants of local Balkan Iron Age populations (Figure 2A), pointing to a high degree of integration of the local population into Roman society. Despite the exceptional number of Roman colonies in the region, and the large military presence along this frontier, there is little ancestry contribution from populations long established in the Italian Peninsula, a pattern exemplified by the almost complete absence in our Balkan transect of Y-chromosome lineage R1b-U152, the most common paternal lineage in Bronze Age and Iron Age populations in the Italian Peninsula^15,16,29^. The prevalence of cremation burials in the earliest centuries could bias the sample, but even after the transition to inhumation burial around the 2^nd^ century, ancestry contributions from populations of Italian descent are not detectable. Rome’s cultural impact on the Middle Danube was deep, but our findings suggest that it was not accompanied by large-scale population movement from the metropole, at least by the descendants of central Italian Iron Age populations.

The Roman Empire did, however, stimulate demographic change in the Balkans. In this early period, ~1/3 of the individuals (15 out of 45) fall beyond the Balkan clines in PCA (Figure 1C; Figure S4) but close to Near Easterners, and can be modeled as deriving their ancestry predominantly from Roman/Byzantine populations from Western Anatolia and, in one case, from Northern Levantine groups (Figure 2A; Data S2, Table 6). Most of these individuals were excavated at four different *Viminacium* necropolises, but we also found them at other urban centers such as *Tragurium* (Trogir) and *Iader* (Zadar). A very strong demographic shift towards Anatolia is also evident in Rome and central Italy during the same period^15,25^ and demonstrates long-distance mobility plausibly originating from the major eastern urban centers of the Empire such as Ephesus, Corinth, or Byzantium/Constantinople; our results show that these migrants had a major demographic impact not only on the Imperial capital but also on other large towns on the Empire’s northern periphery. Our data also provide insights concerning the social dynamics of this demographic process. Unlike the Balkan Iron Age ancestry group whose sex ratio was evenly balanced (11 females out of 22 individuals), the 12 adult individuals with full Anatolian/Levantine ancestry included only 2 women (p = 0.019 for a one-sided binomial test for more males than females). This points to a larger contribution of Near Eastern men, but could also result from different burial customs between the sexes. People with Anatolian ancestry and people with local Balkan ancestry were not spatially segregated in burial nor, for the most part, culturally distinct in burial customs or grave goods. They admixed and were buried at the same necropolises, even side-by-side as in tomb G-148 at Rit necropolis (Figure 2D). However, the evidence may also point to some degree of social stratification, since all 3 individuals at the Rit necropolis of Anatolian origin were buried in stone sarcophagi with exceptionally rich grave goods (Figure 2D and Data S1, section 1)^30^. The main source of migrants to the region shifted away from Anatolia after ~300 CE (Figure 2A), but together with the ancestral legacy of local Balkan Iron Age groups, Anatolian-related ancestry persisted in admixed form into the later Medieval individuals (Figure 2A) with a mean of 23% (95% CI = 17-29%), indicating that this was a deep and lasting demographic impact.

### Migrants from sub-Saharan Africa and North Africa

Our newly reported data also revealed sporadic long-distance mobility. Three men who likely lived in the 2^nd^ or 3^rd^ centuries CE fell outside European and Near Eastern variability (Figure S1), close to present-day and ancient Africans (Figure S2A). Proximal *qpAdm* modeling confirmed these observations (Figure 2A; Data S2, Table 6) with 33% and 100% North African ancestry for individuals I26775 (*Iader*) and I32304 (*Viminacium* Pećine), respectively, while I15499 (*Viminacium* Pirivoj) could be modeled using only ancient East African populations, supporting an East African ancestral origin and agreeing with his uniparental markers mtDNA L2a1j and Y-chromosome E1b-V32, both common in East Africa today^28,31^. The individual of East African ancestry was buried with an oil lamp depicting Jupiter-related eagle iconography (Figure 2C; Data S1, section 1), not a common finding in *Viminacium* graves^32^. Isotopic analysis of tooth roots showed that he was also an outlier with respect to dietary habits during childhood (Figure 2B), with elevated δ^15^N and δ^13^C values indicating the likely consumption of marine protein sources^33^, unlike individuals from Pirivoj and other necropolises whose values (Figure 2B) were similar to the Roman-Period population from *Sirmium*^34^ and consistent with a largely C3-based diet with a significant portion of animal protein consumption^33^. Thus, he likely spent his early years elsewhere, possibly in East Africa, the land of his ancestors; while we will never know his whole life story, whether as soldier, slave, merchant, or migrant, it encompassed a long journey that ended with his death in adolescence on the northern frontiers of the Roman Empire.

### From internal to external migration during Late Antiquity

Beginning in the 3^rd^ or 4^th^ century CE, we observe individuals who are admixed with ancestry related to Central/Northern Europeans and Pontic-Kazakh Steppe populations (Figure 4A; Data S2, Table 6). These two ancestry types tend to colocalize in the same individuals, suggesting that the stream of migrants into the Balkans included people who were admixtures of these two sources, although there are some exceptions, such as two contemporaries from *Viminacium,* Pećine necropolis, who can be modeled as having 36-50% steppe-related ancestry without any contribution of Central/Northern European ancestry (Figure 1C and Data S2, Table 6). Individuals bearing these ancestries were buried at the same necropolises (such as Pećine and Više Grobalja at Viminacium) as individuals with predominantly local and Anatolian ancestries, often with overlapping radiocarbon dates, and displayed 42-55% of Balkan Iron Age-related ancestry (Data S2, Table 6). In contrast, only 2 out of 9 males belonged to Y-chromosome lineages found among individuals with a fully local ancestry profile (Data S1, section 2), with the rest belonging to three haplogroups: I1 and R1b-U106 with a strong Northern European distribution, and haplogroup R1a-Z93, which was common in the Steppe during the Iron Age and early 1^st^ millennium CE^35-37^. Such discrepancy between the autosomal and Y-chromosome signals could be explained by a patrilineal social organization such as has been deduced for early Germanic societies^38^ that resulted in the persistence of the incoming male lineages, due to social selection for reproductive success among male offspring from these lineages, and its observation in the admixed individuals in our transect. People with these ancestry profiles present evidence of different dietary patterns, too, as shown by significantly elevated δ^13^C values (p = 0.001) for individuals bearing ancestry from Pontic-Kazakh Steppe groups (Figure 2B), likely pointing to a C4-rich diet^39^.

The appearance of individuals with admixed Central/Northern European and Pontic-Kazakh steppe ancestry inside the Roman Empire in late antiquity reflects the Roman encounter with various trans-frontier populations in this period. Notably, many individuals reflect a prior process of population admixture between these two sources that likely occurred beyond the Roman frontier, perhaps indicative of, e.g., the formation of diverse confederations under Gothic leadership^40^. Furthermore, although the Roman Empire intermittently lost military control of this frontier from the middle of the third century on, it is noteworthy that many individuals with these ancestries appear integrated into Roman society well before the final breakdown of Roman control of the region. This pattern confirms the importance of processes such as migration, recruitment, and settlement (whether sanctioned by the imperial government or not) in the demographic history of the region in late antiquity, a period of intense interaction and exchange across the Danube border^41^. It is also noteworthy that only 3 individuals show >80% ancestry related to Central/Northern European and Pontic-Kazakh steppe groups. Perhaps the fewer samples whose date range falls in the 6^th^ century CE (*n* = 10) obscures the importance of direct migration of large, predominantly Germanic groups into the region. But it is just as important to observe that many individuals belonging to archaeological contexts identified by cultural criteria (Data S1, section 1) as belonging to various Germanic groups reflect a process of admixture with local populations. At Kormadin, for instance, in what has conventionally been identified as a “Gepid” cemetery, out of four individuals tested we identified two who model as completely local Iron Age Balkan ancestry and two, including one child aged 5-7 years, who display local Iron Age Balkan, Central/Northern European and Pontic-Kazakh steppe ancestry.

It is also unexpected to find that Central/North European and Pontic-Kazakh steppe ancestries vanished after 700 CE (95% CI for the sum of these two ancestry proportions = 0-3%) (Figure 4A; Data S2, Table 6). While the relatively small differentiation between Central/North European and Eastern European ancestries could have resulted in the misassignment of small proportions of Central/North European ancestry as Eastern European ancestry, this result is supported by the complete absence (Data S1, section 2) of Y-chromosome lineages clearly associated with Central/North European and Pontic-Kazakh steppe ancestry (I1, R1b-U106 and R1a-Z93) in the 24 individuals in our transect who lived after 700 CE (95% CI for the frequency of those haplogroups = 0-12%). While this absence could reflect unknown sampling bias, it suggests that the population size of incoming Central/North European groups may have been limited as compared to the local Iron Age population, and/or that selective demographic processes—out-migration, differential mortality due to urbanism or military service—acted to prevent a long-lasting demographic impact of these groups.

### Slavic migrations and the formation of the present-day Balkan gene pool

**Figure 3.**
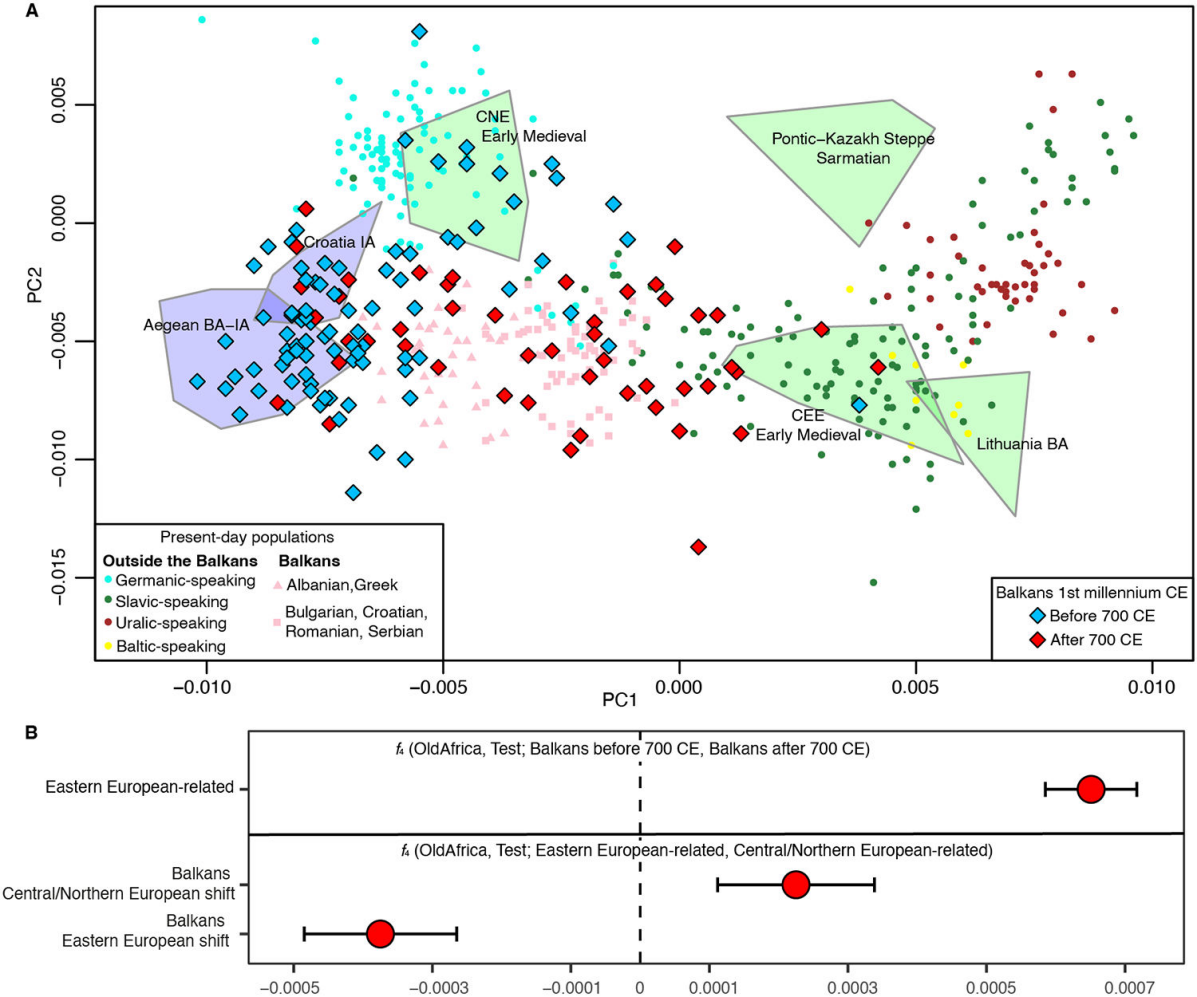
Arrival of ancestry related to Eastern European populations after 700 CE. (A) PCA computed on present-day Central, Northern and Eastern Europeans. Present-day Balkan individuals and ancient individuals were projected onto the PCs. Ancient Balkan individuals are shown as red and blue diamonds and other relevant ancient populations are shown as colored polygons including all individuals in each population. (B) *f*_4_-statistics assessing differential affinities to Central/Northern- and Eastern European-related groups. *Central/Northern European-related* includes individuals from two Langobard-associated cemeteries in Hungary and Northern Italy displaying Central/Northern European-related ancestry (*CNE_EarlyMedieval*) and Bronze and Iron Age individuals from the Netherlands (Data S2, Table 3). *Eastern European-related* includes *CEE_EarlyMedieval* and Bronze Age individuals from Latvia and Lithuania. Test populations are shown in the y-axis. Error bars represent one standard error.

By 700 CE, a new type of ancestry appears across all the Balkan regions covered by our sampling. In a PCA projection onto diverse West Eurasian populations (Figure 1C), these individuals fall at similar positions as the earlier group with Central/Northern European and Pontic-Kazakh steppe-related ancestry. However, we can distinguish their ancestry with a PCA setup more sensitive to recent drift separating Central/Northern and Eastern European populations (Figure 3A). Several Balkan individuals before 700 CE plot close to present-day Central and Northern European Germanic-speaking populations, overlapping individuals from Langobard-associated cemeteries in Hungary and Northern Italy^18^ displaying Central/Northern European-related ancestry (*CNE_EarlyMedieval*). After 700 CE, we observe a clear shift toward present-day Eastern European Slavic-speaking populations in the ancient Balkan transect, a shift mirrored by present-day Balkan populations (Figure 3A). Accordingly, Eastern European-related populations share more alleles (Z = 9.85) with Balkan individuals after 700 CE as compared to before 700 CE (Figure 3B). The differential affinities of Balkan individuals with the strongest Central/Northern European shift in PCA (Z = 1.99) and Balkan individuals with the strongest Eastern European shift in PCA (Z = −3.41), is evident using *f*_4_-statistics of the form *f*_4_ (*OldAfrica, Test; Eastern European-related, Central/Northern European-related*) (Figure 3B). Corroborating these results, *qpAdm* models (Data S1, section 4) with Central/Northern European and Pontic-Kazakh steppe groups yield a very poor fit (p = 2.70 × 10^−15^; Data S2, Table 7) for the group of Balkan individuals with the strongest Eastern European shift, and we were able to obtain a better fit with variable proportions of Balkan Iron Age-related, Anatolian-related and Eastern European-related ancestry (p = 0.049; Data S2, Table 7). As an Eastern European-related proxy, we used a group of early medieval individuals excavated in western Hungary, the Czech Republic, eastern Austria and Western Slovakia (*CEE_EarlyMedieval*). This group fell within the variation of present-day Eastern European Slavic-speaking populations, very close to the Balkan individuals in our dataset with the strongest Eastern European-related shift (Figure 3A; Figure S3).

**Figure 4.**
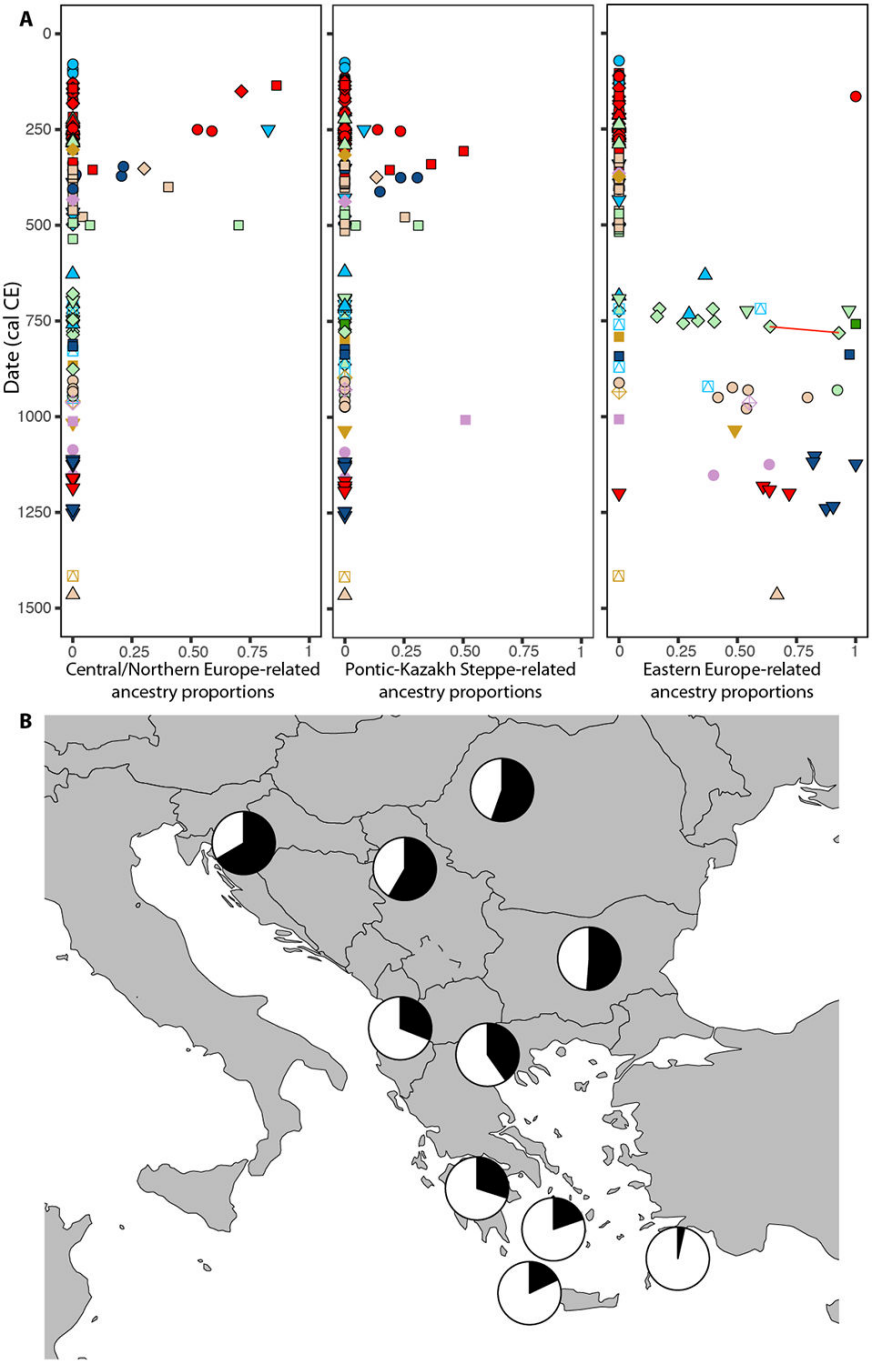
Demographic impact of Migration Period and Early Medieval events. (A) Changes in Central/Northern European, Pontic-Kazakh Steppe and Eastern European-related ancestry proportions between 0-1500 CE, computed with *qpAdm*. A mother and her son are connected through a red line. (B) Proportions of Eastern-European-related ancestry (in black) for present-day Balkan and Aegean populations.

We present evidence that Eastern European ancestry was sporadically present in the Balkans long before the Slavic migrations of late antiquity. Indeed, a woman who probably died in the 2^nd^ or 3^rd^ centuries CE and was buried at Više Grobalja presents unmixed Eastern European ancestry (Figure 4A), offering a remarkable illustration of how small-scale individual percolation into the dynamic economy of the Roman Empire may have preceded larger-scale migration. The vast majority of the individuals with Eastern European ancestry in our dataset appear in the 7th-10th centuries and are of admixed ancestry (Figure 4A); the Slavic migrations started as early as the 6^th^ century^42^, and our dataset may not reflect the early phases, although it provides insights into its dynamics. Out of the seven Balkan individuals with more than 90% East European-related ancestry who were more likely to be migrants, three were females. This finding, together with a ~50/50 ratio of local versus non-local (R1a-Z282, I2a-L621 and Q1a-L715) Y-chromosome lineages (Data S1, section 2), hints at different social dynamics operating during this event as compared to previous periods: here females as well as males make major contributions. We have evidence of the interaction between the two groups at the individual level. At the fortified settlement of Brekinjova Kosa (Bojna, Croatia), two adult men dated to 770-890 cal CE were buried together in the same pit, the younger one with healed skull trauma and with a full Eastern European profile, and the older one with ancestry entirely deriving from Balkan Iron Age populations. Additionally, at the site of Dvorac (Nuštar, Croatia), a woman (Grave 52) with ~90% Eastern European ancestry had a son (Grave 50-A; 64% of this ancestry) (Figure 4A; Data S2, Table 6) with an unsampled man who, like the main group of individuals from the site, must have had ~30% Eastern European ancestry, demonstrating a direct case of incorporation of non-local women that could exemplify some of the social processes at play. The finding of a pair of 10^th^-century twins with southwestern European ancestry at *Timacum Minus* again demonstrates sporadic mobility from far-away regions in the Middle Danube.

To explore whether the Eastern European ancestry signal persisted in present-day Balkan and Aegean populations, we attempted to model present-day groups (Data S1, section 5) by using the same *qpAdm* model used for the ancient individuals after 700 CE with Eastern European-related ancestry. Present-day Serbs, Croats, Bulgarians and Romanians yielded a similar ancestral composition as ancient individuals after 900 CE at sites such as *Timacum Minus*, *Tragurium* or Rudine necropolis at *Viminacium*, with ~50-60% Eastern European-related ancestry admixed with ancestry related to Iron Age Balkan populations and in some cases also a Roman Anatolian contribution (Figure 4B; Data S2, Table 8), implying substantial population continuity in the region over the last 1,000 years. The Eastern European signal significantly decreases in more southern modern groups but it is still present in populations from mainland Greece (~30-40%) and even the Aegean islands (4-20%). This confirms the observations from PCA (Figure 1C and 3A) and previous genetic studies suggesting a substantial demographic impact in the southern Balkan Peninsula^8^ and the Aegean^42^.

## Discussion

Archaeogenetic studies are delivering new evidence that is transforming our understanding of human prehistory and prompting an intense and productive dialogue between geneticists and archaeologists. So far, relatively fewer studies focus on the historical period, that of writing, which requires engagement with written sources in addition to the material evidence. Triangulation of information from history, the archaeological study of material culture, and genetics opens new possibilities for understanding the human past, with each line of evidence not only providing unique information but helping to address biases deriving from other types of analysis. To aid in these goals, the supplement ‘Archaeological Overview’ to this study includes detailed and standardized historical and archaeological information for over twenty sites, along with contextual data for each grave with a newly reported genome. This deeper synthesis of historical, archaeological, and genetic data informs our interpretations, while the detailed site and grave reports allow others to refine our reconstructions or to extend them as future results become available.

The genomic transect of first-millennium individuals from the Balkan Peninsula presented here furnishes new insights into the long-term population dynamics of a region that was both a crucial frontier of the Roman Empire as well as an enduring geographic crossroads between east and west, north and south. The results emphasize the importance of continuing population change in historical times and the long-term shifts in the role of socio-political structures across the 1^st^ millennium. The period of Roman control was dominated by internal migration, with sporadic but increasing long-distance migration from outside the territory of the Roman Empire; this pattern reversed in later centuries, with a relatively larger contribution from populations originating beyond the Danube corridor.

Broadly, our results suggest three phases in the population history of this region in the 1^st^ millennium. First, the high Roman Empire (ca. 1-250 CE) saw the strong impact of Roman culture on the local Iron Age Balkan population. While this process was accompanied by little detectible contribution from populations with ancestry from the Italian Peninsula, there was significant migration by individuals of Anatolian/Eastern Mediterranean ancestry, either directly or through Italy, whose admixture would leave a long trail in later local populations. Meanwhile, militarization and/or economic vitality attracted migrants from further afield both within and beyond the Roman Empire. In some cases at least, the small-scale percolation of individuals preceded large-scale population movements of later centuries.

In the late Roman Imperial period (ca. 250-550) internal migration from within the empire lessened, while the presence of individuals with ancestry originating in populations from beyond the Danube frontier is evident. Admixture was pervasive both among groups originating beyond the frontier (notably Northern/Central Europeans and Pontic-Kazakh Steppe groups) as well as among these groups and the local population. While claims about the identity of individuals or groups have sometimes been made based on material culture discovered in burial contexts, DNA-based ancestry data can reveal the complex role of processes like migration and admixture behind individual and group histories (see above, for the example from Kormadin, where a "Gepid" cemetery certainly included individuals with local Iron Age-Balkan ancestry). The presence of North/Central European ancestry disappears in later periods, suggesting that individuals with this ancestry were relatively few or that historical processes (such as further migration or differential mortality) selectively reduced their contribution in later centuries. For generations, scholars of late Antique history have debated the extent to which the political transitions accompanying the end of Roman rule were fueled by demographic changes and whether these transitions were driven by ethnogenesis or mass migration. Our findings support a nuanced view in which both ethnogenesis and migration were important.

Today, speakers of Slavic languages represent the largest linguistic group in Europe, mainly inhabiting Eastern, Central and Southeastern Europe. Several aspects of their initial arrival in the Balkans are not yet well understood, such as their place of origin and timing, the mechanisms ranging from colonization, invasion, and infiltration, their degree of demographic impact in the region and the underlying reasons with demographic pressures, climate change and depopulation due to the Justinian Pandemic being postulated^42,43^. We document a clear signal of Eastern European-related gene flow in the vast majority of individuals in our dataset after 700 CE (n=49), likely associated with the arrival of Slavic-speaking populations according to historical and archaeological evidence^42^. Due to a gap in our sampling between 500-700 CE, we cannot determine the exact timing of the earliest arrivals, but the detection of individuals with full Eastern European ancestral origin during the 8^th^ and 9^th^ centuries points to a long process encompassing many generations, rather than a short-lived migration event. Unlike the earlier Central/Northern European gene-flow, genomic data are consistent with a major contribution of migrations of both sexes and with a long-lasting strong demographic impact in the region that extends to present-day populations. Nevertheless, our results rule out a complete demographic replacement, as we observe significant proportions of Iron Age Balkan-related and Anatolian-related ancestry across the Medieval period up to the present. These demographic processes of mobility and admixture generated an ancestry cline of present-day Balkan populations with relatively similar ancestry profiles but speaking languages from four different families, i.e. Latin, Slavic, Albanian and Greek, highlighting different cultural processes across the region despite many commonalities in their demographic history. Together, these processes created a regional ancestry profile by the end of the 1^st^ millennium that largely endures across the region.

## Limitations of the Study

Like any historical evidence, this new genetic dataset has limitations. The main one concerns the inherent fragmentary nature of the archaeological record, impacting our study in three ways. First, the prevalence of cremation burial in the first and second century limits the size of the sample in the earliest phase and may bias the results toward a local population more likely to be inhumed. Second, the paucity of sixth-century samples may obscure the presence of populations from Northern/Central Europe who arrived in this later period as well as the earliest phases of the Slavic migrations. Third, urban populations are overrepresented in our study with respect to rural areas, which could be differentially impacted by the demographic processes described in our work. Additional genetic analyses across other Roman frontiers during and after the height of the Empire will help to understand how this ancient phase of globalization shaped the current demographic landscape of three continental regions.

## STAR★Methods:

### Resource Availability

#### Lead Contact

- Further information and requests for resources and reagents should be directed to and will be fulfilled by the lead contact, Iñigo Olalde (inigo.olalde@ehu.eus).

#### Materials availability

- This study did not generate new unique reagents.

#### Data and code availability

- All data needed to evaluate the conclusions in the paper are present in the paper and/or the Supplementary Materials.
- Newly reported ancient sequencing data have been deposited at European Nucleotide Archive (ENA) and are publicly available as of the date of publication with the following accession number PRJEB66422. Haploid genotypes for the 1240k panel for the newly reported ancient individuals, and genotype data for the newly reported present-day individuals are available at https://reich.hms.harvard.edu/datasets.
- This paper does not report original code.
- Any additional information required to reanalyze the data reported in this work paper is available from the lead contact upon request.

### Experimental Model and Study Participant Details

#### Ancient individuals

An extensive description of the archaeological and anthropological context of the ancient individuals analyzed in this study is provided in Data S1, section 1.

#### Present-day individuals

- We collected genetic material from 37 unrelated present-day Serb male individuals from Serbia (*n*=19), Montenegro (*n*=7), Croatia (*n*=5), North Macedonia (*n*=1) and Bosnia and Herzegovina (*n*=5). Serb Individuals were selected according to the following criteria:
  1. Self-declared Serbs leaving on territories of former Yugoslavia where they historically lived.
  2. Speakers of the Serbo-Croatian language.
  3. Belonging to families that are or were in the past of Orthodox religion.
  4. Knowing or still celebrating their family’s Home patron saint, a cultural practice that is characteristic of Serb identity.
- The sample collection and genotyping of the present-day individuals were carried out with the approval and accordance to the supervision of the Ethical committee of the Institute for Molecular Genetics and Genetic Engineering, University of Belgrade (O-EO-29/2021). Participants were informed about the goals of the project and gave informed consent.
- Genomic information for the present-day individuals was obtained by Affymetrix Human Origins Array genotyping of DNA extracted from buccal tissue ^26^, with data quality control performed as described previously ^48^. Data S2, Table 2 shows information of these individuals.

### Method Details

- Direct AMS 14C dates: Radiocarbon dating of a selection of samples was performed at the Pennsylvania State accelerator mass spectrometry radiocarbon laboratory^13^ (Data S2, Table 9). We used the same dental piece or bone as the one subjected to ancient DNA analysis, with the exception of I35012. Dates were calibrated to 2 sigma using OxCal v4.4.2 and the IntCal20 calibration curve^49^. We also obtained δ13C and δ15N values (Data S2, Table 9), informing about dietary habits.
- Ancient DNA laboratory procedures: We selected 146 ancient Balkan individuals for aDNA analysis (Data S1, section 1; Data S2, Table 1). We also selected 4 Early Medieval individuals from Eastern Austria and Western Slovakia, and two Early Medieval individuals from Brandysek (Czech Republic) with previously published shotgun data^50^ (Data S2, Table 1). We performed laboratory work in dedicated clean rooms. The outermost layer of teeth and long bones was removed and powder collected from below the cleaned location to reduce possible exogenous DNA contamination by drilling at low speed to avoid DNA damage from heat^51^. Cochleae were extracted from the temporal bone by sandblasting^52^ and milled. Powder (between 31 and 75 mg per sample) was incubated in lysis buffer and DNA was cleaned and concentrated from one fifth of the lysate following a manual or automated protocol using silica magnetic beads^53^ and Dabney Binding Buffer^54,55^ for manual extraction (Data S2, Table 10). Double-stranded barcoded libraries were prepared with truncated adapters from the extract (corresponding to between 6.2 and 8.4 mg of powder). Libraries were subjected to partial (‘half’) uracil–DNA–glycosylase (UDG) treatment before blunt-end repair to significantly reduce the characteristic damage pattern of aDNA^56,57^. One single-stranded library were prepared using automated scripts following Gansauge *et al.*^58^ (Data S2, Table 10). DNA libraries were enriched for human DNA using probes that target 1,233,013 SNPs (‘1240k capture’^20^) or 1,352,535 SNPs (‘Twist’ BioSciences^21^), and the mitochondrial genome (Data S2, Table 10). Two rounds of capture were performed for the ‘1240k’ reagent and one for the ‘Twist’ BioSciences reagent. Captured libraries were sequenced on an Illumina HiSeq X10 instrument with 2x101 cycles and 2x7 cycles to read out the two indices^59^, or on an Illumina NextSeq 500 instrument with 2x76 cycles and 2x7 cycles to read out the two indices (Data S2, Table 10).
- Bioinformatic data processing: Reads for each sample were extracted from raw sequence data according to sample-specific indices added during wet-lab processing, allowing for one mismatch. Adapters were trimmed and paired-end sequences were merged into single ended sequences requiring 15 base pair overlap (allowing one mismatch) using a modified version of *SeqPrep 1.1* (https://github.com/jstjohn/SeqPrep) which selects the highest quality base in the merged region. Unmerged reads were discarded prior to alignment to both the human reference genome (hg19) and the RSRS version of the mitochondrial genome using the ‘samse’ command in *bwa* (version 0.6.1)^60^. Duplicates were removed based on the alignment coordinates of aligned reads, as well as their orientation. Libraries were sequenced to saturation across multiple sequencing lanes where necessary, with complexity metrics established using *preseq*^61^, merging where necessary. The computational pipelines are available on GitHub (https://github.com/dReichLab/ADNA-Tools, https://github.com/dReichLab/adna-workflow). A total of ten samples failed nuclear capture (Data S2, Table 1), yielding 142 ancient individuals with genome-wide data (136 individuals from the Balkans). Subsequent authenticity of ancient DNA was established using several criteria: we discarded from further analysis libraries with a rate of deamination at the terminal nucleotide below 3%. We computed the ratio of X-to-Y chromosome reads, estimated mismatch rates to the consensus mitochondrial sequence, using *contamMix-1.0.10*^62^ and ran X-chromosome contamination estimates using *ANGSD*^63^ in males with sufficient coverage (Data S2, Table 1). Libraries with evidence of contamination or without a minimum of 20,000 SNPs with at least one overlapping sequence were discarded from genome-wide analyses. At this stage, 13 individuals were excluded, keeping 129 individuals (123 Balkan individuals) for genome-wide analyses (Data S2, Table 1). For this study, we restricted all our analysis to the 1,233,013 SNPs in common between 1240k and Twist reagents, as well as the mitochondrial genome.

### Quantification and Statistical Analysis

- Mitochondrial haplogroup determination: Reads mapped to mitochondrial reference genome were used to determine mtDNA haplogroups (using sequences with mapping quality (MAPQ) ≥ 30 and base quality ≥ 30). A consensus sequence was first determined using bcftools and SAMTools^60^ using a majority rule and requiring a minimum coverage of two. These consensus sequences were then used to determine mitochondrial haplogroups (Data S2, Table 1) using *HaploGrep2* based on phylotree (mtDNA tree build 17)^64,65^.
- Y-chromosome haplogroup determination: To determine the Y-chromosome lineages from the male ancient individuals, we annotated the path of derived mutations (Data S2, Table 1) using the nomenclature of Yfull 8.09 (https://www.yfull.com/), following the same procedure as described in Lazaridis et al. (2022)^25^. We also annotated the haplogroup name associated to the most derived mutation for each sample using the nomenclature of the International Society of Genetic Genealogy (http://www.isogg.org; version 15.73). We comment about the Y-chromosome temporal patterns in Data S1, section 2.
- Kinship analysis: We tested for kinship relationships among pairs of newly reported individuals included in our study. For this purpose, we used the Relationship Estimation from Ancient DNA (READ) program implemented by Monroy Kuhn *et al.*^66^, which can infer family relationships up to second degree even from samples with very low coverage (Data S2, Table 4). Degrees of kinship classification in a population must be independent of within-population diversity, and thus the proportion of non-matching alleles (P0) needs to be normalised before classifying relationships between pairs of individuals. This was achieved by using the expected value for a randomly chosen pair of unrelated individuals from the same population. Both window size and median pairwise P0 default options were used. We found five close kinship relationships: four first-degree pairs, one second-degree relative pair, and a pair of identical twins (Data S1, section 3).
- Determination of molecular sex: To determine the molecular sex of the ancient individuals, we computed the ratio of sequences mapping to Y-chromosome SNP targets to the sum of sequences mapping to X and Y-chromosome SNP targets. As female individuals lack Y chromosome the ratio should be close to 0, whereas male individuals’ ratio should be significantly higher. We used a thresholding in this ratio of <0.03 for classifying a sample as female and >0.35 for male. Results of the determination can be found in Data S2, Table 1.
- Determination of aneuploidies: No apparent aneuploidies were identified in any of the newly reported individuals. Aneuploidies were studied by computing the mean coverage at 1240k SNPs of each chromosome divided by the mean coverage at 1240k SNPs of all autosomes, which should be around 1 for autosomes if no aneuploidies are present; 1 for females and 0.5 for males at the X chromosome if no aneuploidies are present, and 0 for females and 0.5 for males if no aneuploidies are present. The X/autosomes and Y/autosomes coverage ratios are included in Data S2, Table 11.
- Determination of runs of homozygosity (ROH): We assessed the possible presence of runs of homozygosity (ROHs) in the newly reported ancient individuals by applying the method described in Ringbauer *et al*.^67^. Only one ROH segment >20 cM was found in the whole dataset (Data S2, Table 1), indicating the absence of close-kin unions between the parents of the newly reported individuals.
- Genome-wide analysis datasets: To study the genetic ancestry of the newly sequenced individuals we built two datasets: The ‘HO’ dataset containing 6695 present-day individuals from worldwide populations^26,48,68-70^, 37 newly reported present-day Serbs, all genotyped on the Human Origins Array, together with 3690 present-day individuals with whole-genome data from the SGDP, HGDP and 1000 genomes datasets^48,68,71-76^, a set of 624 relevant ancient individuals (Data S2, Table 3) with genome-wide data from previous publications^15,18,19,50,68,77-93^, and our newly sequenced ancient individuals from the Balkans and adjacent regions. We kept 591,642 SNPs resulting from the intersection between the Human Origins array and the 1240k capture. Second, the ‘1240k’ dataset contained the same individuals as the ‘HO’ dataset but excluded present-day individuals genotyped on the Human Origins array. We kept 1,054,671 autosomal SNPs, excluding SNPs of the 1240k array that are been specifically included based on their functional effects or located on the sex chromosomes. In both datasets, for each individual we randomly sampled one allele at each SNP position to represent the data for that individual.
- Principal Component Analysis: We performed Principal Component Analysis (PCA) using the ‘smartpca’ program in EIGENSOFT (version 7.2.1). We projected ancient individuals onto the components computed on present-day individuals with “lsqproject:YES” and “shrinkmode:YES”^24,94^. We ran five different PCAs: 1. One for which PCs were computed on the HO dataset using 1036 present-day West Eurasian individuals genotyped on the Human Origins array (Figure 1C; Figure S1). We used this PCA to determine affinities of our samples with Near-Eastern/ European populations. 2. One for which PCs were computed using 1314 present-day West Eurasian and African individuals genotyped on the Human Origins array ^26^ (Figure S2A). We used this PCA to investigate the genomic affinities of newly reported ancient individual plotting outside West Eurasian genetic variation. 3. One for which PCs were computed on the HO dataset using 429 present-day Central, Northern and Eastern Europeans genotyped on the HO array (Figure S3). We designed this PCA to reveal more recent shared drift that could better separate Central-Northern European populations from Central-Eastern European populations. 4. One for which PCs were computed on the HO dataset using 1180 present-day West Eurasian and East Eurasian (North, East and Southeast Asia) individuals genotyped on the Human Origins array ^26^ (Figure S2B). We used this PCA to investigate the presence of East Eurasian-related ancestry in our newly reported individuals. 5. One for which PCs were computed on the 1240k dataset using 161 present-day West Eurasians from eight populations (Russian, Orcadian, French, Tuscan, Sardinian, Basque, Adygei, Druze) from the HGDP set of globally diverse populations ^71^. This PCA (Figure S4) uses twice as many SNPs as the previous ones as we used whole genome sequencing data, reducing noise especially for individuals with low-quality data. The trade-off is the reduced present-day sampling density across space as compared to HO dataset.
- *f*_4_-statistics: We computed *f*_4_-statistics in AdmixTools v.6. (https://github.com/dReichLab/AdmixTools)^26,95^. using the program *qpDstat* and f4mode: YES. Standard errors were computed with the default jackknife approach.
- *qpAdm* admixture modelling of ancient individuals: We modelled the ancestry of the newly reported Balkan individuals, as well as 15 previously published^25^ individuals from similar historical and geographic context (Data S2, Table 1), using the *f-*statistics framework implemented in the *qpAdm* software from AdmixTools v.6. (https://github.com/dReichLab/AdmixTools)^26,95^. We performed the analyses on the ‘1240k dataset’ and set the “allsnps: YES” option. When choosing populations to act as sources and outgroups in the models, we avoided including individuals with shotgun data and/or without UDG treatment whenever possible, to avoid biases that appear when different types of data are co-analyzed ^21^. Given the very high ancestry heterogeneity observed in PCA, even within the same archaeological sites and time periods, we decided not to group individuals for analysis with the goal of allowing as much granular analysis as possible (Data S1, section 4). The disadvantage of this strategy is that it decreases the power to reject non-fitting models, as compared to an approach where samples are grouped into populations or clusters whose ancestry is then modelled. To mitigate this problem of reduced statistical power, we excluded individuals with fewer than 40,000 SNPs for this *qpAdm* analysis and merge the data for the two individuals who were genetically identical (I15538 and I15539).
- *qpAdm* admixture modelling of present-day Balkan and Aegean populations: With the knowledge gained through the ancestry analyses of the ancient Balkan individuals, we modelled the ancestry of present-day populations from the Balkans and the Aegean, using *qpAdm* (Data S1, section 5). This analysis was performed on the ´HÓ dataset, after filtering out 366,668 SNPs (224,207 SNPs remained) known to produce biases when co-analyzing 1240k data (most of our ancient samples) with other types of data^21^, in this case the present-day groups genotyped on Human Origins array.

## Supplementary Material

Figure S3 **Figure S3. PCA with ancient samples and present-day Balkan populations projected onto the PCs computed on present-day Central, Northern and Eastern Europeans, related to** Figure 3. This PCA corresponds to that in Figure 3A with a more detailed color scheme.

Figure S1 **Figure S1. PCA with ancient samples projected onto the PCs computed on present-day West-Eurasian individuals, related to** Figure 1. This PCA is the zoom-out version of main text Figure 1C to fully visualise the West-Eurasian population structure.

Figure S2 **Figure S2. PCAs with ancient samples projected onto the PCs computed on present-day West-Eurasian populations and additional individuals, related to** Figure 1. (A) Including present-day African individuals. (B) Including present-day East Eurasian individuals.

Figure S4 Figure S4. PCA with the ancient samples projected onto the PCs computed on 161 present-day West Eurasians from eight populations (Russian, Orcadian, French, Tuscan, Sardinian, Basque, Adygei, Druze), using the 1240k dataset, related to Figure 1.

Data S1 Reference list

Data S2 **Data S2**: Data tables, related to STAR Methods.

Data S1 **Data S1**: Supplementary information: archeological and historical context of the archeological sites reported in this study, additional statistical analysis and discussion. Related to STAR Methods.

## Inclusion and diversity:

We support inclusive, diverse, and equitable conduct of research.
