# Supplementary figures and images for: A Genetic History of the Balkans from Roman Frontier to Slavic Migrations
Source: Cell. Author manuscript; Available in PMC 2023 Dec 27. (PMC10752003; doi:10.1016/j.cell.2023.10.018)

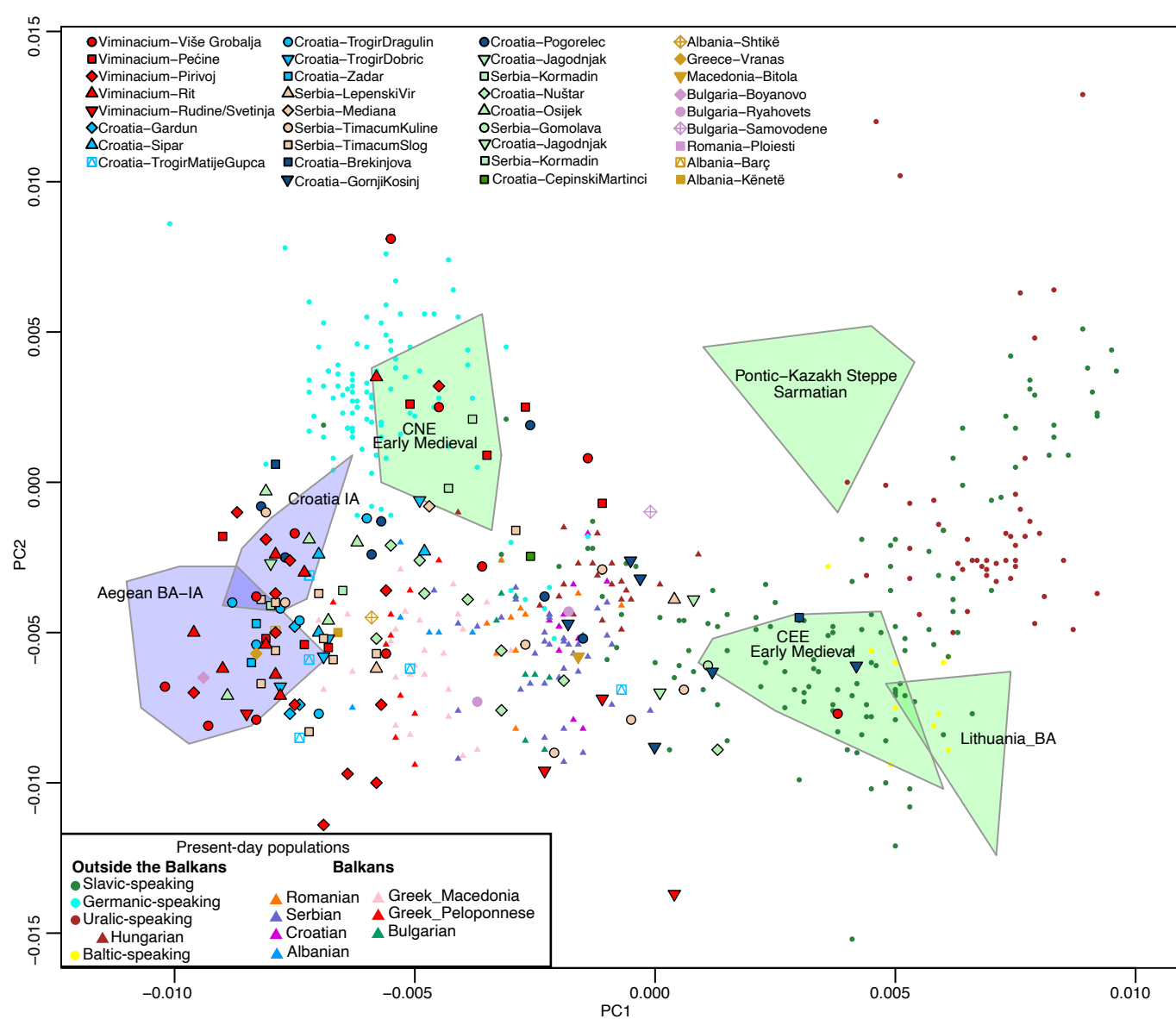

Supplement: Figure S3 — PCA with ancient samples and present-day Balkan populations projected onto the PCs computed on present-day Central, Northern and Eastern Europeans, related to Figure 3. This PCA corresponds to that in Figure 3A with a more detailed color scheme. [file NIHMS1944038-supplement-Figure_S3.pdf]

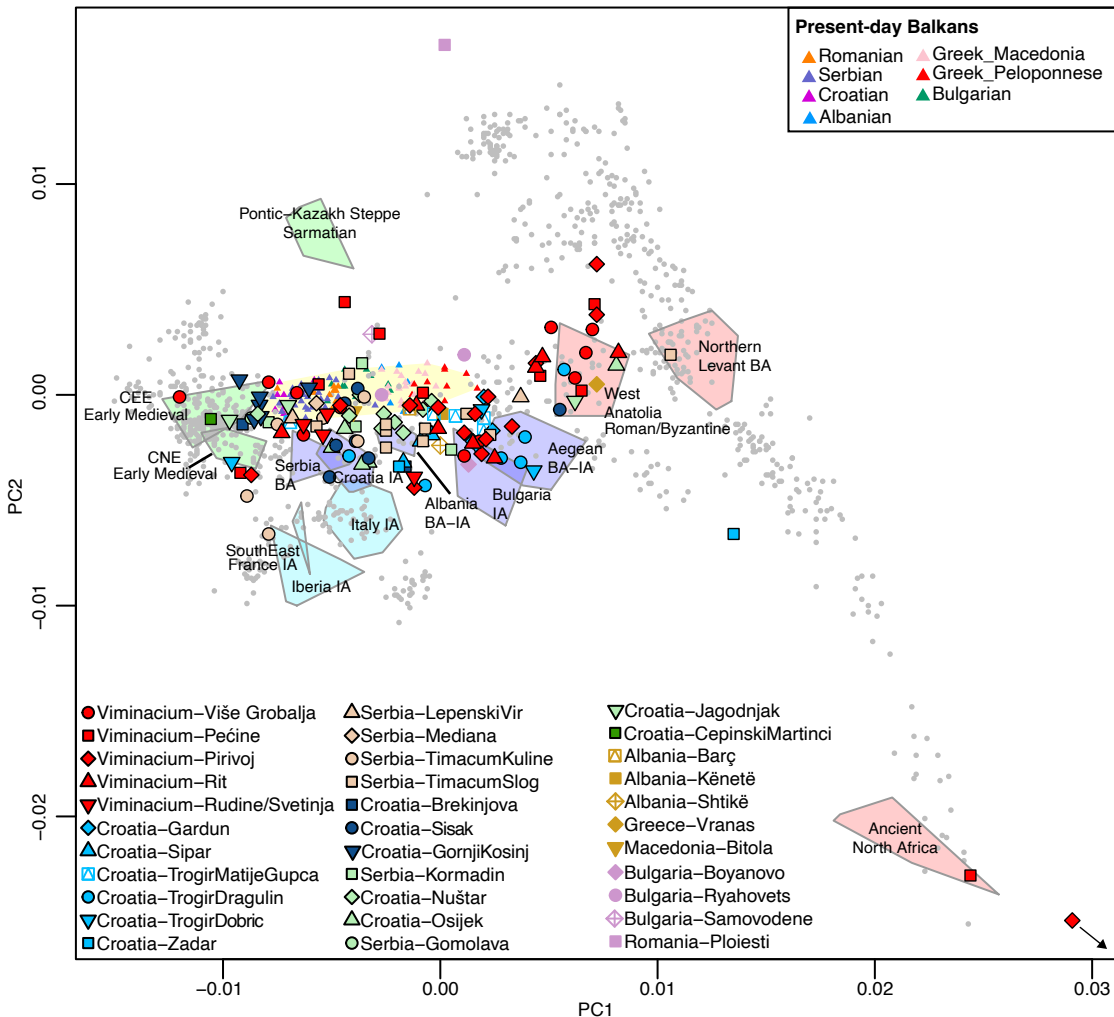

Supplement: Figure S1 — PCA with ancient samples projected onto the PCs computed on present-day West-Eurasian individuals, related to Figure 1. This PCA is the zoom-out version of main text Figure 1C to fully visualise the West-Eurasian population structure. [file NIHMS1944038-supplement-Figure_S1.pdf]

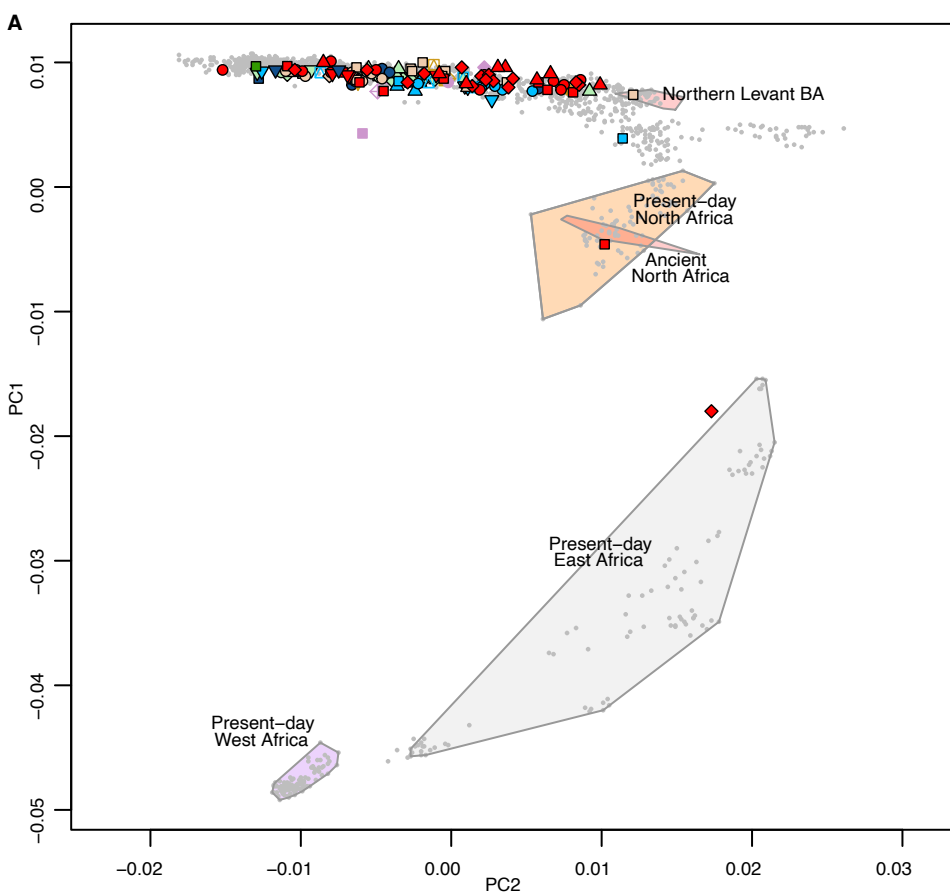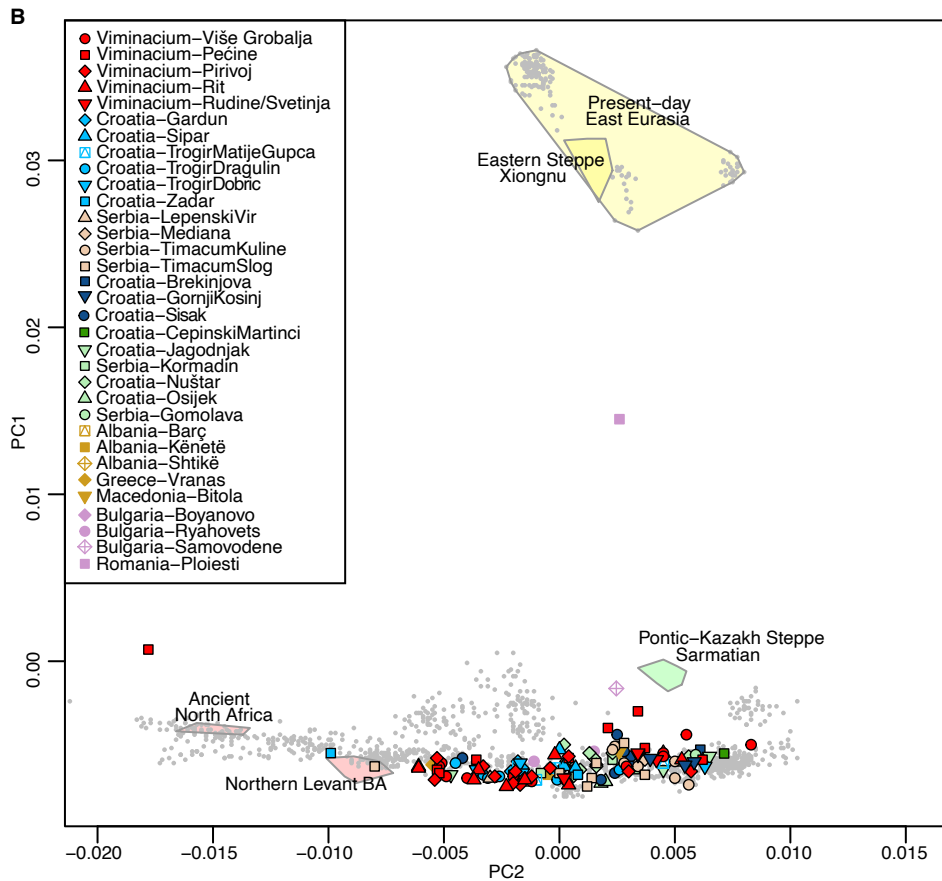

Supplement: Figure S2 — PCAs with ancient samples projected onto the PCs computed on present-day West-Eurasian populations and additional individuals, related to Figure 1. (A) Including present-day African individuals. (B) Including present-day East Eurasian individuals. [file NIHMS1944038-supplement-Figure_S2.pdf]

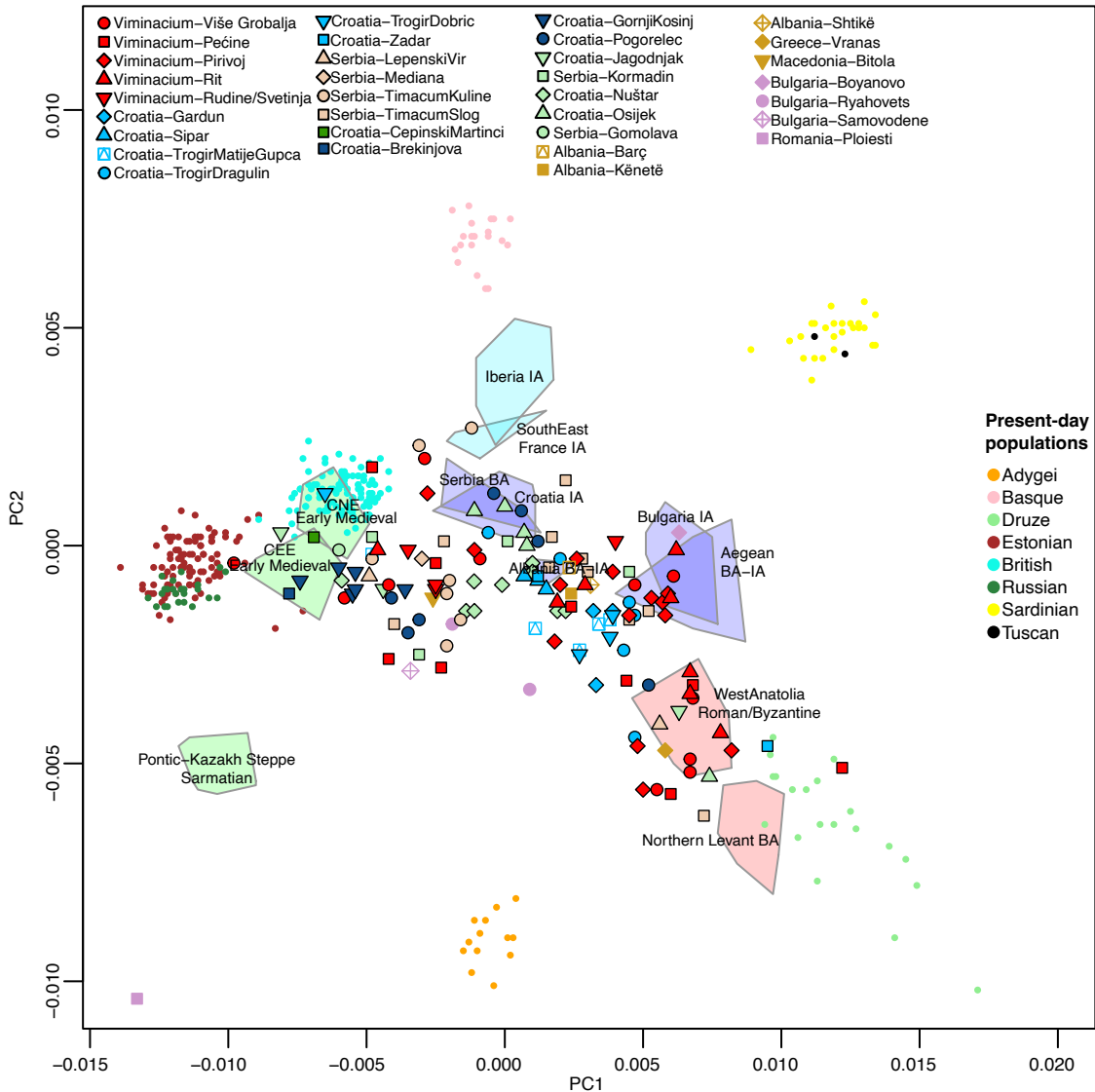

Supplement: Figure S4 — PCA with the ancient samples projected onto the PCs computed on 161 present-day West Eurasians from eight populations (Russian, Orcadian, French, Tuscan, Sardinian, Basque, Adygei, Druze), using the 1240k dataset, related to Figure 1. [file NIHMS1944038-supplement-Figure_S4.pdf]
