# Supplementary material for: A Genetic History of the Balkans from Roman Frontier to Slavic Migrations
Source: Cell. Author manuscript; Available in PMC 2023 Dec 27. (PMC10752003; doi:10.1016/j.cell.2023.10.018)
Supplement: Data S1 Reference list [file NIHMS1944038-supplement-Data_S1_Reference_list.docx]

**Suplemental Reference list**

1. Skoglund, P., Storå, J., Götherström, A., & Jakobsson, M. (2013). Accurate sex identification of ancient human remains using DNA shotgun sequencing. *Journal of Archaeological Science*, *40*(12), 4477–4482.

2. Bartík, J., & Šefčáková, A. (2004). Hrob so šálkou protoúnětickej kultúry z Blatného, okr. Bratislava-vidiek, Slovensko. *Zborník K Poctě Vladimíru Podborskému, FF Masaryk University in Brno, Brno,* 235–244.

3. Madiraca, V., Koprivnjak, V., Miletić, M., Zubin Ferri, T., & Bekić, L. (2017). Early medieval finds from the Brekinjova Kosa archaeological site (results of excavations in 2011 and 2015). *Archaeologia Adriatica*, *11*(1), 145–215.

4. McCormick, M. (2001). *Origins of the European economy: communications and commerce AD 300-900*. Cambridge University Press.

5. Kalafatić, H. (2009). Zaštitna istraživanja lokaliteta Čepinski Martinci-Dubrava na trasi autoceste Beli Manastir-Osijek-Svilaj 2007. i 2008. g. *Annales Instituti Archaeologici*, *1*, 20–25.

6. Krznar, S., & Tkalčec, T. (2015). The identity of the community and the identity of the individual: the burial of the deceased within the settlement in the Middle Ages in Northern Croatia. *Ruralia XI-Religious Places, Cults and Rituals in Medieval Rural Environment*, *24*.

7. Vyroubal, V., & Bedić, Ž. (2015). Rezultati antropološke analize ljudskog osteološkog materijala s nalazišta Donji Miholjac - Đanovci i Čepinski Martinci. *Zagreb, Antropološki Centar Hrvatske Akademije Znanosti i Umjetnosti*, *in press*.

8. Sanader, M., & Tončinić, D. (2010). Gardun-The Ancient Tilurium. *Finds of the Roman Military Equipment in Croatia (Zagreb)*, 33–53.

9. Sanader, M., Demicheli, D., & Milićević Bradač, M. (2013). A “Poet” in the military camp at Tilurium, in: Sanader M., Rendić-Miočević A., Tončinić D., Radman-Livaja I. (Eds.), Proceedings of the XVII Roman military equipment conference. *Filozofski Fakultet Sveučilišta u Zagrebu / Arheološki Muzej u Zagrebu*, 411–433.

10. Ritterling, E. (1925). Legio. *Re­al-Encyklopädie Der Classischen Altertumswissenschaft*, *12*(2), 1329–1829.

11. Sanader, M., Tončinić, D., Šimić-Kanaet, Z., Ivčević, S., Buljević, Z., & Šeparović, T. (2017). Tilurium IV: arheološka istraživanja 2007.-2010. *Arheološki Zavod Filozofskog Fakulteta u Zagreb*.

12. Zaninović, M. (2007). Beneficiarii consularis na području Delmata. *Prilozi Instituta Za Arheologiju u Zagrebu*, *24*, 181–184.

13. Sanader, M., Tončinić, D., Buljević, Z., Ivčević, S., & Šeparović, T. (2014). *Tilurium III: istraživanja 2002.-2006. godine*. Filozofski fakultet, Zavod za arheologiju.

14. Sanader, M. (2003). Tilurium I. Istraživanja – Forschungen 1997. – 2001. *Dissertationes et Monographiae* , *4*.

15. Šlaus, M., & Novak, M. (2014). Antropološka analiza ljudskog osteološkog materijala. In *Tilurium III. Istraživanja 2001.-2006. godine* (Vol. 6, pp. 117–131). Dissertationes et Monographiae.

16. Buljević, Z. (2014). Stakleni inventar. In *Tilurium III. Istraživanja 2001.-2006. Godine* (Vol. 6, pp. 225–292). Dissertationes et Monographiae.

17. Jovanović, M. (2015). Gomolova, Hrtkovci — Vinčanska nekropola. *Rad Muzeja Vojvodine*, *57*, 7–60.

18. Borić, D. (2009). Absolute Dating of Metallurgical Innovations in the Vinča Culture of the Balkans, in: Metals and Societies. *Studies in Honour of Barbara S. Ottaway*, 191–245.

19. Mathieson, I., Alpaslan-Roodenberg, S., Posth, C., Szécsényi-Nagy, A., Rohland, N., Mallick, S., Olalde, I., Broomandkhoshbacht, N., Candilio, F., Cheronet, O., Fernandes, D., Ferry, M., Gamarra, B., Fortes, G. G., Haak, W., Harney, E., Jones, E., Keating, D., Krause-Kyora, B., … Reich, D. (2018). The genomic history of southeastern Europe. *Nature*, *555*(7695), 197–203. https://doi.org/10.1038/nature25778

20. Horvat, Z. (2003). O nekim osobinama sakralne arhitekture u Lici nakon protjerivanja Turaka. *Senjski Zbornik: Prilozi Za Geografiju, Etnologiju, Gospodarstvo, Povijest i Kulturu*, *30*(1), 109–147.

21. Šarić, I., & Šmalcej, M. (1988). Gornji Kosinj. *Rekognosciranje i Sondiranja. Arheološki Pregled*, 220–222.

22. Petrinec, M. (2009). *Groblja od 8. do 11. stoljeća na području ranosrednjovjekovne hrvatske države*. Muzej Hrvatskih Arheoloških Spomenika.

23. Gusar, K., & Vujević, D. (2013). Grob 75 s lokaliteta Pakoštane-Crkvina. *Archaeologia Adriatica*, *7*(1), 271–300.

24. Tresić Pavičić, D. (2016). Jagodnjak-Krčevine-Selska bara (AN 7). *Hrvatski Arheološki Godišnjak*, *12*, 39–41.

25. Dugonjić, A., Dinko  Tresić, P., & Mario  Novak. (2022). The early medieval cemetery in Jagodnjak (Croatian Baranja) –First results of the archaeological excavations and anthropological analysis. In A. Rapan Papeša & A. Dugonjić (Eds.), *Avari i Slaveni: Dvije strane pojasnog jezičca. Avari na sjeveru i jugu kaganata / Avars and Slavs: Two sides of a belt strap end: Avars on the North and South of the Khaganate* (pp. 26–61). Arheološki muzej u Zagrebu.

26. Bunčić, M. (2020). *Permanent Exhibition of the Medieval Collection. Guide.* Archaeological Museum in Zagreb.

27. Šeper, M. (1952). Neolitičko naselje na Kormadinu. *Arheološki Vestnik*, *3*(1), 24–98.

28. Dimitrijević, D. (1960). Gepidska nekropola „Kormadin“ kod Jakova. *Rad Vojvođanskih Muzeja* , *9*, 5–50.

29. Bulatović, A. P., Kapuran A.N, & Strugar, , N.I. (2010). Neolitski stratum na lokalitetu Kormadin u Jakovu – sondažno iskopavanje 2008. *Godišnjak Grada Beograda* , *60*, 1–32.

30. Roksandic, M. (1999). *Transition from Mesolithic to Neolithic in the Iron Gates gorge: Physical anthropology perspective*. Simon Fraser University Burnaby, BC.

31. Srejović, D. (1969). *Lepenski vir: nova praistorijska kultura u Podunavlju*. Srpska književna zadruga.

32. Bonsall, C., Vasić, R., Boroneanţ, A., Roksandic, M., Soficaru, A., McSweeney, K., Evatt, A., Aguraiuja, Ü., Pickard, C., & Dimitrijević, V. (2015). New AMS 14C dates for human remains from Stone Age sites in the Iron Gates reach of the Danube, southeast Europe. *Radiocarbon*, *57*(1), 33–46.

33. Balen-Letunić, D. (2004). Japodi, in: D. Balen-Letunić, D. (ed.) Ratnici na razmeđu istoka i zapada. Starije željezno doba u kontinentalnoj Hrvatskoj. . *Arheološki Muzej Zagreb*, 212–254.

34. Balen-Letunić, D. (2000). Japodske nekropole s ogulinskog područja. *Vjesnik Arheološkog Muzeja u Zagrebu* , *32*(33), 23–61.

35. Vasić, M., Milošević, G., Gavrilović Vitas, N., & Crnoglavac, V. (2016). Constantine’s villa at Mediana. *Niš: Narodni Muzej*.

36. Mikić, Ž. (2006). Mediana and it’s Medieval Population-an anthropological study. *Serbian Academy of Science and Arts-Macedonian Academy of Science and Arts Publication*, *Special Issue “Homeage to Milutin Garašanin,”* 647–654.

37. Veeramah, K. R., Rott, A., Groß, M., Van Dorp, L., López, S., Kirsanow, K., Sell, C., Blöcher, J., Wegmann, D., Link, V., Hofmanová, Z., Peters, J., Trautmann, B., Gairhos, A., Haberstroh, J., Päffgen, B., Hellenthal, G., Haas-Gebhard, B., Harbeck, M., & Burger, J. (2018). Population genomic analysis of elongated skulls reveals extensive female-biased immigration in Early Medieval Bavaria. *Proceedings of the National Academy of Sciences of the United States of America*, *115*(13), 3494–3499. https://doi.org/10.1073/pnas.1719880115

38. Hakenbeck, S. (2009). *’Hunnic’modified skulls: physical appearance, identity and the transformative nature of migrations*.

39. Mikić, Ž. (2008). Deux nécropoles de la Grande migration des peuples á Viminacium. *Balcanica*, *48*, 45–50.

40. Микић, Ж. (2010). Вештачи дефомисане лобанје са Медијане-антрополошки осврт. *Гласник Српског Археолошког Друштва*, *26*, 1693–173.

41. Farkaš, Z. (2019). Záver kultúry ľudu so starou lineárnou keramikou. Obj. 114/86 v Bratislave – Mlynskej doline. . *Zborník Slovenského Národného Múzea – Archeológia*, *29*, 7–29.

42. Egyházy-Jurovská, B., & Farkaš, Z. (1984). 1985: Záchranný výskum v Mlynskej doline v Bratislave. *Archeologické Výskumy a Nálezy Na Slovensku v Roku*, 80–81.

43. Farkaš, Z. (2002). Nálezy ľudských pozostatkov v prostredí kultúry ľudu s lineárnou keramikou na Slovensku. *Archeologické Rozhledy*, *54*(1), 23–43.

44. Rapan Papeša, A., Kenéz, Á., & Pető, Á. (2015). The archaeobotanical assessment of grave samples from the Avar Age cemetery of Nuštar (Eastern Croatia). *Prilozi Instituta Za Arheologiju u Zagrebu* , *32*, 261–288.

45. Dugonjić, A., & Rapan Papeša, A. (2019). *Avari i Slaveni/Avars and Slavs*. Zagreb.

46. Rapan Papeša, A. (2014). Keramički i olovni pršljenci za vretena iz avarodobnih grobova na lokalitetu Nuštar–Dvorac. *Prilozi Instituta Za Arheologiju u Zagrebu*, *31*, 159–180.

47. Grömer, K., & Rapan Papeša, A. (2015). Simple cloth and stamped leather: organic finds from the Avar graveyard in Nuštar (Eastern Croatia). *Vjesnik Arheološkog Muzeja u Zagrebu*, *48*(1), 51–83.

48. Vidal-Ronchas, R., Rajić Šikanjić, P., Premužić, Z., Rapan Papeša, A., & Lightfoot, E. (2019). Diet, sex, and social status in the Late Avar period: stable isotope investigations at Nuštar cemetery, Croatia. *Archaeological and Anthropological Sciences*, *11*, 1727–1737.

49. Premužić, Z., Rajić Šikanjić, P., & Rapan Papeša, A. (2017). Bioarheološka analiza avarodobnog groblja u Nuštru, in: Tončinić D. (ed.). *Izdanja Hrvatskog Arheološkog Društva 31: Arheologija Na Dunavu. Hrvatsko Arheološko Društvo*, 117–122.

50. Premužić, Z., Rajić Šikanjić, P., & Rapan Papeša, A. (2016). A case of Avar period trepanation from Croatia. *Anthropological Review*, *79*, 471–482.

51. Zábojník, J. (1991). Seriation von Gürtelbeschlaggarnituren aus dem Gebiet der Slowakei und Österreichs (Beitrag zur Chronologie der Zeit Des Awarischen Kaganats). In Z. Čilinská (Ed.), *K problematike osídlenia srednodunajskej oblasti vo včasnom stredoveku* (pp. 219–321). Archeologický ústav Slovenskej akadémie vied.

52. Čilinská, Z. (1975). *Frauenschmuck aus dem 7.-8. Jahrhundert im Karpatenbecken*. *XXIII*, 63–97.

53. Daim, F. (1987). Das awarische Gräberfeld von Leobersdorf, NÖ. Studien zur Archäologie der Awaren 3. *Veröffentlichung Der Kommission Für Frühmittelalterforschung, Denkschriften Der Österreichischen Akademie Der Wissenschaften*, *2*(1194), 10.

54. Rapan Papeša, A. (2018). Magic or Practicality?“Antiquities” in Avar Period Graves in Eastern Croatia. *Life and Death InMedieval and Early Modern Times. Proceedings of the 5th International Scientific Conference of Mediaeval Archaeology of the Institute of Archaeology Zagreb, 6th and 7th June*, 17–31.

55. Leleković, T. (2009). Osijek – Trg bana Jelačića. *Hrvatski Arheološki Godišnjak* , *5*(2008), 45–51.

56. Leleković, T. (2020). Elija Mursa: novo čitanje grada. *Elija Mursa: Novo Čitanje Grada*, *19*, 77–161.

57. McCormick, M. (2015). Tracking mass death during the fall of Rome’s empire (I). *Journal of Roman Archaeology*, *28*, 325–357.

58. Drnić, I., & Groh, S. (2018). Excavations and geophysical prospection of the Iron Age and Roman period site at Sisak–Pogorelac position (2012–2017). *Vjesnik Arheološkog Muzeja u Zagrebu*, *51*(1), 67–140.

59. Drnić, I. (2018). Segestica and Siscia: From the periphery of the Empire to a provincial center. In I. Drnić (Ed.), *Segestica and Siscia: From the periphery of the Empire to a provincial center* (Vol. 16, pp. 7–21). Archaeological Museum in Zagreb.

60. Lolić, T. (2022). *Urbanism of Roman Siscia: Interpretation of Historical and Modern Maps, Drawings and Plans*. Archaeopress Publishing Ltd.

61. Lolić, T. (2003). Colonia Flavia Siscia. *V: The Autonomous Towns of Noricum and Pannonia. Pannonia I. Situla*, *41*, 131–152.

62. Baćani, I., Škrgulja, R., & Tomaš Barišić, T. (2018). *Necropolises of Siscia, exhibition catalogue*. Sisak City Museum.

63. Friesinger, H. (1972). *Frühmittelalterliche Körpergräber aus Pottenbrunn, Stadtgemeinde St. Pölten, NÖ.* *51*, 113–189.

64. Friesinger, H. (1966). *Fundberichte aus Österreich*. *9*, 30–33.

65. Jungwirth, J., & Windl, H. (1973). *Fundberichte aus Österreich*. *12*, 130–134.

66. Spindler, P., & Windl, H. (1974). *Fundberichte aus Österreich* . *13*, 136–139.

67. Windl, H. (1975). *Fundberichte aus Österreich* . *14*, 178.

68. Fabrizii-Reuer, S., & Reuer, E. (2001). Das frühmittelalterliche Gräberfeld von Pottenbrunn, Niederösterreich. Anthropologische Auswertung. *Mitteilungen Der Prähistorischen Kommission*, *40*.

69. Steckel, R. H., Larsen, C. S., Roberts, C. A., & Baten, J. (2019). The European History of Health Project: Introduction to Goals, Materials, and Methods. In R. H. Steckel, C. S. Larsen, C. A. Roberts, & J. Baten (Eds.), *The Backbone of Europe.* (pp. 1–10). Cambridge University Press .

70. Gausterer, C., Stein, C., & Teschler-Nicola, M. (2015). Erster genetischer Nachweis von Lepra im frühmittelalterlichen Österreich. *Wiener Medizinische Wochenschrift*, *165*, 126–132.

71. Teschler-Nicola M. (2009). First evidence of Leprosy in Early Mediaeval Austria. *5th International Anthropological Congress of A. Hrdlicka, Praha and Humpolec, Czech Republic, Anthropologie* , *XLVII*(2–3).

72. Teschler-Nicola, M., & Gausterer, C. (2009). Ein erster Nachweis von Lepra aus dem frühmittelalterlichen Österreich. *Archäol Österr*, *20*, 25–27.

73. Teschler-Nicola, M., Harkins, K. M., & Schamall, D. (2007). Multiple Cases of Inherited Metabolic Disorder (Mucopolysaccharidosis) in a Mediaeval Population from Pottenbrunn, Lower Austria. *34th Annual Meeting of the PPA (North America), Philadelphia, Abstractbook* , 28.

74. Petković, S. (2005). *Roman and Medieval necropolis in Ravna near Knjaževac*. Archaeological Institute of Belgrade.

75. Petković, S., & Ilijić, B. (2013). Votive altar of Lucius Petronius Timachus. *Starinar*, *63*, 53–72.

76. Hirt, A. M. (2010). *Imperial mines and quarries in the Roman world: organizational aspects 27 BC-AD 235*. OUP Oxford.

77. Petković, S., & Miladinović-Radmilović, N. (2014). Military graves from the late Roman necropolis at Slog in Ravna (Timacum Minus). *Starinar*, *64*, 87–130.

78. Haury, J., & Wirth, G. (1964). Procopius of Cesaria, Buildings. In B. G. Teubner (Ed.), *Procopii Caesariensis Opera omnia: Vol. 4.4* (pp. 123.45-124.21).

79. Miladinović-Radmilović, N., Bizjak, D., & Vulović, D. (2014). Early Medieval necropolis on the site Timacum Minus-Kuline. *Vesnik, Journal for History, Muzeology and Art,* *41*, 9–31.

80. Milošević Zakić, B. (2022). Mreže i udice. Eksploatacija mora u doba antike i ranog srednjeg vijeka. Reti e ami da pesce. Sfruttamento del mare in eta’ romana e altomedievale. . In *Katalog izložbe/Catalogo della mostra.* Muzej grada Umaga – Museo civico di Umago.

81. Babić, I., Kirigin, B., & Paraman, L. (2020). *Iz prapočetaka Trogira. Zaštitna arheološka istraživanja u sklopu palače Garagnin – Fanfogna (Muzej grada Trogira) 1978. – 1980. godine*. Trogir: “Radovan” Društvo za zaštitu kulturnih dobara Trogira.

82. Paraman, L. (2009). *Helenistički Trogir*. Ph.D thesis: University of Zadar.

83. Paraman, L., Ugarković, M., Ableidinger, J., Rembart, L., Oberndorfer, D., Visković, E., Schintlmeister, L., Klostermann, P., Binder, M., & Steskal, M. (2020). Report on New Excavations in Ancient Trogir: The 2018 Croatian–Austrian Mission. *Jahreshefte Des Österreichischen Archäologischen Institutes*, *88*(2019), 387–460.

84. Babić, I. (2016). *Trogir: grad i spomenici*. Književni krug; Muzej hrvatskih arheoloških spomenika.

85. Paraman, L., & Ugarković, M. (2021). O „helenističkom“ pogrebnom nalazu s Čiova kod Trogira. *Annales Instituti Archaeologici*, *17*(1), 112–122.

86. Bilić, M. (2012). Trogir – Put Dragulina. *Hrvatski Arheološki Godišnjak* , *8*(2011), 698–700.

87. Paige, J. M. (2020). *The mother-infant dyad: reconstructing maternal nutritional status at Put Dragulina cemetery*. M.A. Thesis: Mississippi State University.

88. Osterholtz, A. J., Paraman, L., & Paige, J. M. (2022). Maternal-Infant Health in Roman Tragurium: Bioarchaeological Investigations of the Very Young. *International Congress on Roman Bioarchaeology, 20–21 October 2022 (Virtual). Abstract Book* , 7–8.

89. Paraman, L. (2017). Malo polje – Dragulin. *Hrvatski Arheološki Godišnjak*, *13*(2016), 881–884.

90. Osterholtz, A. J. (2018). Perinatal health as an indicator of maternal health factors during the Roman Civil War: preliminary analysis of the Put Dragulina Cemetery, Trogir, Croatia. *87th Annual Meeting of the American Association of Physical Anthropologists, April 11–14, 2018, Austin, Texas. Book of Abstracts*, 195–196.

91. Paraman, L. (2017). Spasilačko istraživanje segmenta rimskodobne nekropole u ulici Put Dragulina u Trogiru. *Program i Sažeci. Hrvatsko Arheološko Društvo, Muzej Grada Kaštela*, *14*, 2–6.

92. Mirković, M. (2007). *Moesia Superior: eine Provinz an der mittleren Donau*. von Zabern.

93. Mirković, M. (1968). Rimski gradovi na Dunavu u Gornjoj Meziji. *Arheološko Društvo Jugoslavije*.

94. Kazhdan, A. (1991). *Oxford Dictionary of Byzantium* (I. Djurić & A. Kazhdan, Eds.; Vol. 1). Oxford University Press.

95. Mikić, I. (2018). Roman Limes and Cities in Serbia. *Galery of the Serbian Academy of Science and Arts Publications*, *Catalogue*.

96. Mikić, Ž. (1980). O antropološkim tipovima prisutnim u antičkim nekropolama Viminacijuma. *Starinar*, *41*, 117–122.

97. Nikolić, S., Raičković-Savić, A., & Mitić, A. (2023). Keramičke posude iz Viminacijuma. *Arheološki Institut Beograd*, *in print*.

98. Korać, M., & Mikić, Ž. (2014). Anthropological collection Viminacium I The Pećine Necropolis. *Belgrade: Center for New Technology, Archeological Institute Belgrade*.

99. Spasić, D. (1990). Srednjovekovne nekropola “Kod groblja” u Starom Kostolcu. *VIMINACIUM Požarevac* , *4–5*, 157–176.

100. Jovanović, B. (2018). *Early La Tène Pećine Necropolis*. Institute of Archaeology.

101. Vulović, D., Miladinović-Radmilović, N., & Mikić, I. (2019). A case of myositis ossificans traumatica on one skeleton from Viminacium. *Starinar*, *69*, 203–214.

102. Спасић Д. (1990). Средњовековна некропола „Код гробља” у Старом Костолцу. *Viminacivum*, *4*(5), 157–175.

103. Mikić, I., & Korać, M. (2011). Viminacium – The Pećine necropolis-skeletons around Late Antique Buildings „A“ and „B“. *Arheologija i Prirodne Nauke / Archaeology and Science* , *7*, 185–199.

104. Vojvoda, M., & Mrđić, N. (2017). Coin finds from the Viminacium necropolis of Pećine and their role in funerary ritual. *Beograd: Arheološki Institut, Belgrade, Institute of Archaeology*.

105. Borić, D., & Griffiths, S. (2015). The Living and The Dead, Memory and Transition: Bayesian Modelling of Mesolithic and Neolithic Deposits from Vlasac, the Danube Gorges. *Oxford Journal of Archaeology*, *34*(4), 343–364.

106. Cook, G. T., Bonsall, C., Hedges, R. E. M., McSweeney, K., Boroneant, V., Bartosiewicz, L., & Pettitt, P. B. (2002). Problems of dating human bones from the Iron Gates. *Antiquity*, *76*(291), 77–85.

107. Redžić, S. (2014). Rimske pojasne garniture na tlu Srbije od I do IV veka. In *Универзитет у Београду*. Ph.D. Thesis: University of Belgrade.

108. Korać, M. (2018). Oil Lamps from Viminacium (Moesia Superior). Plates. *Institute of Archeology of Belgrade*, *2*.

109. Marrese, G., Tucci, P., & Raičković Savić, A. (2015). Roman pottery from Viminacium (Serbia, 2nd-3rd centuries AD): compositional characteristics, production and technological aspects. *Arheologija i Prirodne Nauke / Archaeology and Science* , *10*, 9–44.

110. Bjelajac, L. (1990). *Terra sigillata u Gornjoj Meziji: import i radionice Viminacium-Margum*. Arheoloski institut.

111. Golubović, S. (2008). *Grobovi u obliku bunara sa nekropola Viminacijuma*. Arheološki institut.

112. Raičković, A. (2007). Keramičke posude zanatskog centra iz Viminacijuma. *Centar Za Nove Tehnologije Viminacijum i Arheološki Institut*.

113. Ivány, D. (1925). Die pannonischen Lampen. *Dissertationes Pannonicae*, *2*(2), 16–17.

114. Loeschcke, S. (1919). *Lampen aus Vidonissa: Ein Beitrag zur Geschichte von Vindonissa und des antiken Beleuchtungswesens*. In Kommission bei Beer & Cie.

115. Mikić, I. (2017). An overview of the study of trepanation in the territory of Serbia. *Arheologija i Prirodne Nauke / Archaeology and Science* , *13*, 145–153.

116. Nikolić, E. (2013). Contribution to the Study of Roman Architecture in Viminacium: Construction materials and building techniques. *Archaeology and Science*, *8*, 21–48.

117. Golubović, S., Mrđić, N., & Speal, S. (2009). Killed by an Arrow – Grave 152 from Viminacium. *XVI ROMEC (The Roman Military Equipment Conference), Xantener Berichte*, *16*, 5–63.

118. Milovanović, B., & Raičković, A. (2009). Research results of the southwest Part of the site Pirivoj (Viminacium). *Arheologija i Prirodne Nauke / Archeology and Science* , *5*, 29–57.

119. Danković, I. (2012). Route of the Eastern Road of Viminacium. In L. Vagalinski & N. Sharankov (Eds.), *LIMES XXII, Proceedings of 22nd International Congress of Roman Frontier Studies* (pp. 557–562).

120. Danković, I., Milovanović, B., & Mikić, I. (2018). Zaštitna arheološka iskopavanja na lokalitetu Pirivoj (Viminacijum) 2016. godine. *Arheologija u Srbiji: Projekti Arheološkog Instituta u 2016. Godini*, 35–42.

121. Ambrose, S. H., & Krigbaum, J. (2003). Bone chemistry and bioarchaeology. *Journal of Anthropological Archaeology*, *22*(3), 193–199.

122. Ubelaker, D. H., & Grant, L. G. (1989). Human skeletal remains: Preservation or reburial? *American Journal of Physical Anthropology*, *32*(S10), 249–287.

123. Hünemörder, C. (2006). “Eagle.” In H. Canick & C. F. Salazar (Eds.), *Brill’s New Pauly*.

124. Norris, J., & Al‐Manaser, A. (2020). Epigraphica Dusaria I. Some Nabataean, Hismaic and Hismaic/Nabataean inscriptions with rock drawings from the Ḥismā desert, north‐west of Tabūk (Saudi Arabia). *Arabian Archaeology and Epigraphy*, *31*(2), 436–472.

125. Milovanović, B. D., & Danković, I. D. (2020). Anthropomorphic amulets from Viminacium. *Starinar*, *70*, 127–143.

126. Danković, I. (2020). *Inventar grobova ženske populacije kao odraz životnog stila – Studija slučaja viminacijumskih nekropola od I do IV veka*. Ph.D. Thesis: University of Belgrade.

127. Milovanović, B., & Redžić, S. (2022). Jewellery and brooches: Between functionality, aesthetic value, and status symbol. In *VIVERE IN VRBE* (Vol. 321, pp. 285–427).

128. Danković, I., & Marjanović, M. (2022). Woman, wife, mother: everyday life of female residents of Viminacium. In *VIVERE IN VRBE* (Vol. 274, pp. 203–282).

129. Spasić-Đurić, D. (2016). A note of new archaeological explorations in byzantine Braničevo. In V. Bikić (Ed.), *Byzantine Heritage and Serbian art: Vol. I* (pp. 109–115). Serbian Academy of Arts and Sciences.

130. Stephenson, P. (2008). Balkan Borderlands (1018-1204). In J. Shepard (Ed.), *The Cambridge history of the Byzantine Empire c. 500-1492* (pp. 91–664). Cambridge University Press.

131. Поповић, М., & Иванишевић, В. (1988). Град Браничево у средњем веку. *Старинар. XXXIX*.

132. Korać, M., & Golubović, S. (2009). Viminacium - Više Grobalja II. *Institute of Archaeology Belgrade*.

133. Зотовић, Љ., & Јордовић, Ч. (1990). Некропола Више Гробаља. *Viminacivm* , *I*.

134. Ivanišević, V., Kazanski, M., & Mastykova, A. (2006). *Les nécropoles de Viminacium à l’époque des Grandes Migrations*. Association des amis du Centre d’histoire et civilisation de Byzance.

135. Redžić, S. (2007). Nalazi rimskih fibula na nekropolama Viminacijuma. *Beograd: Centar Za Nove Tehologije Viminacium: Arheološki Institut.*

136. Vojvoda, M., & Nemanja, M. (2015). *Coin finds from the Viminacium Necropolis of Više Grobalja*. Institute of Archaeology, Belgrade.

137. Bialeková, D. (1984). Zisťovací výskum a povrchový prieskum vo Výčapoch-Opatovciach. . *Archeologické Výskumy a Nálezy Na Slovensku v Roku 1983*, 41–45.

138. Budinský-Krička, V. (1947). Slovanské popolnicové pole z doby predhradištnej vo Výčapoch-Opatovciach, okr. Nitra. *Historica Slovaca* , *5*, 135–144.

139. Tocik, A. (1979). Vycapy Opatovce und weitere altbronzezeitliche Gräberfelder in der Südwestslowakei. *Materialia Archaeologica Slovaca Nitra-Hrad*, *1*, 1–366.

140. Fadić, I. (2008). Zadar – Relja (Vrt Relja). . *Hrvatski Arheološki Godišnjak*, *3*(2007), 350–352.

141. Fadić, I. (2008). Zadar-Relja (Trgovački centar). *Hrvatski Arheološki Godišnjak*, *3*(2006), 347–349.

142. Alihodžić, T. (2010). Zadar – Polačišće i Ulica Petra Svačića. *Hrvatski Arheološki Godišnjak*, *5*(2009), 508–509.

143. Alihodžić, T. (2011). Zadar – Relja (parkiralište). *Hrvatski Arheološki Godišnjak*, *6*(2010), 540–543.

144. Plohl, M. (2018). Burial Typology at the" Vrt Relja" Necropolis in Zadar. *Miscellanea Hadriatica et Mediterranea*, *5*, 65–97.

145. Glušćević, S. (2005). Zadarske nekropole od 1. do 4. stoljeća. *Organizacija Groblja, Pogrebni Obredi, Podrijetlo, Kultura, Status i Standard Pokojnika. Neobjavljena Doktorska Disertacija. Zadar: Sveučilište u Zadru*.

146. Cruciani, F., La Fratta, R., Trombetta, B., Santolamazza, P., Sellitto, D., Colomb, E. B., Dugoujon, J. M., Crivellaro, F., Benincasa, T., Pascone, R., Moral, P., Watson, E., Melegh, B., Barbujani, G., Fuselli, S., Vona, G., Zagradisnik, B., Assum, G., Brdicka, R., … Scozzari, R. (2007). Tracing past human male movements in northern/eastern Africa and western Eurasia: New clues from Y-chromosomal haplogroups E-M78 and J-M12. *Molecular Biology and Evolution*, *24*(6), 1300–1311. https://doi.org/10.1093/molbev/msm049

147. Lazaridis, I., Alpaslan-Roodenberg, S., Acar, A., Açıkkol, A., Agelarakis, A., Aghikyan, L., Akyüz, U., Andreeva, D., Andrijašević, G., & Antonović, D. (2022). The genetic history of the Southern Arc: A bridge between West Asia and Europe. *Science*, *377*(6609), eabm4247.

148. Patterson, N., Isakov, M., Booth, T., Büster, L., Fischer, C.-E., Olalde, I., Ringbauer, H., Akbari, A., Cheronet, O., & Bleasdale, M. (2022). Large-scale migration into Britain during the Middle to Late Bronze Age. *Nature*, *601*(7894), 588–594.

149. Posth, C., Zaro, V., Spyrou, M. A., Vai, S., Gnecchi-Ruscone, G. A., Modi, A., Peltzer, A., Mötsch, A., Nägele, K., & Vågene, Å. J. (2021). The origin and legacy of the Etruscans through a 2000-year archeogenomic time transect. *Science Advances*, *7*(39), eabi7673.

150. Antonio, M. L., Gao, Z., Moots, H. M., Lucci, M., Candilio, F., Sawyer, S., Oberreiter, V., Calderon, D., Devitofranceschi, K., Aikens, R. C., Aneli, S., Bartoli, F., Bedini, A., & Cheronet, O. (2019). Ancient Rome: A genetic crossroads of Europe and the Mediterranean. *Science*, *714*(November), 708–714.

151. Aneli, S., Saupe, T., Montinaro, F., Solnik, A., Molinaro, L., Scaggion, C., Carrara, N., Raveane, A., Kivisild, T., & Metspalu, M. (2022). The genetic origin of Daunians and the Pan-Mediterranean southern Italian Iron Age context. *Molecular Biology and Evolution*, *39*(2), msac014.

152. Žegarac, A., Winkelbach, L., Blöcher, J., Diekmann, Y., Krečković Gavrilović, M., Porčić, M., Stojković, B., Milašinović, L., Schreiber, M., & Wegmann, D. (2021). Ancient genomes provide insights into family structure and the heredity of social status in the early Bronze Age of southeastern Europe. *Scientific Reports*, *11*(1), 1–11.

153. Harney, É., May, H., Shalem, D., Rohland, N., Mallick, S., Lazaridis, I., Sarig, R., Stewardson, K., Nordenfelt, S., & Patterson, N. (2018). Ancient DNA from Chalcolithic Israel reveals the role of population mixture in cultural transformation. *Nature Communications*, *9*(1), 3336.

154. Agranat-Tamir, L., Waldman, S., Martin, M. A. S., Gokhman, D., Mishol, N., Eshel, T., Cheronet, O., Rohland, N., Mallick, S., & Adamski, N. (2020). The genomic history of the Bronze Age southern Levant. *Cell*, *181*(5), 1146–1157.

155. Skourtanioti, E., Erdal, Y. S., Frangipane, M., Balossi Restelli, F., Yener, K. A., Pinnock, F., Matthiae, P., Özbal, R., Schoop, U. D., Guliyev, F., Akhundov, T., Lyonnet, B., Hammer, E. L., Nugent, S. E., Burri, M., Neumann, G. U., Penske, S., Ingman, T., Akar, M., … Krause, J. (2020). Genomic History of Neolithic to Bronze Age Anatolia, Northern Levant, and Southern Caucasus. *Cell*, *181*(5), 1158-1175.e28. https://doi.org/10.1016/j.cell.2020.04.044

156. Novak, M., Olalde, I., Ringbauer, H., Rohland, N., Ahern, J., Balen, J., Janković, I., Potrebica, H., Pinhasi, R., & Reich, D. (2021). Genome-wide analysis of nearly all the victims of a 6200 year old massacre. *Plos One*, *16*(3), e0247332.

157. Narasimhan, V. M., Patterson, N., Moorjani, P., Rohland, N., Bernardos, R., Mallick, S., Lazaridis, I., Nakatsuka, N., Olalde, I., & Lipson, M. (2019). The formation of human populations in South and Central Asia. *Science*, *365*(6457), eaat7487.

158. Margaryan, A., Lawson, D. J., Sikora, M., Racimo, F., Rasmussen, S., Moltke, I., Cassidy, L. M., Jørsboe, E., Ingason, A., & Pedersen, M. W. (2020). Population genomics of the Viking world. *Nature*, *585*(7825), 390–396.

159. Allentoft, M. E., Sikora, M., Sjögren, K. G., Rasmussen, S., Rasmussen, M., Stenderup, J., Damgaard, P. B., Schroeder, H., Ahlström, T., Vinner, L., Malaspinas, A. S., Margaryan, A., Higham, T., Chivall, D., Lynnerup, N., Harvig, L., Baron, J., Casa, P. Della, Dąbrowski, P., … Willerslev, E. (2015). Population genomics of Bronze Age Eurasia. *Nature*, *522*(7555), 167–172. https://doi.org/10.1038/nature14507

160. Underhill, P. A., Poznik, G. D., Rootsi, S., Järve, M., Lin, A. A., Wang, J., Passarelli, B., Kanbar, J., Myres, N. M., & King, R. J. (2015). The phylogenetic and geographic structure of Y-chromosome haplogroup R1a. *European Journal of Human Genetics*, *23*(1), 124–131.

161. Pericic, M., Lauc, L. B., Klaric, I. M., Rootsi, S., Janićijević, B., Rudan, I., Terzić, R., Čolak, I., Kvesić, A., & Popović, D. (2005). High-resolution phylogenetic analysis of southeastern Europe traces major episodes of paternal gene flow among Slavic populations. *Molecular Biology and Evolution*, *22*(10), 1964–1975.

162. Kuhn, J. M. M., Jakobsson, M., & Günther, T. (2017). Estimating Genetic Kin Relationships in Prehistoric Populations. *PloS One*, *13*(4), e0195491. https://doi.org/10.1101/100297

163. Rohland, N., Mallick, S., Mah, M., Maier, R. M., Patterson, N. J., & Reich, D. (2022). Three assays for in-solution enrichment of ancient human DNA at more than a million SNPs. *Genome Research*, *32*(11–12), 2068–2078.

164. Lazaridis, I., Nadel, D., Rollefson, G., Merrett, D. C., Rohland, N., Mallick, S., Fernandes, D., Novak, M., Gamarra, B., Sirak, K., Connell, S., Stewardson, K., Harney, E., Fu, Q., Gonzalez-Fortes, G., Jones, E. R., Roodenberg, S. A., Lengyel, G., Bocquentin, F., … Reich, D. (2016). Genomic insights into the origin of farming in the ancient Near East. *Nature*, *536*(7617), 419–424. https://doi.org/10.1038/nature19310

165. Cardoso, S., Valverde, L., Alfonso-Sánchez, M. A., Palencia-Madrid, L., Elcoroaristizabal, X., Algorta, J., Catarino, S., Arteta, D., Herrera, R. J., & Zarrabeitia, M. T. (2013). The expanded mtDNA phylogeny of the Franco-Cantabrian region upholds the pre-neolithic genetic substrate of Basques. *PloS One*, *8*(7), e67835.

166. Olalde, I., Mallick, S., Patterson, N., Rohland, N., Villalba-Mouco, V., Silva, M., Dulias, K., Edwards, C. J., Gandini, F., Pala, M., Soares, P., Ferrando-Bernal, M., Adamski, N., Broomandkhoshbacht, N., Cheronet, O., Culleton, B. J., Fernandes, D., Lawson, A. M., Mah, M., … Reich, D. (2019). The genomic history of the Iberian Peninsula over the past 8000 years. *Science*, *363*(6432), 1230–1234. https://doi.org/10.1126/science.aav4040

167. Gelabert, P., Sandoval-Velasco, M., Olalde, I., Fregel, R., Rieux, A., Escosa, R., Aranda, C., Paaijmans, K., Mueller, I., & Gilbert, M. T. P. (2016). Mitochondrial DNA from the eradicated European Plasmodium vivax and P. falciparum from 70-year-old slides from the Ebro Delta in Spain. *Proceedings of the National Academy of Sciences*, *113*(41), 11495–11500.

168. Matisoo-Smith, E., Gosling, A. L., Platt, D., Kardailsky, O., Prost, S., Cameron-Christie, S., Collins, C. J., Boocock, J., Kurumilian, Y., Guirguis, M., Pla Orquín, R., Khalil, W., Genz, H., Abou Diwan, G., Nassar, J., & Zalloua, P. (2018). Ancient mitogenomes of Phoenicians from Sardinia and Lebanon: A story of settlement, integration, and female mobility. *PLoS ONE*, *13*(1), 1–19. https://doi.org/10.1371/journal.pone.0190169

169. Heather, P. (2010). *Empires and barbarians: migration, development and the birth of Europe*. Pan Macmillan.

170. Patterson, N., Moorjani, P., Luo, Y., Mallick, S., Rohland, N., Zhan, Y., Genschoreck, T., Webster, T., & Reich, D. (2012). Ancient admixture in human history. *Genetics*, *192*(3), 1065–1093.

171. Harney, É., Patterson, N., Reich, D., & Wakeley, J. (2021). Assessing the performance of qpAdm: A statistical tool for studying population admixture. *Genetics*, *217*(4). https://doi.org/10.1093/GENETICS/IYAA045

172. Gnecchi-Ruscone, G. A., Khussainova, E., Kahbatkyzy, N., Musralina, L., Spyrou, M. A., Bianco, R. A., Radzeviciute, R., Gomes Martins, N. F., Freund, C., Iksan, O., Garshin, A., Zhaniyazov, Z., Bekmanov, B., Kitov, E., Samashev, Z., Beisenov, A., Berezina, N., Berezin, Y., Bíró, A. Z., … Krause, J. (2021). Ancient genomic time transect from the Central Asian Steppe unravels the history of the Scythians. *Science Advances*, *7*(13). https://doi.org/10.1126/sciadv.abe4414
