# Supplementary material for: A Genetic History of the Balkans from Roman Frontier to Slavic Migrations
Source: Cell. Author manuscript; Available in PMC 2023 Dec 27. (PMC10752003; doi:10.1016/j.cell.2023.10.018)
Supplement: Data S1 — Supplementary information: archeological and historical context of the archeological sites reported in this study, additional statistical analysis and discussion. Related to STAR Methods. [file NIHMS1944038-supplement-Data_S1.docx]

Supplemental information

**A Genetic History of the Balkans from Roman Frontier to Slavic Migrations**

Iñigo Olalde*, Pablo Carrión, Ilija Mikić, Nadin Rohland, Swapan Mallick, Iosif Lazaridis, Matthew Mah, Miomir Korać, Snežana Golubović, Sofija Petković, Nataša Miladinović-Radmilović, Dragana Vulović, Timka Alihodžić, Abigail Ash, Miriam Baeta, Juraj Bartík, Željka Bedić, Maja Bilić, Clive Bonsall, Maja Bunčić, Domagoj Bužanić, Mario Carić, Lea Čataj, Mirna Cvetko, Ivan Drnić, Anita Dugonjić, Ana Đukić, Ksenija Đukić, Zdeněk Farkaš, Pavol Jelínek, Marija Jovanovic, Iva Kaić, Hrvoje Kalafatić, Marijana Krmpotić, Siniša Krznar, Tino Leleković, Marian M. de Pancorbo, Vinka Matijević, Branka Milošević Zakić, Anna J. Osterholtz, Julianne M. Paige, Dinko Tresić Pavičić, Zrinka Premužić, Petra Rajić Šikanjić, Anita Rapan Papeša, Lujana Paraman, Mirjana Sanader, Ivana Radovanović, Mirjana Roksandic, Alena Šefčáková, Sofia Stefanović, Maria Teschler-Nicola, Domagoj Tončinić, Brina Zagorc, Kim Callan, Francesca Candilio, Olivia Cheronet, Daniel Fernandes, Aisling Kearns, Ann Marie Lawson, Kirsten Mandl, Anna Wagner, Fatma Zalzala, Anna Zettl, Željko Tomanović, Dušan Keckarević, Mario Novak, Kyle Harper, Michael McCormick, Ron Pinhasi, Miodrag Grbić, Carles Lalueza-Fox*, David Reich*

**Other Supplementary Materials for this manuscript include the following:**

Data S2: Data Tables, related to STAR Methods.

Data S1: Supplementary information: archeological and historical context of the archeological sites reported in this study, additional statistical analysis and discussion. Related to STAR Methods.

1. Archaeological Overview: contexts of newly reported individuals (ed. Kyle Harper and Michael McCormick)
2. Y-chromosome patterns
3. Analysis of kinship relationships within archaeological sites
4. *qpAdm* admixture modelling of ancient individuals
5. *qpAdm* admixture modelling of present-day Balkan and Aegean populations
6. **Archaeological Overview: Contexts of newly reported individuals** (ed. Kyle Harper and Michael McCormick)

Edited by Michael McCormick and Kyle Harper, with assistance from Pablo Carrión and Iñigo Olalde; contributions by Timka Alihodžić, Juraj Bartík, Željka Bedić, Maja Bilić, Clive Bonsall, Maja Bunčić, Domagoj Bužanić, Lea Čataj, Mirna Cvetko, Ivan Drnić, Anita Dugonjić, Ana Đukić, Zdeněk Farkaš, Pavol Jelínek, Marija Jovanović, Iva Kaić, Hrvoje Kalafatić, Marijana Krmpotić, Siniša Krznar, Tino Leleković, Vinka Matijević, Ilija Mikić, Nataša Miladinović-Radmilović, Branka Milošević Zakić, Anna J. Osterholtz, Julianne M. Paige, Lujana Paraman, Sofija Petković, Zrinka Premužić, Petra Rajić Šikanjić, Anita Rapan Papeša, Mirjana Sanader, Alena Šefčáková, Maria Teschler-Nicola, Domagoj Tončinić, Dinko Tresić Pavičić, Dragana Vulović.

In this section, we provide the essential information about the archaeological context of the newly reported ancient individuals in alphabetical order of the site or, under the site, the sub-site name. Geographic coordinates for each site are given in Data S2, Table 1. All calibrated radiocarbon dates are given at two standard deviations (for details, see Data S2, Table 9). In cases where genomic analysis was possible and unless otherwise indicated, individuals have been sexed based on ancient DNA data; when genetic information was lacking, the basis for sexing is signaled, e.g., physical anthropology. Unlike traditional methods of genetic sexing based on the study of one or a few genetic markers, the determination of genetic sex with genome-wide data in ancient remains (see Material and Methods) has proven a very reliable method, even in cases of low-quality DNA data^[1]^.

The excavating and/or regionally expert archaeologists have supplied the basic bibliography and information, some of which is unpublished, for each site. Their materials have been assembled and compiled by Pablo Carrion and Iñigo Olalde, and revised, vetted and standardized to the extent possible among so many differing scholarly traditions, and edited by Kyle Harper and Michael McCormick with the generous advice of our colleagues in the region, with the aim of making this conspectus of published and unpublished archaeological and historical information as useful as possible for those who wish to assess the full implications of the present article and who would use the new findings for further archaeogenetic, archaeological and historical research.

**1.1 Blatné, Senec district (Slovakia)**

Contact persons**:** Alena Šefčáková, Juraj Bartík, Zdeněk Farkaš, Pavol Jelínek

**Geographic Information**: The site is located in the village of Blatné, in the Senec district in the Bratislava region (western Slovakia), north of the Danube, 25 km northeast of modern Bratislava and roughly 5 km north of the town of Senec.

**Excavation History:** Rescue excavations in Blatné (Senec district, Trnava Region) were carried out on the edge of a loess terrace, which protrudes above the floodplain of Danube tributaries inundated in the past up to 9 to 10 m. In 2000 and 2002, an area of 895 m^2^ was investigated and about 47 graves were uncovered at an Early Bronze Age cemetery.

**Summary of sampled materials:** During excavation, four graves were discovered with finds dating from the Middle Ages. With the help of radiocarbon dating, three additional ones (graves [Blatné] 2, 34 and 39) were assigned to the Medieval Period. The cemetery has not yet been published; only partial information is presently available ^[2]^.

We sampled one Early Medieval grave:

- **Genetic Identifier:** I12991. **Grave Identifier:** Blatné 39 (Inv. No. 8504). **Grave type:** burial pit, NW-SE orientation. **Skeletal information:** female, adolescent, 15-16 years old. **Grave goods:** none. **Dating**: radiocarbon date 709-880 cal CE (1220±15 BP, PSUAMS-9770). **Additional information**: individual found during archaeological research in 2002. The damaged gracile skeleton was lying in a crouched position on the right side with her head on the SE. *Cribra orbitalia* are visible on the roof of her eye sockets.

**Ancestry summary:** Eastern European-related ancestry.

**1.2 Brekinjova Kosa, Bojna, Glina (Croatia)**

Contact person(s): Marijana Krmpotić

**Geographic Information**: The archaeological site of Brekinjova Kosa is located in the region of Banovina, approximately 67 km south of Zagreb and approximately 15 km south of Glina in Sisak-Moslavina County. This is a mountainous region on the forest-covered western slopes of Trgovska gora (“mountain”), and Brekinjova Kosa hill is situated in a narrow ravine 80-100 m deep, through which the Sivac and Bojna streams run.

**Excavation History**: In a forested mountain region about 40 km SW of Sisak/Siscia, the site lies about 15 km from the Roman roads connecting Siscia and southwestern Pannonia to the Adriatic, in a region situated on the border between Roman provinces Pannonia and Dalmatia, which came into the sphere of interest of the Carolingian Empire during Charlemagne’s war against the Avar Khaganate. An Early Iron Age hillfort was documented by a field survey conducted in 2010. During rescue excavations conducted in 2015 due to the potential destruction of the site by activities at the Bojna quarry, the central part of the upper plateau was investigated (a surface area of 2950 m²). In addition to prehistoric contexts, eleven Early Medieval graves and a waste-pit were recorded, together with numerous small finds from this period (fragments of pottery vessels datable from the middle of the 7^th^ to the 9^th^ century CE). The foundations of a heavily eroded stone structure were recorded to the north of the central part of the hillfort’s upper plateau. The structure is oriented east-west; the eastern end has eroded away, leaving unclear whether it may have been an early medieval church. Within and around the structure, eleven early medieval inhumation burials were investigated. An exceptional, “princely” burial with an antechamber was partially carved into the bedrock in alignment with the southern wall of the structure. The individual was buried with splendid gilded spurs, a gold setting of a rock crystal pendant, a Byzantine gold coin (solidus) issued at Syracuse, 760-775 CE, and had worn a garment woven with gold thread. The skeleton was very poorly preserved and the seventh-century radiocarbon date of a tarsus bone must be disregarded in light of the coin, grave goods, and the 8^th^-9^th^ century dates of wood from the burial ^[3]^. Some kind of link connected this individual with the contemporary elite burials featuring identical coins some 130 and 180 km to the south, at Biskupija and Trilj (Croatia), respectively ^[4]^.

Further systematic archaeological excavations, which have been conducted since 2016, revealed the remains of an early medieval fortified settlement with more than 50 contemporaneous graves. Some of the graves were part of a cemetery organized in rows, although most of them were dug inside the settlement. Graves and other features were dated on the basis of the radiocarbon analysis and comparable finds from the second half of the 7^th^ century to the entire 9^th^ century ^[3]^.

**Summary of sampled materials:** Sampled materials belonged to the Early Medieval necropolis, 2015 excavation campaign.

We sampled one grave with two individuals from this necropolis:

- **Genetic Identifier:** I26748 and I26749. **Grave Identifier:** Grave 5 A and B. **Grave type:** Deceased in a wooden coffin, W-E orientation (heads at the western end). **Skeletal information:** A: male, young adult; B: male, around 50 years old. **Grave goods:** a small iron nail and five rhomboid iron strips which must have been embedded in wood probably indicate a wooden burial chamber or coffin. **Dating**: A: radiocarbon date 773-890 cal CE (1190±20 BP, PSUAMS-12036; and B: radiocarbon date 774-885 cal CE (1200±20 BP, PSUAMS-12037). **Additional information**: this grave is located on the northeastern slope in the upper row next to grave 2. The upper part of the grave-pit has an oval rather than rectangular layout. Halfway through the fill, at a depth of around 1 m, cranial fragments were recorded on the western side of the grave along with a slightly different fill (darker in color and looser in consistency) probably representing the position of a disintegrated coffin or some other wooden structure in which the two deceased individuals had been laid in. The discovered skeletal remains were very badly preserved. The recovered cranial fragments belonging to two adult individuals further displayed: A: an antemortem healed puncture wound on the right side of the frontal bone; B: one carious lesion on maxillary M3.

**Ancestry summary**: A: Anatolia Roman+Eastern Europe; B: Balkans Iron Age-related.

**1.3 Čepinski Martinci-Dubrava (Croatia)**

Contact persons: Siniša Krznar, Hrvoje Kalafatić

**Geographic Information**: Čepinski Martinci-Dubrava site is located south-west of Čepin, south of the Čepin Čepinski Martinci road, near Čepinski Martinci village.

**Brief settlement history**: The archaeological site covers an area of 220,000 m^2^ and includes Chalcolithic (Lsinja, Retz-Gajary and Baden cultures) settlements, a Bronze Age settlement and cemetery, as well as settlements from Late Antiquity and the Early Middle Ages.

**Excavation History**: The site was found and investigated during rescue archaeological research on the route of the highway 5C running through Croatia from Beli Manastir to Svilaj, on the Osijek–Đakovo route section. The Institute of Archaeology conducted research in 2007 and 2008. In the area of the connection road, at the eastern edge of the Lasinja culture settlement, several small pits were discovered which belong to the southern part of the early medieval settlement, excavated at the neighbouring Čepin-AN COKP site. The remains of an early medieval settlement were found north of the Bronze Age settlement, which is connected and chronologically simultaneous with the settlement unearthed on the connection road and dated from the 8^th^ to 11^th^ centuries ^[5]^ CE.

**Summary of sampled materials:** The sampled individual was discovered in the northern part, in the surroundings of the Early Medieval settlement features (dwellings, pits, wells, etc.) ^[6]^.

Sampled grave:

- **Genetic Identifier:** I35081. **Grave Identifier:** G23, U-1786. **Grave type:** no visible burial container, W-E orientation. **Skeletal information:** female (genetically assigned), 30-50 years old. **Grave goods:** none. **Dating**: radiocarbon date (charcoal found close to the deceased) 657-873 cal CE (1280±40 BP; Beta-278778) ^[6]^ **Ancestry summary:** Eastern European-related. **Additional information:** buried in the eastern part of an elongated pit of irregular shape, placed along its northern edge, and stretching west-east. Although the skeleton was quite damaged, it was obvious that the deceased was laid on her back with her legs bent over at the knees ^[7]^.

**1.4 Gardun (Croatia)**

Contact person(s): Mirjana Sanader, Domagoj Tončinić, Iva Kaić, Vinka Matijević, Mirna Cvetko, Domagoj Bužanić

**Geographic Information:** on the southern approaches to the modern village of Gardun atop the hill of the same name, the site overlooks the Roman road connecting the Danube through the Dinaric Alps to the provincial capital of Salona, some 20 km distant, on the Adriatic.

**Brief settlement history**: The legionary fortress of Tilurium was situated in the territory of the present-day village of Gardun, on a dominating and strategic position on the right bank of the Cetina river, controlling the surrounding fields and plateaus, as well as the crossing over the Cetina. The most prominent finds are tombstones and other inscriptions bearing testimony to the presence of various Roman military units at *Tilurium* ^[8]^. The most represented unit is *Legio VII*, or *Legio VII Claudia pia fidelis*. The oldest reliably dated stone monuments of the *Legio VII* in Dalmatia are the so-called *Tabulae Dolabellae*, dating to the year 16/17 CE. Some tombstones of the *Legio VII* from *Tilurium* have been dated palaeographically to the 1^st^ century CE ^[9]^. Most authors agree that the latest plausible date for the arrival of the VII legion in *Tilurium* was during the Dalmatian-Pannonian revolt of 6-9 CE, or immediately afterward ^[10]^. Some finds of Roman military equipment from Tilurium may date even to the 4^th^ decade of the 1^st^ century BCE ^[11]^. After the departure of *Legio VII Claudia pia fidelis* to Viminacium in the province of Moesia ca. 50 CE, auxiliary units and perhaps divisions of other legionary units were stationed in *Tilurium*. The last auxiliary unit probably left *Tilurium* soon after 245 CE^[12]^. After the departure of the last military unit from *Tilurium*, archaeological evidence indicates drastic changes regarding the organization of space. Graves and tombs were established within the former fortress and, in some cases, individual structures were transformed and/or completely destroyed. The successor occupation remains to be characterized archaeologically, but one of the deceased individuals has been radiocarbon dated to 432-598 cal CE, and a metal buckle is of similar date.

**Excavation History:** A systematic archaeological investigation at Tilurium, conducted since 1997 by the Department of Archaeology, Faculty of Philosophy, University of Zagreb with Mirjana Sanader as the Project Director, is the first such research done at this site. It was organised as a part of “Roman Military Camps in Croatia”, a scientific research project (130-0000000-0777) backed by the Ministry of Science, Education and Sport of the Republic of Croatia since 1997, with the support of the Ministry of Culture of the Republic of Croatia, Split-Dalmatian County, the town of Trilj, and the residents of Gardun and Vojnić.

**Summary of sampled materials:** The osteological material analyzed for this paper was found inside the graves that were archaeologically documented within excavation area A, located in the southeastern part of the fortress. Excavation area A consists of military barrack blocks (*centuriae*), which were partially dismantled and transformed in Late Antiquity. This is evidenced by the stratigraphy, specifically of the walking surface between two *centuriae* which was intentionally completely covered with a layer of stones, mortar and architectural elements originating from the walls of the *centuriae* ^[13]^. The spatial transformation is further confirmed by the tomb SU 165 that was built above the stone rubble fill layer and along the southern face of a *centuria’s* wall which was reused as the northern edge of the tomb. The tomb is dated by a coin of Emperor Valens (364-378 CE) and a belt buckle attributed ^[13]^ to a type used among the Eastern Goths in the second half of the 5^th^ or the 6^th^ century. The graves are oriented east-west; the skeletons were disarticulated and individuals’ orientations within the tombs were not recorded.

**Sampled Graves:**

- **Genetic Identifier:** I26894. **Grave Identifier:** SU 19 / Grave 1A from 1999. **Grave type:** deceased in grave with stone block construction, E-W orientation (see Summary). **Skeletal information:** female, 1.5–3 years old. **Grave goods:** none ^[14]^. **Dating**: stratigraphy: 360-600 CE. **Additional information**: the grave was found in the excavation area A, in the space between the abandoned military sleeping quarters (*centuriae*) and the ramparts, suggesting that the area was no longer used as a fortress at the time of the burial. The internal dimensions of the grave were 60 x 40 cm. Osteological analysis showed that two children were buried there, apparently within a short space of time.

**Ancestry summary**: Balkans Iron Age+Anatolia Roman.

- **Genetic Identifier:** I26897. **Grave Identifier:** SU 176/177, 490; SU 176/177, C (two samples from the same individual). **Grave type:** deceased in grave with stone block construction, E-W orientation ^[13]^ (see Summary). **Skeletal information:** male**,** 0-1.5 years old ^[15]^. **Grave goods:** none. **Dating**: radiocarbon: 400-600 CE based on the radiocarbon date for individual SU 176/177,522 (I26899) from the same grave (see next entry). **Additional information:** Grave SU 176/177 was found in excavation area A next to the remains of an unidentified building which was partly built over the wall of the military sleeping quarters (*centuria*). One large stone block was used as the eastern wall of the grave, but on the north, south, and west sides, various irregularly arranged stones were found. A grave covering was not found. The bottom of the grave was a compact layer of small stones and earth. The internal dimensions of the tomb were 60 x 40 cm. All bones were disarticulated. Osteological analysis showed that three children aged 0-1.5 years were buried there.

**Ancestry summary**: Balkans Iron Age+Anatolia Roman.

- **Genetic Identifier:** I26899. **Grave Identifier:** SU 176/177,522. **Grave type:** deceased in grave with stone block construction, E-W orientation (see Summary). **Skeletal information:** male**,** 0-1.5 years old ^[15]^. **Grave goods:** none. **Dating**: radiocarbon: 432-598 cal CE (Beta-584485). **Additional information**: same grave as I26897 and I26896.

**Ancestry summary:** excluded from analysis dataset based on low SNP count.

- **Genetic Identifier:** I26896. **Grave Identifier:** SU 176/177,244. **Grave type:** deceased in grave with stone block construction, E-W orientation (see Summary). **Skeletal information:** sex unknown**,** 0-1.5 years old ^[15]^. **Grave goods:** none. **Dating**: radiocarbon: 400-600 CE based on the radiocarbon date for individual SU 176/177,522 (I26899) from the same grave. **Additional information**: same grave as I26897 and I26899.

**Ancestry summary:** excluded from analysis dataset based on low SNP count.

- **Genetic Identifier:** I26895. **Grave Identifier:** SU 180/181, 393 from 2006. **Grave type:** deceased in grave with stone block construction, E-W orientation ^[13]^ (see Summary). **Skeletal information:** female, 1–3 years old ^[15]^. **Grave goods:** 7 beads (dating from 1^st^ to the 7^th^ century CE), a fragment of glass, three pieces of iron, pottery fragments ^[16]^. **Dating**: 360-600 CE based on the stratigraphic context. Tomb excavated next to and belonging to the same layer as unsampled grave SU165, dated by a coin of Valens (364-378 CE) and a belt buckle attributed to the Eastern Goths and dated to the second half of the 5^th^ or the 6^th^ c. CE. **Additional information**: the grave was built along the wall of the military sleeping quarters (*centuriae*). The grave covering also consisted of stone slabs, and its bottom was a layer of compact earth and small stones. The internal dimensions of the tomb were 65 x 25 cm. The bones of the deceased were disturbed and concentrated on the east side of the grave.

**Ancestry summary**: Balkans Iron Age+Anatolia Roman.

**1.5 Gomolava, Hrtkovci, Vojvodina (Serbia)**

Contact person: Marija Jovanović

**Geographic Information**: The site of Gomolava is located on the left bank of the Sava river, next to the village of Hrtkovci (Serbia).

**Excavation History**: In the period from 1973 to 1977, a Neolithic Vinča culture necropolis was discovered with 27 inhumed deceased and six dislocated skeletons ^[17]^. In the northeastern part of the excavation block, 22 graves were discovered along with several dislocated skeletons: graves 2/1975, 3/1975, 3/1976, 4/1976, 5/1976, 56/1976, 7/1976, 8/1976, 9/1976, 10/1976, 11/1976, 12/1976, 13/1976, 14/1976, 15/1976, 18/1976, 19/1976, 20/1976, 21/1976, 22/1976, 23/1976 and 24/1977. Grave 25/1977 was found within a section control profile, between blocks I and II. In block VI, trench I, two inhumations were found: grave 16/1976 and 17/1976 and several dislocated bones. Grave 1/1973 was discovered in trench A, along the southern profile of block I, and grave 2/1973 was found in the northeastern part of block IV. Aside from these, there is also a child's grave from block III, q. 82/XIX. Inside graves 15/1976 and 23/1976, dislocated bones from multiple individuals were found. All skeletons belonged to males except for grave 17/1976 and grave 23/1976. Based on the grave findings and radiocarbon dating ^[18]^, the necropolis belongs to the last phase of the Vinča culture (Vinča D2 phase), with the exception of the human skull at Grave 1, whose radiocarbon date is Early Medieval.

**Summary of sampled materials:** Genome-wide data from graves 3, 8 and 10 were reported in Mathieson et al 2018 ^[19]^. In this study, we report data from grave 1, discovered in Block I, q. 89,90/XIV, at a relative depth of 5.71 metres, The archaeological context and radiocarbon date strongly indicates that the sampled bone is intrusive into a Vinča-period grave:

- **Genetic Identifier:** I1116. **Grave Identifier:** NG12/Grave 1/1973; mus. Inv. 6330. **Grave type:** burial in pit, E-W orientation. **Skeletal information:** male, 32-40 years. **Grave goods:** two biconical bowls were found at the level of the forehead. They clearly belong to the Vinča culture and are almost identical to other vessels found at Gomolava. **Dating:** direct radiocarbon on the petrous analyzed with aDNA: 775-994 cal CE (1125±26 BP, OxA-X-2648-37). **Additional information**: the skeleton was damaged; the head was in the east with the face turned to the south. The body was placed in a semi-crouching position. The legs were slightly bent at the knee so that the femurs and shins formed an angle of about 90 degrees.

**Ancestry summary**: Anatolia Roman+Eastern Europe.

**1.6 Gornji Kosinj – St. Ana (Croatia)**

Contact person: Lea Čataj

**Geographic Information**: The chapel of St. Ana is situated in the south-east part of Gornji Kosinj between the stream Bakovac and the Lika River. The current building, rectangular in shape with rectangular apse, was built in the early 20^th^ century on top of the older, presumably medieval chapel. According to some sources it was rebuilt in 1733 as a branch of the parish church ^[20]^.

**Brief settlement history**: The village of Gornji Kosinj is a part of Perušić municipality in the mountainous Lika-Senj County in Croatia. During the Medieval period it was a part of the so-called Bočać and later Bužani district up until the 16^th^ century when it was conquered by the Ottomans. By the late 17^th^ century the region was under Habsburg rule ^[20–22]^.

**Excavation History**: Small test excavations were carried out in 1988 by the Faculty of Humanities and the Regional Institute for Cultural Heritage Conservation from Zagreb when four small trenches were excavated in a wider area around the chapel. Only one burial was found, partially intersected by the eastern wall of the apse. The skeleton was oriented northwest-southeast, following the orientation of the chapel. It was laid on its back with arms extended along the body ^[21]^.

**Summary of sampled materials**: In 2020, further test excavations were conducted by the Croatian Conservation Institute from Zagreb. Five trenches covering 70 m^2^ were investigated: three of them around the church and two by the enclosure wall. Twenty-four graves were found in front of the chapel and along its southern walls. According to the stratigraphy of the site and grave finds, most of the individuals were buried in the period between the 10^th^ and the 14^th^ century CE: women in front of the chapel and men by the southern nave and the southern wall of the apse. Two time periods of the burials could be defined: one belonging to the 10^th^ and 11^th^ centuries (graves 10, 23 and 25), the other one to the period of the 12^th^ to 14^th^ centuries (graves 1 and 17). Graves following the orientation of the current chapel suggest the existence of an earlier church/chapel, which was presumably built in the 10^th^/11^th^ century and which had the same orientation as the present-day chapel. The sole burial outside the 10^th^- to 14^th^-century time frame is grave 3, located in front of the chapel and dated after the end of the 17^th^ century.

Sampled Graves:

- **Genetic Identifier:** I35012. **Grave Identifier:** Grave 1. **Grave type:** no visible burial container, WNW-ESE orientation (parallel with the building wall), laid on its back. **Skeletal information:** male, 30-40 years old. **Grave goods:** Round bronze belt buckle dated between 13^th^ and 15^th^ century and a plain bronze ring ^[23]^. **Dating:** radiocarbon: 1161-1264 cal CE (847±27 BP, UBA-49326); artifacts, stratigraphy: 12^th^-14^th^ century CE. **Additional information**: the skeleton was partially excavated: his left half lies beneath the foundation of the apse and his legs outside the excavation limits.

**Ancestry summary**: Anatolia Roman+Eastern Europe.

- **Genetic Identifier:** I34980. **Grave Identifier:** Grave 17. **Grave type:** no visible burial container, SW-NE orientation, laid on her back with arms extended along the body. **Skeletal information:** female, over 40 years old. **Grave goods:** none. **Dating**: stratigraphy: 12^th^-14^th^ century. **Additional information**: grave orientation was not parallel with the walls of the chapel in front of which she was buried. She has been genetically determined to be the mother of the male in Grave 1.

**Ancestry summary**: Anatolia Roman+Eastern Europe.

- **Genetic Identifier:** I35014. **Grave Identifier:** Grave 10. **Grave type:** no visible burial container, WNW-ESE, laid on his back with his arms extended along the body. Orientation (parallel to the wall). Disturbed**. Skeletal information:** male, 20-30 years old. **Grave goods:** none. **Dating**: stratigraphy: 11^th^-12^th^ century. **Additional information**: individual buried in a grave pit in front of the southern wall of the nave. The skeleton was partially disturbed by more recent burials.

**Ancestry summary**: Eastern European-related.

- **Genetic Identifier:** I35095. **Grave Identifier:** Grave 23. **Grave type:** no visible burial container, SSW-NNE orientation (parallel to the façade), was laid on her back with arms extended along the body. **Skeletal information:** female, 30-40 years old. **Grave goods:** a pair of plain bronze circlets near the head. **Dating**: artifacts, burial location, stratigraphy: 11^th^-12^th^ century. **Additional information:** the individual was buried in front of the chapel, with her head near the entrance. Lower part of the right leg partially beneath the foundation. The type of bronze links is documented from the 9^th^ century until the late medieval period ^[22]^

**Ancestry summary**: Balkans Iron Age+Anatolia Roman+Eastern Europe.

- **Genetic Identifier:** I35010. **Grave Identifier:** Grave 25. **Grave type:** no visible burial container, NNE-SSW orientation, laid on her back with arms extended along the body. **Disturbed. Skeletal information:** female, 30-40 years old. **Grave goods:** bronze finger ring found near the right hand and two pieces of jewelry near the head: plain bronze circlet, bronze single-beaded earring. **Dating**: artifacts, stratigraphy: 11^th^-12^th^ century. **Additional information:** individual buried in a grave pit in front of the chapel, with her head near the entrance. Skeleton was partially disturbed. Plain bronze circlet can be placed in a wide timeframe from the 9^th^ century to the late medieval period. Bronze single-beaded earring / temple ring and bronze finger ring can be dated between the second half of the 9^th^ and the early 11^th^ century ^[22]^.

**Ancestry summary**: Balkans Iron Age+Anatolia Roman+Eastern Europe.

- **Genetic Identifier:** I35008. **Grave Identifier:** Grave 3. **Grave type:** no visible burial container, WNW-ESE orientation (perpendicular to the façade), laid on his back with arms placed on his stomach. **Skeletal information:** male, over 45 years old. **Grave goods:** Underneath his right knee, a leather bag with nine pieces of silver coins from the second half of the 17^th^ century was found. **Dating**: After 1696 CE based on the coins. **Additional information**: Individual buried in front of the chapel.

**Ancestry summary**: Balkans Iron Age+Anatolia Roman+Eastern Europe.

**1.7 Jagodnjak-Krčevine-Selska Bara (Croatia)**

Contact person(s): Dinko Tresić Pavičić, Anita Dugonjić

**Geographic Information**: The Jagodnjak–Krčevine-Selska Bara site is situated in the Croatian part of Baranja, near the town of Beli Manastir and some 5 km kilometres from the Drava river, some 35 km from where it empties into the Danube.

**Brief settlement history**: This multi-layered archaeological site contains traces of human activities dated to the Neolithic period, the middle Bronze Age, younger Iron Age, Antiquity, and the Early Medieval period ^[24]^. Important archaeological vestiges from the Early Medieval period include the settlement remains, and a cemetery (including thirty-six graves) situated alongside the western edge of the investigated area. Research to date indicates that the site was a small agricultural settlement in the early Middle Ages; physical anthropological study has identified in the individuals multiple osteological signs of intense labor in the course of everyday life, consonant with activities expected in field or stock-raising work ^[25]^.

**Excavation History**: Rescue archaeology at the site was carried out from October 2014 to August 2015 by Kaducej Ltd. The grave goods show typical characteristics of finds discovered throughout the Carpathian Basin during the Avar period, specifically the first and second Avar Khaganate (end of the 6^th^ century–first half of the 9^th^ century) ^[24]^.

**Summary of sampled materials**: The sampled individuals were excavated at the Early Medieval necropolis.

We sampled three individuals from this cemetery:

- **Genetic Identifier:** I26745. **Grave Identifier:** grave 14C. **Grave type:** no visible burial container, E-W orientation, laid on its back with arms along the body, with head to the east. **Skeletal information:** female, 1.5-2 years old. **Grave goods:** none. **Dating**: radiocarbon date 670-775 cal CE (1275±20 BP, PSUAMS-12035). **Additional information**: Multiple burial containing three individuals. The sampled child was placed between the legs of two unsampled adult skeletons who were oriented W-E (with the heads to the west).

**Ancestry summary**: Balkans Iron Age+Eastern Europe.

- **Genetic Identifier:** I26746. **Grave Identifier:** grave 52. **Grave type:** no visible burial container, W-E orientation, laid on his back with forearms placed over the pelvis, with head to the west. **Skeletal information:** male, over 50 years old. **Grave goods:** an iron buckle loop in the ribs on the right side and next to the right elbow. **Dating**: grave finds and the chronology of the cemetery activity: 600-850 CE. **Additional information**: animal bones were also present, testifying to a funerary ritual which may have involved a feast and/or food offering.

**Ancestry summary:** Anatolia Roman-related.

- **Genetic Identifier:** I26747. **Grave Identifier:** grave 27. **Grave type:** no visible burial container, orientation, lying on her back with arms extended along the body, with head to the west. **Skeletal information:** female, 35-45 years old. **Grave goods:** none. **Dating**: radiocarbon dates 671-774 cal CE (1280±20 BP PSUAMS-11913) and 655-737 cal CE (1320±22 BP; DeA-27897).

**Ancestry summary:** Balkans Iron Age+Eastern Europe.

**1.8 Jakovo, Kormadin (Serbia)**

Contact person(s): Anita Dugonjić, Maja Bunčić

**Geographic Information:** The site is located in Jakovo near Surčin in the south-east of Syrmia (modern Serbia).

**Brief settlement history:** Apart from the large late Neolithic / early Eneolithic (Vinča) settlement, the site is also significant for the cemetery from the end of the 5^th^ and the beginning of the 6^th^ century, where finds indicating Germanic cultural influence such as weapons (swords), knives, buckles and combs were discovered ^[26]^.

**Excavation History**: The site of Kormadin was recognized as a key archaeological site at the beginning of the 20^th^ century, thanks to the discovery of a large prehistoric settlement and a cemetery from the Migration period. The Archaeological Museum in Zagreb (then the Archaeological Department of the National Museum) was the patron of the excavations conducted from 1902 to 1906 by a local teacher from Surčin, Ante Poturičić. The richness of the site aroused the interest of Josip Brunšmid, director of the Archaeological Department of the National Museum, and he also conducted research at the same location on two occasions (1904 and 1905). Information about the excavations and finds are preserved in the correspondence of A. Poturičić with J. Brunšmid. Apart from basic information about the excavation site and very general information about the features, the letters are far from the necessary archaeological documentation that would enable the recognition of exact positions and the contexts of archaeological features, graves and their finds. Thus, although information on grave goods was recorded, it is not possible to associate each find with a specific grave.

The excavations at the site began again in 1956 and lasted until 1958 in order to protect the site during construction. The rescue archaeological excavation was led by the Homeland Museum of Zemun. The latest excavation at the site was conducted in 2008 under the leadership of the Department of Archaeology of the Faculty of Philosophy in Belgrade.

**Summary of sampled materials:** Of the graves excavated at the beginning of the 20^th^ century, Poturičić collected grave goods but kept only skulls of the skeletons, which are currently in the Archaeological Museum in Zagreb, but without proper numbering and only partial information ^[27–29]^.

We sampled four petrous bones from four different skulls collected by Poturičić and Brunšmid:

- **Genetic Identifier:** I27295. **Grave Identifier:** Burial 1903/30, skull No. 1; excavated by A. Poturičić. **Grave type:** unknown. **Skeletal information:** female, 5-7 years old. **Grave goods:** unknown. **Dating**: grave goods found at this cemetery: 450-550 CE. **Additional information:** healed cribra orbitalia in both orbits.

**Ancestry summary**: Balkans Iron Age+Central/Northern Europe+Pontic-Kazakh Steppe.

- **Genetic Identifier:** I27296. **Grave Identifier:** Burial 1904/09, No. 9; excavated by J. Brunšmid. **Grave type:** unknown. **Skeletal information:** male, 30-40 years old. **Grave goods:** unknown. **Dating**: grave goods found at this cemetery: 450-550 CE; radiocarbon: 435-587 calCE (1545±20 BP, PSUAMS-13193). **Additional information:** no pathologies observed.

**Ancestry summary**: Balkans Iron Age+Central/Northern Europe+Pontic-Kazakh Steppe.

- **Genetic Identifier:** I27297. **Grave Identifier:** Burial 1904/16, No. 5; excavated by J. Brunšmid. **Grave type:** unknown. **Skeletal information:** male, over 40 years old. **Grave goods:** unknown. **Dating**: grave goods found at this cemetery: 450-550 CE; radiocarbon: 440-599 calCE (1530±20 BP, PSUAMS-13194). **Additional information:** button osteoma on the right parietal bone.

**Ancestry summary**: Balkans Iron Age-related.

- **Genetic Identifier:** I27298. **Grave Identifier:** Burial 1904/15, No. 11/Ind. B; excavated by J. Brunšmid. **Grave type:** unknown. **Skeletal information:** female, over 45 years old. **Grave goods:** unknown. **Dating**: grave goods found at this cemetery: 450-550 CE. **Additional information:** no pathologies observed.

**Ancestry summary**: Balkans Iron Age-related.

**1.9 Lepenski Vir (Serbia)**

Contact person: Clive Bonsall

**Geographic Information:** The site is situated on the right bank of the River Danube in the Iron Gates Gorge.

**Brief settlement history**: The site of Lepenski Vir is renowned for its exceptional archaeological record of Mesolithic to Early Neolithic settlement, including abundant and well-preserved architectural remains, boulder artworks, and over 200 burials. Lepenski Vir lies 37 km downstream from the medieval fortress of Golubac, which was built on the site of an earlier Roman *castrum* on the south bank of the Danube at the entrance to the Iron Gates Gorge. A short distance to the south of Lepenski Vir lies the now submerged Roman fortress at Boljetin (ancient *Smorna*) occupied from the 1^st^ to the 6^th^ century CE, and subsequently used as a medieval (9^th^ century) burial ground where 97 graves were excavated.

**Excavation History:** The site was extensively excavated from 1965 to 1969 during construction of the Iron Gates I hydroelectric plant and dam which resulted in the total or partial flooding of archaeological sites throughout the Gorge. In historical times the Danube served as a natural frontier between competing powers, and military installations from the Roman, Migration and Ottoman periods abound in this region.

**Summary of sampled materials**: While the overwhelming majority of the burials found at Lepenski Vir relate to the Mesolithic–Early Neolithic (c. 9000–5500 cal BCE), human remains assigned to later periods were also unearthed. We report genetic data from two graves sampled in May 2000 at the Department of Archaeology, University of Belgrade. The first one is burial 18, a group of disarticulated bones recovered from a disturbed context near the ruins of a Roman watchtower in the central part of the site. It was later recognized that they comprised the remains of at least two adult individuals, named 18 and 18(1). Based on the relative robusticity of their cranial bones, 18 was interpreted as an adult female and 18(1) as an adult male ^[30]^.The second sample was a tooth labelled as ‘from burial 4’, radiocarbon dated to the medieval period. The nature and location of the original burial are uncertain, but it is probably not the crouched inhumation illustrated as ‘burial 4’ by Srejović (1969: plate 73) ^[31]^ and assigned to Lepenski Vir phase III (Neolithic).

Sampled Graves:

- **Genetic Identifier:** I4664. **Grave Identifier:** Burial 18. **Grave type:** disturbed context. **Skeletal information:** male, adult. **Grave goods:** none. **Dating**: radiocarbon 130-320 cal CE (1825±25 BP, OxA-25217) ^[32]^. **Additional information**: The sample taken for aDNA was a fragment of compact bone from a left ulna labelled as coming from burial 18. The ulna was present in the collection in 2000, when the sample was taken, but it was not among the bones from burial 18 examined by Roksandic (1999) ^[30]^, and so it is not clear if the ulna relates to burial 18, 18(1) or a third individual.

**Ancestry summary:** excluded from individual ancestry analysis based on low SNP count.

- **Genetic Identifier:** I5404. **Grave Identifier:** Burial 4. **Grave type:** undetermined type and orientation (e.g., Srejović 1969: pl. 73) ^[31]^. **Skeletal information:** male, adult. **Grave goods:** unknown. **Dating**: radiocarbon date 1432-1611 cal CE (421±23 BP, OxA-25212). **Additional information**: This individual is represented in our sampling by two teeth selected among human remains labelled as ‘burial 4’. Published accounts of burial 4 (e.g., Srejović 1969: pl. 73) ^[31]^ refer to a crouched inhumation covered by stone slabs that was assumed to date to the Early Neolithic. The fifteenth-century CE date for burial 4 is (within error) identical to the calibrated ^14^C ages of three well-documented medieval burials from Lepenski Vir (burials 29, 30 and 62), and his strontium isotope ratio is also very similar to other Medieval burials from the site ^[31]^ (such as 30, 49 and 62), indicating that they were immigrants who originated from at least two geologically distinct regions beyond the Iron Gates Gorge. The genetic profile is also consistent with a 15^th^-century date.

**Ancestry summary**: Balkans Iron Age+Anatolia Roman+Eastern Europe.

**1.10 Mala Metaljka (Croatia)**

Contact person(s): Ana Đukić

**Geographic Information:** The site is located in the Ogulin-Plaški region, on the saddle of the hill between the modern village of Trojvrh and Velika Metaljka, some 5 km from the Roman road to Senj, on the Adriatic, ca. 40 km to southwest.

**Brief settlement history**: The larger area of Trojvrh includes three hills and connected ravines: Veliki Vrh, Velika Metaljka and Mala Metaljka. Veliki Vrh and Velika Metaljka are defined as hillforts, while Mala Metaljka is considered to be the necropolis used by the inhabitants of, presumably, both settlements.

**Excavation History**: The excavations conducted in 1982 by the Archaeological Museum in Zagreb revealed four intact graves and 11 disturbed burials, most of which were dated to the Early Iron Age based on associated grave goods, a total of 12 finds. The excavations were limited to a very small area due to fear of mines left over from World War II when Mala Metaljka was used as a military stronghold. The stone constructions of the graves were almost completely destroyed, and skeletal remains were mixed with prehistoric pottery and material dating to the Roman period ^[33,34]^.

**Summary of sampled materials**: The analysed burial was excavated at the Mala Metaljka necropolis in 1982 and dated to the Late Roman period:

- **Genetic Identifier:** I24343. **Grave Identifier:** Grave 4. **Grave type:** no visible burial container. **Skeletal information:** female. **Grave goods:** none. **Dating**: radiocarbon: 433-578 cal CE (1550±20 BP, UCIAMS-179779). **Additional information**: The human remains were scattered and mixed with archaeological material dating to different periods (Iron Age, Roman period) but based on analogies with similar graves from the region, the individual was probably placed directly into the soil, on her back, with arms outstretched alongside the body.

**Ancestry summary**: excluded from analysis dataset based on possible mtDNA contamination.

**1.11 Mediana (Serbia)**

Contact person(s): Ilija Mikić

**Geographic Information:** Mediana has been identified in a Roman archaeological site located on the left bank of the river Nišava and on the old Niš-Pirot road near the village of Brzi Brod (Serbia). The vestiges are located approximately 4.5 km east of the Ottoman Niš Fortress, built on top of the ancient town of Naissus.

**Brief settlement history**: The extensive late Roman ruins about 5 km east of ancient Naissus (mod. Niš, Serbia) have been identified with Naissus’ suburb Mediana. Naissus began as an ancient Thracian settlement that developed under the Roman Empire, particularly in the 4^th^ century, when emperors and their courts were frequent visitors. Constantine I was born at Naissus, and archaeologists have suspected he may have been responsible for building the luxurious residential villa excavated at this site. The site was located on the old Roman road, a *via publica* that passed through the diocese of Dacia linking *Singidunum* (Belgrade) *Viminacium, Naissus,* and *Serdica* (Sofia, Bulgaria) ^[35]^, and connecting the Danube frontier with the imperial capital, Constantinople, as well as to the Adriatic. The Huns destroyed Naissus in 441; possibly restored in the 6^th^ century, it was conquered by the Avars ca. 613.

**Excavation History**: Although the Roman necropolis has not been yet explored, several archaeological interventions starting in 1961 have discovered burials dated to the Migration and medieval periods, most of them at the so-called “Southern Necropolis”. The individuals analyzed here were discovered and excavated in a different area of the site, west of the villa, in the process of constructing access paths for the future archaeological park around the villa complex. The detailed history of the villa complex, its transformations and its abandonment remain to be discovered.

**Summary of sampled materials**: Archaeological excavations in 2000-2001 unearthed 4 graves (Graves 34 to 37) ^[36]^ west of the villa with the peristyle ^[35,36]^. Graves 34 (located in Sounding 4) and 35 (located at the extension of Sounding 4) were sampled for this study. The general archaeological and historical context have suggested to some that both individuals belonged to the Gothic cultural circle ^[35]^. Both individuals present artificially deformed skulls (Figure 1 below), a non-Roman custom which scholars have long associated with migrating Eurasian populations and which became fashionable among various groups in southeastern Europe in the 5^th^ century ^[37,38]^. The skull bandaging method observed for these two individuals also corresponds to other examples of cranial deformation previously observed in necropolises characterized as “Germanic” at Viminacium ^[39]^.

Sampled Graves:

- **Genetic Identifier:** I15549. **Grave Identifier:** G-34. **Grave type:** the tomb was constructed using recycled Roman bricks. Skeleton in outstretched position, with hands alongside the body. W-E orientation, head in the west ^[40]^; artificially deformed skull. **Skeletal information:** male, under 21 years old. **Grave goods:** a comb, a lunular bronze pendant and a bead of glass paste. **Dating**: grave goods: assigned by excavators to late 4^th^ to early 5^th^ century; radiocarbon: 259-409 cal CE (1705±15 BP, PSUAMS-8557).

**Ancestry summary:** Balkans Iron Age+Central/Northern Europe+Pontic-Kazakh Steppe.

- **Genetic Identifier:** I15550. **Grave Identifier:** G-35. **Grave type:** the tomb was carved into a stone column from the end of the 4^th^ century–beginning of the 5^th^ century. This column had belonged to the latest construction phase of the villa and is oriented quite differently in relation to other architectural units. Skeleton in outstretched position, with hands alongside the body. W-E orientation, head in the west ^[40]^. **Skeletal information:** male, 40-45 years old. **Grave goods:** This tomb contained a bronze coin (held in the right hand of the skeleton), an iron wire, and an iron knife. **Dating**: general archaeological context: ca. 350-450 CE.

**Ancestry summary:** excluded from analysis dataset based on low SNP count.


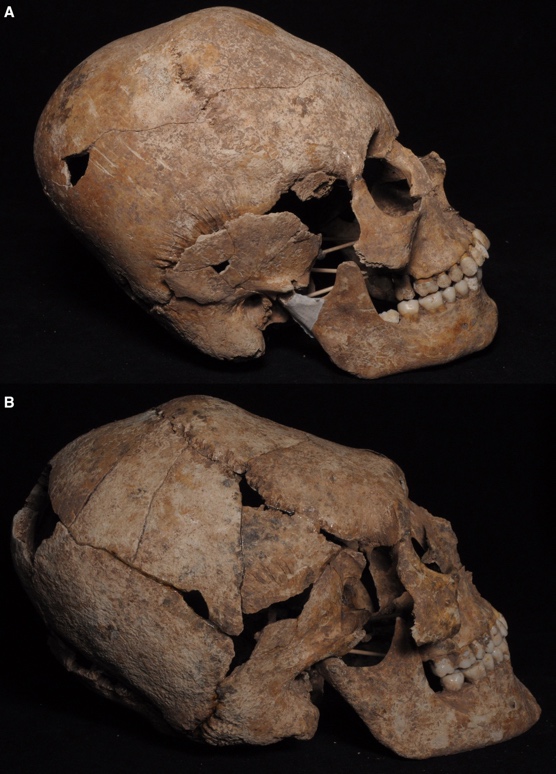


**Figure 1. Elongated skulls unearthed at the Mediana archaeological site**. (A) Grave 34 (I15549) and (B) grave 35 (I15550).

**1.12 Mlynská Dolina, Bratislava district (Slovakia)**

Contact persons**:** Alena Šefčáková, Juraj Bartík, Zdeněk Farkaš, Pavol Jelínek

**Geographic Information:** The area of archaeological research at Bratislava–Mlynská Dolina was spread over Staré Grunty, in the district of Karlova Ves, on the lower and higher middle terraces of the Danube River. To the south, investigation was bounded by the route of the original Botanická Street, which today reaches into the area of the Botanical Garden; to the east by the steep slope to the Vydrica Creek, which in the past meandered at the bottom of the Mlynská Dolina before emptying into the Danube River and to the north by the line of Ilkovičová Street, the part leading to the entrance to the buildings of the Faculty of Natural Sciences, Komenský University ^[41]^.

**Excavation History:** This area has been known as an archaeological site since at least the middle of the 20^th^ century ^[41]^, when fragments of ceramics from the Neolithic Period (younger Linear Pottery (LBK) and the Želiezovska group) and the Late Latène Period were recovered. On the grounds of the Botanical Garden, a part of the Early Medieval burial site from the 9^th^ century was uncovered. At the beginning of the 1980s, a larger Lengyel culture settlement was documented. Later, the northern part of a large settlement from the end of the Old and Middle Neolithic (Linear Pottery, LBK) and pits of the Middle Danube Mound Culture were discovered in areas A and B. Similar to the Medieval cultural layer, they crossed the northern edge of the original Neolithic settlement area.

**Summary of sampled materials:** In the years 1984-1990, under the leadership of B. Egyházy-Jurovská and Z. Farkaš, the Slovak National Museum - Archaeological Museum in Bratislava carried out rescue archaeological research in the lower part of the Mlynská Dolina in Bratislava, at the site of the construction of the northern approach of the Lafranconi highway bridge over the Danube River ^[41]^.

In 1984, 12 objects from the period of younger Linear Pottery (LBK), the Želiezovska type, the Middle Danube Mound culture and rare sherds from the Late La Tène Period were discovered, as well as cultural layers with finds from the 9^th^-16^th^ centuries. Moreover, an inhumation grave of a man in a crouched position was found which was assumed to be from the Neolithic ^[42]^. In 1986, two more children's graves with associated Neolithic objects were uncovered (Farkaš 2002). In subsequent research seasons, objects from the Neolithic, Eneolithic, and Bronze Age, as well as the Middle Ages and the modern period were found ^[41]^.

We sampled one Early Medieval grave:

- **Genetic Identifier:** I4803 **Grave Identifier:** Sample Inv. No. 7773. **Grave type:** skeletal grave in a separate burial pit, E-W orientation. **Skeletal information:** male, 35-45 years old. **Grave goods:** none. **Dating**: radiocarbon date 708-884 cal CE (1215±20 BP, PSUAMS-4351). **Additional information**: Grave found in 1986 in a separate grave pit north of the Neolithic inhabited area in the lower part of Bratislava–Mlynská Dolina on the right bank of Vydrica Creek ^[43]^. The individual was lying in a crouched position on his left side. This find, along with parts of other human remains in the secondary deposition, does not exclude the existence of a funeral area. The skeleton was damaged but belong to a robust male (identified osteologically and genetically), about 171 cm tall, with osteological markers of strong muscular activity. On his spine one can observe degenerative changes in the form of *spondylosis deformans* of degree 2 (up to 3 mm), Schmorl’s nodes and *spondylarthrosis;* and traces of a healed fracture are visible on the right *radius*.

**Ancestry summary**: Eastern European-related ancestry.

**1.13 Nuštar, Dvorac (Croatia)**

Contact person(s): Anita Rapan Papeša, Petra Rajić Šikanjić, Zrinka Premužić.

**Geographic Information:** The site is located near the family Khuen-Belasi castle in the village of Nuštar (Vukovar – Srijem county, eastern Croatia). Nuštar is 5 km northeast of the modern town of Vinkovci, and 25 km souteast of Osijek (see Osijek) on the Roman road network between the Danube and the Adriatic.

**Excavation History:** In 2011, the Municipal Museum of Vinkovci carried out a rescue excavation at the construction site of the new football (soccer) field, when part of a Late Avar Period cemetery was investigated. Altogether, 196 graves with 188 individuals organized into 10 rows have been excavated and dated archeologically to the 8^th^ and early 9^th^ century CE. Radiocarbon dates place three individuals in the 7^th^-9^th^ century, 8^th^-9^th^ century and 8^th^-10^th^ century with 95% confidence.

**Summary of sampled materials:** Graves were visible as dark rectangular patches in yellow loess soil. All burials were oriented west–east, and seven multiple burials were recorded. The deceased were buried in supine position on their back, with arms laid next to the body with only a few exceptions. Different wooden constructions affected the degree of skeletal preservation. Over 1500 small finds came from 175 graves and we can divide those into two main categories: traces of food offerings (animal bones and pots) and grave goods (costume elements, personal belongings and tools) ^[44]^. The available radiocarbon dates so far place this site in the 8th-10th century CE: skull with trepanation (unsampled for this study; context lost due to construction work) dated to 664-821 cal CE (1277±29, UBA-25620), Grave 45 (unsampled for this study) dated to 702-881 cal CE (1230±20 BP, PSUAMS-12039) and Grave 52 (sampled for this study) dated to 771-947 cal CE (1175±15 BP, PSUAMS-12038); all other dates are based on the small finds ^[44–50]^.

We sampled 9 individuals from this necropolis:

- **Genetic Identifier:** I28388. **Grave Identifier:** Grave 36. **Grave type:** deceased in grave with beam construction, W-E orientation, lying on its back. **Skeletal information:** male, 18-20 years old. **Grave goods:** dark brown pot made on a slow pottery wheel, single-edged iron knife, iron belt buckle and cast bronze fittings of a belt set: trapezoidal buckle, main strap end decorated with a flat leaf tendril, propeller mount, ten main belt mounts with pendants decorated with lilies, four belt hole guards decorated with flat leaf tendrils, eleven side strap mounts decorated with a geometrical pattern, side strap end decorated with two griffins. **Dating**: grave goods: according to the belt set parts the grave belongs to Zábojník’s SS III phase (750-780 CE) ^[51]^; stratigraphy: horizontal stratigraphy of the cemetery suggests the beginning of that phase. **Additional information**: Rectangular grave-pit (2.68 x 0.95 x 1.11 m) with deeper ends under the head and feet, and three pairs of vertical beams.

**Ancestry summary**: Balkans Iron Age-related.

- **Genetic Identifier:** I28390. **Grave Identifier:** Grave 52. **Grave type:** no visible burial container, W-E orientation, lying on her back with arms extended along the body and fingers on the pelvis. **Skeletal information:** female, 35-50 years old. **Grave goods:** brown pot made on a slow pottery wheel, square undecorated needle case, oval bronze earring with rhombic cross section and side decoration, necklace made of glass beads shaped like blue and green translucent watermelon seeds. **Dating**: grave goods: earring Type X after Čilinska (725-800 CE) ^[52]^, beads of “*melonenkern*” type that appear throughout 8^th^ century (but translucent blue and green appear before black opaque ones), suggesting an archeological date ca. to 725-750 CE; stratigraphy: cemetery horizontal stratigraphy would fit 725-750 CE; radiocarbon date: 771-947 cal CE (1175±15 BP, PSUAMS-12038). **Additional information**: rectangular grave-pit with deeper ends under the head and feet (2.45 x 1.02 x 1.03 m). She is the mother of the individual 50A in grave 50.

**Ancestry summary:** Balkans Iron Age+Anatolia Roman+Eastern Europe.

- **Genetic Identifier:** I28392. **Grave Identifier:** Grave 41. **Grave type:** no visible burial container, W-E orientation, lying on her back with arms extended along the body and fingers on the pelvis. **Skeletal information:** female, 20-35 years old. **Grave goods:** brown pot made on a slow pottery wheel, oval bronze earring with rhombic section and metal bead pendant, single edged iron knife, trapezoidal iron buckle, rounded spindle whorl with horizontal grooves.. **Dating**: grave goods: analogous types suggest this pot belongs to SPA I period (~710-740 CE) after Daim ^[53]^; similar reasoning for the earring; stratigraphy: cemetery horizontal stratigraphy agrees with a 710-740 CE date. **Additional information**: rectangular grave-pit with deeper ends under the head and feet (2.10 x 0.87 x 0.87 m).

**Ancestry summary**: Balkans Iron Age-related.

- **Genetic Identifier:** I28393. **Grave Identifier:** Grave 161. **Grave type:** no visible burial container, W-E orientation, lying on her back. **Skeletal information:** female, 20-35 years old. **Grave goods:** brown pot made on a slow pottery wheel, Roman-type ‘strongly profiled fibula ^[54]^, two bronze bracelets made of thick wire with slightly wider ends and punched dot decoration, biconical lead spindle whorl, single-edged iron knife, pair of bronze earrings connected with bronze chain, 455 necklace beads: millet-like, watermelon-seed like, and segmented), Roman bronze finger ring, hollow silver bead. **Dating**: analogous examples suggest this pot type belongs to SPA III period (770-800 CE) after Daim ^[53]^; similarly for earrings and mixed necklace; stratigraphy: cemetery horizontal stratigraphy would fit a 770-800 CE date. **Additional information:** rectangular grave-pit with deeper ends under the head and feet (2.45 x 0.75 x 1.05m).

**Ancestry summary**: Balkans Iron Age+Anatolia Roman+Eastern Europe.

- **Genetic Identifier:** I28394. **Grave Identifier:** Grave 57. **Grave type:** no visible burial container, W-E orientation, lying on his back with arms crossed over the pelvis. **Skeletal information:** female, 12-14 years old. **Grave goods:** pair of oval bronze earrings with rhombic cross section and bead pendants, necklace (of 20 beads: watermelon seed-like, millet-like, segmented and oval with melted ornament). **Dating**: earring Type X after Čilinska (725-800 CE) ^[52]^, similar mixed necklaces belong to the latest Avar culture phases (750-800 CE); stratigraphy: according to the horizontal stratigraphy of the cemetery and graves in the vicinity that were dated through belt sets, this grave belonged to phase SS III / SS IV (750-825 CE) after Zábojník ^[51]^. **Additional information:** rectangular grave-pit, narrowing with steps (1.80 x 0.90 x 0.44 m).

**Ancestry summary**: Balkans Iron Age+Anatolia Roman+Eastern Europe.

- **Genetic Identifier:** I28397. **Grave Identifier:** Grave 139. **Grave type:** no visible burial container, W-E orientation, lying on its back with arms extended along the body and head to the West. **Skeletal information:** male, 35-50 years old. **Grave goods:** trapezoidal iron buckle, single-edged iron knife. **Dating**: stratigraphy: according to horizontal stratigraphy of the cemetery and graves in the vicinity that were dated through belt sets, this grave belongs to phase SS III (750-780 CE) after Zábojník ^[51]^. **Additional information:** rectangular grave-pit (2.41 x 0.60 x 0.18 m).

**Ancestry summary**: Balkans Iron Age+Anatolia Roman+Eastern Europe.

- **Genetic Identifier:** I28398. **Grave Identifier:** Grave 81. **Grave type:** no visible burial container, W-E orientation, lying on her back with arms extended along the body. **Skeletal information:** female, 35-50 years old. **Grave goods:** brown hand-made pot, biconical spindle whorl. **Dating**: stratigraphy: according to analogies this pot type belongs to SPA II period (740-770 CE) after Daim ^[53]^; stratigraphy: cemetery horizontal stratigraphy fits a 740-770 CE date. **Additional information:** rectangular grave-pit with deeper ends under the head and feet (1.97 x 0.58 x 0.67 m).

**Ancestry summary**: Balkans Iron Age+Anatolia Roman+Eastern Europe.

- **Genetic Identifier:** I28400. **Grave Identifier:** Grave 118. **Grave type:** no visible burial container, W-E orientation, lying on his back with legs and arms bended (fetal position). **Skeletal information:** male, 20-35 years old. **Grave goods:** none. **Dating**: stratigraphy: based on horizontal stratigraphy of the cemetery and graves in the vicinity that were dated through belt sets, this grave belongs to phase SS III (750-780 CE) after Zábojník ^[51]^. **Additional information:** Rectangular grave-pit (1.65 x 0.70 x 0.16 m).

**Ancestry summary**: Balkans Iron Age+Anatolia Roman+Eastern Europe.

- **Genetic Identifier:** I34800. **Grave Identifier:** Grave 50 A. **Grave type:** no visible burial container, W-E orientation, lying on his back with head to the West; double burial. **Skeletal information:** 2 subadults; only A sampled: male, 10-12 years old. **Grave goods:** brown pot made on a slow pottery wheel, decorated with multiple wavy lines. **Dating: g**rave goods: according to analogies this type of pot belongs to SPA III period (770-800 CE) after Daim ^[53]^. **Additional information**: rectangular grave-pit with deeper ends under the head and feet, and three pairs of vertical beams and narrowing along longer edges (2.37 x 1.41 x 1.07 m). It contained two juvenile individuals lying parallel to each other, but due to the collapse of the grave construction bones were mixed. The unsampled individual (B) was 5-10 years old and was associated with two single-edged iron knifes. The sampled individual A was genetically the son of the woman individual in grave 52 analyzed above.

**Ancestry summary**: Anatolia Roman+Eastern Europe.

**1.14 Osijek (Croatia)**

Contact person(s): Tino Leleković

**Geographic Information:** The site is located at the Ban Jelačić Square, some 100 m outside the eastern Roman town gates of *Mursa* in modern Osijek (Osijek-Baranja County, Slavonia, Croatia). Mursa is located on the Drava River and the Roman road network connecting the Adriatic to the Danube frontier.

**Brief settlement history**: *Mursa* was a colony of veterans (*Colonia Aelia Mursa*) founded by Emperor Hadrian in 133 CE in the eastern part of Pannonia (Roman province *Pannonia Inferior*). As one of the largest settlements in the region, situated on the Drava River, *Mursa* was the civilian and administrative centre of this part of the border (*limes*) zone.

**Excavation History**: Tino Leleković from the Croatian Academy of Sciences and Arts in Zagreb led a rescue excavation in 2008 at the site. This excavation, covering an area of about 3000 m^2^, revealed stratigraphy that includes five different development phases. The earliest layer contained 101 incineration graves from the late 1^st^ and early 2^nd^ centuries CE, part of the cemetery that preceded the colony. In the 2^nd^ and 3^rd^ centuries, this area was a suburb. According to metal finds and coinage from burials, it was reused as a burial area in the 260s or 270s CE.

**Summary of sampled materials:** The analysed burials are from the eastern cemetery of the Roman town of *Mursa*. Archaeological excavation revealed 311 graves from the 3^rd^ century CE, some of which were sampled for this study. Retrieved grave goods show that these burials originate from a narrow time frame, indicating that this group of people died during a short period, possibly suggesting this group of burials formed during an epidemic or war ^[55,56]^.

Sampled graves:

- **Genetic Identifier:** I26753. **Grave Identifier:** grave 15. **Grave type:** no visible burial container, NW-SE orientation, lying on his back with the arms placed on the pelvis and the legs bent to the left. **Skeletal information:** male, adult. **Grave goods:** none. **Dating**: general chronology of the cemetery: 3^rd^ century. **Additional information**: the body was placed in a rectangular grave-pit 146 cm long.

**Ancestry summary**: Anatolia Roman-related.

- **Genetic Identifier:** I26752. **Grave Identifier:** grave 17. **Grave type:** no visible burial container, NE-SW orientation, double burial, apparently simultaneous ^[57]^. **Skeletal information:** adult male. **Grave goods:** a bronze box or inkpot containing coins of Gallienus (253-268 CE) and Valerianus (253-260 CE) minted in Viminacium, a glass vessel, and a ceramic jug. **Dating**: artifacts, coins, cemetery chronology: 3^rd^ century. **Additional information:** The burial includes a male and female skeleton placed in a rectangular grave-pit (d. 206 cm x 110 cm). The female skeleton had her hands resting crossed on her abdomen, and her feet folded. The male skeleton (analysed in this study) had his legs outstretched, his right arm outstretched, and his left arm resting on his chest holding the female skeleton by her right arm. Both individuals were buried with pieces of jewelery.

**Ancestry summary**: Balkans Iron Age-related.

- **Genetic Identifier:** I26754. **Grave Identifier:** grave 129. **Grave type:** no visible burial container, NE-SW orientation. **Skeletal information:** female, adult. **Grave goods:** several pieces of beads found around the neck and a bronze inkwell cover. **Dating**: general chronology of the cemetery, artifacts: 3^rd^ century. **Additional information:** The skeleton was in the pose of a person sleeping in a bed, positioned on the right side. The bronze inkwell cover was placed below the head.

**Ancestry summary**: Balkans Iron Age-related.

- **Genetic Identifier:** I26751. **Grave Identifier:** grave 227-B. **Grave type:** no visible burial container, NE-SW orientation; double burial, unclear whether simultaneous (Type I). **Skeletal information:** adult female. **Grave goods:** Two iron bracelets, two coins, iron knife, buckle. **Dating**: general chronology of the cemetery, artifacts: 3^rd^ century. **Additional information:** Double burial, most likely a male and female skeleton, recovered in a square shape grave-pit (d. 197 cm x 107 cm). The skeletons were on their backs, parallel to each other, with stretched legs. The right arm of the smaller skeleton was on the abdomen, and the left was outstretched next to the body. The larger skeleton was only partially preserved. The smaller (female) skeleton (sampled in this study) had a bronze bracelet on each arm. The grave goods of the larger skeleton included two coins discovered between the spine and the left elbow, an iron knife on the left thigh, and a buckle between the left arm and the spine.

**Ancestry summary**: Balkans Iron Age-related.

- **Genetic Identifier:** I26750. **Grave Identifier:** grave 350. **Grave type:** no visible burial container, NE-SW orientation; triple burial, apparently Type I ^[57]^. **Skeletal information:** female, adult. **Grave goods:** none. **Dating**: stratigraphy: 3^rd^ century. **Additional information:** Grave 350 includes three individuals buried in a rectangular pit (d.192 cm x 113 cm). The female skeleton (analysed in this study) was laid on her back, with the legs folded. The male skeleton was lying on his stomach with his legs outstretched. His hands were placed under his head as if asleep. The burial included an infant skeleton between the two adult skeletons. The poorly preserved child's skeleton had parts of the leg bones missing.

**Ancestry summary**: Balkans Iron Age-related.

**1.15 Pogorelec, Sisak (Roman *Siscia*) (Croatia)**

Contact person(s): Ivan Drnić, Željka Bedić

**Geographic Information**: Sisak is a modern town in continental Croatia, located some 50 km southeast of Zagreb at the confluence of three rivers: the Cupa, the Odra and the Sava on whose southern bank it sits, and on the important Roman road from *Emona* (Ljubjana) to *Sirmium* within the network of roads connecting the Adriatic coast to the Danube frontier.

**Brief settlement history**: During the 4^th^ century BCE the site was inhabited by an indigenous Pannonian community known from the ancient written sources as *Segestani* (hence the name of the settlement *Segesta* / *Segestica*) ^[58]^.The Romans conquered *Segestica* in 35 CE and established a military stronghold which gradually evolved into a civilian settlement; it was raised to the rank of colony during the reign of the emperor Vespasianus (*Colonia Flavia Siscia*) ^[58,59]^. It was one of the largest settlements in the Roman province of Pannonia with a river harbor, fortifications, baths, aqueducts, several cemeteries and a mint. During Diocletian’s rule it became the capital of the province Pannonia Savia ^[60,61]^.

**Excavation History**: continuous archaeological excavations at Pogorelec started in 2012 and are led by Archaeological Museum in Zagreb, with a total of 28 skeletal burials having been discovered at the western necropolis. Also, during the rescue excavations conducted in 2013 and 2014 in the western necropolis, the private company Arheolog d.o.o. uncovered 65 skeletal burials.

**Summary of sampled materials**: the western necropolis developed in the north-eastern section of modern Pogorelec, on the right bank of the river Kupa, next to the road which probably connected the bridge on the Kupa River, next to the western city walls, to the bridge where the Odra River empties into the Kupa ^[58,62]^. The finds and elements of costume from the grave goods excavated so far, as well as the burial rites (exclusively inhumations), date this necropolis generally to Late Antiquity, from the end of the 3^rd^ to the first half of the 5^th^ century CE.

We sampled seven individuals from the western necropolis:

- **Genetic Identifier:** I28446. **Grave Identifier:** Grave 5. **Grave type:** deceased in a grave with *tegulae* (roof tile) construction, N-S orientation. The skeleton was covered with several white sandstone tiles over the chest and legs. A small wire, 1.8 cm long, was found between the *tegulae*, and it may have been part of an earring. **Skeletal information:** female, 1.5-2 years old, laid on her back. **Grave goods:** two fragments of ribbed iron bracelets and six tiny cylindrical beads: four made of blue and two of white glass were found around the skull. **Dating**: end of the 3^rd^ to the first half of the 5^th^ century CE, based on the cemetery’s life span.

**Ancestry summary:** Balkans Iron Age+Central/Northern Europe+Pontic-Kazakh Steppe.

- **Genetic Identifier:** I26761. **Grave Identifier:** Grave 8. **Grave type:** no visible burial container, E-W orientation. **Skeletal information:** female, 35-40 years old, laid on her back. **Grave goods:** bronze finger ring on a dislocated finger of the left hand. **Dating**: end of the 3^rd^ to the first half of the 5^th^ century CE, based on the cemetery’s life span. **Additional information**: badly preserved skeleton.

**Ancestry summary:** Balkans Iron Age+Central/Northern Europe+Pontic-Kazakh Steppe.

- **Genetic Identifier:** I26762. **Grave Identifier:** Grave 9. **Grave type:** no visible burial container, NW-SE orientation. **Skeletal information:** female, 11-12 years old, curled on the right side and lying on a layer of potsherds. **Grave goods:** pottery fragments, two copper-alloy bracelets, an iron peg. **Dating**: end of the 3^rd^ to the first half of the 5^th^ century CE, based on the cementery’s life-span.

**Ancestry summary:** Balkans Iron Age+Central/Northern Europe+Pontic-Kazakh Steppe.

- **Genetic Identifier:** I28447. **Grave Identifier:** Grave 10. **Grave type:** no visible burial container, E-W orientation, placed on her back with arms extended along the body. **Skeletal information:** female, 40-50 years old. **Grave goods:** nine glass beads. **Dating**: end of the 3^rd^ to the first half of the 5^th^ century CE, based on the cemetery’s life-span.

**Ancestry summary:** Balkans Iron Age-related.

- **Genetic Identifier:** I26763. **Grave Identifier:** Grave 14. **Grave type:** no visible burial container, E-W orientation. **Skeletal information:** male, 30-55 years old, placed on its back with right arm extended along the body and the left placed on the right hip. **Grave goods:** amorphous lump of bronze. **Dating**: end of the 3^rd^ to the first half of the 5^th^ century CE, based on the cemetery’s life-span.

**Ancestry summary:** Balkans Iron Age-related.

- **Genetic Identifier:** I28448. **Grave Identifier:** Grave 17. **Grave type:** deceased in a grave with *tegulae* (roof tile) construction, N-S orientation. **Skeletal information:** female, 40-50 years old, placed on her back with orientation north-south with head to the north. **Grave goods:** two bone bracelets (left hand), two bronze earrings with glass beads on either sides of the skull, silver foil. **Dating**: end of the 3^rd^ to the first half of the 5^th^ century CE, based on the cemetery’s life-span.

**Ancestry summary:** Balkans Iron Age-related.

- **Genetic Identifier:** I26764. **Grave Identifier:** Grave 21. **Grave type:** Deceased in a wooden coffin, NW-SW orientation. **Skeletal information:** female, 40-45 years old, laid on her back with arms extended along the body. **Grave goods:** two ceramic vessels (a bowl and jug) and a glass *balsamarium,* elements of a costume (ten biconical glass beads (9 blue, 1 green )), two coins. **Dating**: after 348/350 CE based on coins and, before ca. 450 CE based on the cemetery’s life-span. **Additional information**: the glass *balsamarium* was laid next to the left leg, and the coins near the pelvis. Two coins were found next to the deceased woman’s pelvis (AE3 of emperor Constans, minted between 348 and 350 AD in Thessalonica, and AE2 of the usurper Caesar Decentius, minted in Aquileia in 351 AD, which date the grave after the middle or, possibly, the second half of the 4^th^ century CE). Above the deceased woman’s head there was a large clamp which was probably part of the coffin indicated by the wood traces.

**Ancestry summary:** Balkans Iron Age+Anatolia Roman.

**1.16 Pottenbrunn (Austria)**

Contact persons**:** Maria Teschler-Nicola

**Geographic Information:** Pottenbrunn is located about 50 km west of Vienna, south of the Danube, close to the Lower Austrian provincial capital St. Pölten. The 9^th^-century CE cemetery is located in a gravel pit, in “Parzelle” (“plot”) 1839/1.

**Excavation History:** The first graves were discovered in 1965; in 1966 rescue excavations by the Working Group for Prehistory and Early History (“Fundbergedienst der Österreichischen Arbeitsgemeinschaft für Ur- und Frühgeschichte”) unearthed 61 graves ^[63,64]^. The excavations were continued in 1973 and 1974 by staff members of the Anthropological Department of the Natural History Museum of Vienna (Johann Jungwirth, Helmut Windl and Paul Spindler), who excavated 123 further burials ^[65–67]^.

**Summary of sampled materials:** The material culture of the cemetery has been identified as Slavic (influenced by the “Köttlach” and contemporary Bavarian material culture; Carolingian-influenced pottery). The graves are mostly west-east oriented (exactly west-east: 38%; the others differ slightly from this orientation), undisturbed and equipped with similar grave goods: females and children were buried with small iron knives, men's graves contained iron sickles, awls, and long knives; other items recovered are iron bands for wooden containers, bronze and silver adornments (ear- and finger rings, bracelets, glass beads enclosed by bronze or iron wire). Remains of wooden coffins were often found. Out of the total of 184 archaeologically verified burials the skeletal remains of 199 individuals were recovered. Most of the graves are single burials, but there are also 8 double burials, 1 triple burial and 9 successive multiple burials (McCormick Type II,“Nachbestattungen” ^[57]^). Age at death estimations indicated 104 subadults and 95 adults (48 males and 47 females) ^[68]^. The skeletons are preserved at the Natural History Museum and were investigated in further studies, e.g., they were included in the *European History of Health Project,* a large, multidisciplinary research project that recorded health indicators of skeletal remains of more than 15,000 individuals from the third to the end of the 19^th^ century recovered from more than 100 European localities ^[69]^. Data recording at Pottenbrunn remains revealed, e.g., the first case of lepromatous leprosy infection in Austria ^[70–72]^, and cases of a very rarely observed inherited metabolic disorder (Mucopolysaccharidosis) ^[73]^.

We report data from one individual from this Early Medieval cemetery:

- **Genetic Identifier:** I15232. **Grave Identifier:** Grave 123 (Inv. No 22451/1973); mus. Inv. 6330. **Grave type:** pit burial, W-E orientation. **Skeletal information:** male, 6 years old (Infans I/2) based on the mineralisation degree of the teeth (Fabrizii-Reuer and Reuer, 2001). **Grave goods:** above the thorax and the right knee of the individual, remnants of the coffin and near the left thigh an iron knife was observed (Jungwirth and Windl 1973:132). **Dating:** radiocarbon dating of the petrous bone analyzed for aDNA, 773-890 cal CE (1190±20 BP, PSUAMS-9000). **Additional information:** depth 115 cm; preserved are small thin-walled neurocranial fragments including the right temporal and isolated left petrous bone, fragments of the mandible and some teeth; the postcranium is represented by both femur shafts and a few vertebral arches.

**Ancestry summary**: Eastern European-related ancestry.

**1.17 Ravna village; probable ancient *Timacum Minus* (Serbia)**

Contact person(s): Sofija Petković, Nataša Miladinović-Radmilović, Dragana Vulović

**Geographic Information:** The site is located in the western Balkans in the vicinity of present-day Ravna, 10 km north of Knjaževac in eastern Serbia, on a tributary and some 60 km southwest of the Danube.

**Brief settlement history**: The Roman army was active on this site in the second half of the 1^st^ century CE: a stone auxiliary fortification defended the left (western) bank of the Beli (“White” ) Timok River, part of a network of tributary rivers of the Danube, probably from around the middle of the 2^nd^ century ^[74]^. The fort was on the important Roman road connecting the Danube frontier to the Adriatic coast, and has been plausibly identified with “*Timacum Minus*”, mentioned notably by the Peutinger Table, and alluded to in an inscription from Ravna ^[75]^; a small civilian settlement developed adjacent to the fort and included baths and temples from the 2^nd^ or 3^rd^ centuries CE. The fort may have been connected with Roman mines in the area ^[76,77]^. It was rebuilt ca. 300 and, again, in the second half of the 4^th^ century; apparently around 400 CE, it was at least partially damaged by fire; another destruction layer assigned to the middle of the fifth-century level is the latest ancient activity so far detected, although, in the 6^th^ century Procopius mentions fortification work at two sites in the region whose names seem to allude to the Timok River ^[78]^. A new settlement grew up in the ruins of the fort and yielded ceramics dated to the 11^th^ and 12^th^ centuries ^[74,79]^.

**Summary of sampled materials**: Archaeological excavations in Ravna have identified 4 different Late Roman/Medieval necropolises: 1- Kuline, located in the interior of the Roman fortress, 2-Slog, 3- Podina and 4- Ravanski (Zubanov). For this study we have sampled two of them: the medieval burials at Kuline and the late Roman ones at Slog.

**1.17.1 Kuline Necropolis (8^th^-11^th^ centuries)**

**Geographic Information:** see above.

**Excavation History:** The excavations of Kuline necropolis started in 1978. A total of nine graves were initially found, located in the central part of the abandoned Roman fortress ^[79]^. The cemetery is organized in rows, which are oriented in a west-east direction, and are formed by simple grave pits, except in two cases (G-3 and G-5) where river pebbles were found along the left side of the deceased^77^. The graves are unfurnished and, archaeologically, could be dated only by the abandonment of the fort, presumably in the fifth century, and the assumption of a link with the medieval settlement connected to the medieval burials on Slog hill, and whose traces were also detected in the abandoned fortress ^[79]^.

**Summary of sampled materials:** A total of 7 individuals were sampled; physical anthropological studies suggested genetic connections among them ^[79]^, and the recovery of their genomes shows that the adult males buried next to each other in Graves 2 and 3 were identical twins.

- **Genetic Identifier:** I15537. **Grave Identifier:** G-1. **Grave type:** no visible burial container, W-E orientation. **Skeletal information:** male, adult. **Grave goods:** none. **Dating**: settlement archaeological context and radiocarbon dates of other individuals buried in this cemetery: ca. 800-1000 CE.

**Ancestry summary:** Balkans Iron Age+Anatolia Roman+Eastern Europe.

- **Genetic Identifier:** I15538. **Grave Identifier:** G-2. **Grave type:** no visible burial container, pit was dug into the street’s paving stones, W-E orientation. **Skeletal information:** male, adult. **Grave goods:** none. **Dating**: radiocarbon: date 892-989 cal CE (1115±15 BP, PSUAMS-8592). **Additional information**: The genetic analysis shows that this individual is an identical twin of individual G-3, buried in the grave immediately south of G-2.

**Ancestry summary:** Southwestern European-related.

- **Genetic Identifier:** I15539. **Grave Identifier:** G-3. **Grave type:** no visible burial container, pit was dug into the street’s paving stones, W-E orientation. **Skeletal information:** male, adult. **Grave goods:** none. **Dating**: settlement archaeological context: 800-1000 CE; genetically the twin brother of the G-3, therefore, radiocarbon date: 892-989 cal CE (1115±15 BP, PSUAMS-8592). **Additional information**: The genetic analysis shows that this individual is an identical twin of individual G-2, buried in the next grave to the north.

**Ancestry summary:** Southwestern European-related.

- **Genetic Identifier:** I15540. **Grave Identifier:** G-4. **Grave type:** no visible burial container, W-E orientation. **Skeletal information:** female, adult. **Grave goods:** none. **Dating**: radiocarbon: 775-978 cal CE (1140±15 BP, PSUAMS-9670).

**Ancestry summary:** Balkans Iron Age+Anatolia Roman+Eastern Europe.

- **Genetic Identifier:** I15541. **Grave Identifier:** G-5. **Grave type:** no visible burial container, W-E orientation. **Skeletal information:** male, adult. **Grave goods:** none. **Dating**: settlement archaeological context and radiocarbon dates of other individuals buried in this cemetery: ca. 800-1000 CE.

**Ancestry summary:** Balkans Iron Age+Anatolia Roman+Eastern Europe.

- **Genetic Identifier:** I15542. **Grave Identifier:** G-6. **Grave type:** no visible burial container, W-E orientation. **Skeletal information:** male, adult. **Grave goods:** none. **Dating**: radiocarbon date 897-1021 cal CE (1075±15 BP, PSUAMS-8555).

**Ancestry summary:** Balkans Iron Age+Anatolia Roman+Eastern Europe.

- **Genetic Identifier:** I15543. **Grave Identifier:** G-7. **Grave type:** no visible burial container, W-E orientation. **Skeletal information:** male, adult. **Grave goods:** none. **Dating**: settlement archaeological context and radiocarbon dates of other individuals buried in this cemetery: ca. 800-1000 CE.

**Ancestry summary:** Balkans Iron Age+Anatolia Roman+Eastern Europe.

**1.17.2 Slog Necropolis (4^th^ – 6^th^ Centuries)**

**Geographic information:** About 400 west of the Roman fort, near Ravna; see above.

**Excavation History**: Part of the multi-phased late Roman and early medieval necropolis was explored about 400 m west of the fort in the course of three campaigns of rescue excavation on the eastern slope of the hill known as Slog, prompted by the reconstruction of the local Ravna – Debelica road in 1994–1996, on the west side of the road. A total of 140 graves were discovered, of which 72 date to the Late Roman period. According to the archaeologists, this necropolis developed in three successive phases (plans 6 and 7 of Petković, 2005) ^[74]^, from the middle of the 4^th^ to the middle of the 5^th^ century. The deduced phases are based on grave goods as indicators of cultural identity or their absence, and general historical considerations, and have the limitations associated with such datings. The excavators would distinguish phase I from ca. 350 to 380 CE, phase II from ca. 380 to 410 CE, and phase III from ca. 410 to 450 CE ^[77]^. A number of the graves were disturbed and objects removed, presumably in antiquity, complicating assessments based on grave goods. Nevertheless, the presence of military gear and weapons ^[77]^ among the grave furnishings, healed or perimortem wounds consistent with or certainly stemming from combat, including a trilobed arrowhead typical of steppe warriors that was possibly a cause of death (Grave 123), and markers of occupational stress indicate that many of these individuals were connected to warfare and the late Roman army. All the sampled individuals were genetically male with only one exception.

The medieval graves were dug in the same space as well as immediately west of the late Roman graves. Grave goods and historical considerations led the excavators to date these graves from the late 9^th^ into the 11^th^ century; the cemetery was only partially excavated. The medieval graves from this necropolis have not been included in this study.

Sampled Individuals:

- **Genetic Identifier:** I15551. **Grave Identifier:** G-91. **Grave type:** destroyed brick and stone construction; iron nails indicate a wooden coffin; disturbed; W-E orientation. **Skeletal information:** male, over 50 years old; healed possible weapon wound on the mentum **Grave goods:** none: the grave had been opened and grave goods presumably removed. **Dating**: archaeological: 350–380 CE (Phase I of necropolis); radiocarbon: 242-375 cal CE (1750±20 BP, PSUAMS-8561)

**Ancestry summary:** Northern Levant.

- **Genetic Identifier:** I15553. **Grave Identifier:** G-99. **Grave type:** no visible burial container, rectangular burial pit, W-E orientation; disturbed. **Skeletal information:** male, 30-35 years old. **Grave goods:** an amphora-shaped bronze belt-tip and fragments of bronze plating among disarticulated skeleton **Dating**: archaeological: belt tip: ca. 350-450; 350–380 CE (Phase I of necropolis).

**Ancestry summary:** Balkans Iron Age-related.

- **Genetic Identifier:** I15544. **Grave Identifier:** G-15. **Grave type:** no visible burial container, rectangular burial pit, above which was a destroyed construction of stone and roof tiles dislocated next to the grave, W-E orientation; disturbed **Skeletal information:** male, 30-45 years old. **Grave goods:** none; the grave had been opened and grave goods presumably removed **Dating**: archaeological: 380–410 CE (Phase II of necropolis); radiocarbon: 261-418 cal CE (1685±20 BP, PSUAMS-8725).

**Ancestry summary:** Balkans Iron Age-related.

- **Genetic Identifier:** I15545. **Grave Identifier:** G-25. **Grave type:** grave lined with river pebbles, preserved on both sides of the head, below the feet and along the right side of the skeleton, W-E orientation; destroyed by digging of medieval grave 6. **Skeletal information:** male, 20-25 years old. **Grave goods:** 32 possibly decorative iron rivets with pyramidal heads from shoes or belt; an iron buckle in the form of the Latin letter B near the feet. **Dating**: 380–410 CE (Phase II of necropolis); radiocarbon: 417-538 cal CE (1610±15 BP, PSUAMS-8556).

**Ancestry summary:** Balkans Iron Age+Central/Northern Europe+Pontic-Kazakh Steppe.

- **Genetic Identifier:** I15546. **Grave Identifier:** G-26. **Grave type:** grave with destroyed river pebble construction, W-E orientation; nails indicate a wooden coffin **Skeletal information:** male, 20-40 years old. **Grave goods:** to the left of the skull, a bronze consecration coin of Constantine I from 341–346 and two bronze coins of Constans, minted 341–348, which may be intrusive from late Roman Grave 44, which is beneath G-26; a bronze buckle in the region of the pelvis, an amphora-shaped bronze belt-tip in the region of the pelvis and a small, olive green glazed ceramic vessel with three handles next to the right foot. **Dating**: archaeological: vessel: third or 4^th^ century ^[77]^; 380–410 CE (Phase II of necropolis). **Ancestry summary:** Balkans Iron Age-related.
- **Genetic Identifier:** I15547. **Grave Identifier:** G-27A. **Grave type:** with river pebble construction, W-E orientation; destroyed. **Skeletal information:** male, 30-35 years old; disarticulated Also situated above late Roman grave 44. **Grave goods:** a bronze buckle with a rectangular plate and a circular frame, a bronze loop and theconical shank of a leaf-shaped iron arrowhead. The grave had been opened and grave goods presumably removed. **Dating**: archaeological: 380–410 CE (Phase II of necropolis).

**Ancestry summary:** Balkans Iron Age-related.

- **Genetic Identifier:** I15548. **Grave Identifier:** G-28. **Grave type:** with river pebble construction, W-E orientation; destroyed. **Skeletal information:** male, 30-40 years old. **Grave goods:** iron buckle in the shape of the Latin letter B on the right pelvic bone. **Dating**: archaeological: 380–410 CE (Phase II of necropolis).

**Ancestry summary:** Balkans Iron Age-related.

- **Genetic Identifier:** I15552. **Grave Identifier:** G-97. **Grave type:** deceased in a grave with stone construction (around the skull), W-E orientation. **Skeletal information:** male, c. 40 years old. **Grave goods:** a leaf-shaped iron arrowhead in the region of the right pelvic bone and an animal bone (sheep or goat’s pelvic bone) in the region of the chest. The grave had been opened and grave goods presumably removed. **Dating**: archaeological: 380–410 CE (Phase II of necropolis).

**Ancestry summary:** Balkans Iron Age+Central/Northern Europe.

- **Genetic Identifier:** I15556. **Grave Identifier:** G-123. **Grave type:** no visible burial container, W-E orientation; partially damaged by late Roman grave 102, above G-123. **Skeletal information:** male (based on physical anthropology), 45-65 years old; an iron trilobed arrowhead between the ribs on the right side of the chest cavity was a possible cause of death. **Grave goods:** a bronze buckle with an oval plate and an oval frame in the area of the pelvis. **Dating**: archaeological: 380–410 CE (Phase II of necropolis).

**Ancestry summary**: excluded from analysis dataset based on low SNP count.

- **Genetic Identifier:** I15554. **Grave Identifier:** G-100. **Grave type:** Mortared brick and stone construction, W-E orientation; destroyed **Skeletal information:** male, 40-45 years old. **Grave goods:** a fragment of a glass goblet and two fragments of a ceramic lamp. The grave had been opened and grave goods presumably removed. **Dating**: archaeological: 410–450 CE (Phase III of necropolis).

**Ancestry summary:** Balkans Iron Age-related.

- **Genetic Identifier:** I15555. **Grave Identifier:** G-108. **Grave type:** cist constructed with roof tiles sides and brick floor; disturbed , W-E orientation. **Skeletal information:** male**,** c. 20 years old. **Grave goods:** none; the grave had been opened and grave goods presumably removed. **Dating**: archaeological: 410–450 CE (Phase III of necropolis).

**Ancestry summary:** Balkans Iron Age-related.

**1.18 Sipar, Umag (Croatia)**

Contact person(s): Branka Milošević Zakić

**Geographic Information**: The archeological site of Sipar is located in the north-western part of Croatian Istria, about 4 kilometres north of Umag, on a small peninsula (about 0.5 ha) that connects the 80-meter-long spur with the mainland.

**Brief settlement history**: In the written documents, Sipar is first mentioned along with other Istrian coastal towns (*civitates*), as *Sapparis* and *Sipparis* by the Anonymus Cosmographer of Ravenna of the 7^th^ – 8^th^ centuries. The archaeological excavations that started in 2013 have been led by Branka Milošević Zakić and revealed several phases of development of the site. The archeological evidence shows occupation at Sipar between the 1^st^ century BCE and the end of the 9^th^ century CE. The earliest material finds show that Sipar was inhabited at least from the middle of the 1^st^ century BCE, possibly as a temporary Roman military camp located on the trade route leading from northern Italy to the eastern Adriatic coast; it seems to have been disaffected in favor of the mainland after the 1^st^ century CE. The eastern part of the peninsula connected to the mainland and was powerfully fortified, apparently in the 6^th^ century, when emperor Justinian’s reconquest of Italy and new continental migrations made the coastal strongholds of the eastern Adriatic essential to the Byzantine Empire’s military infrastructure. Small finds document continuous occupation of the peninsula from the 6^th^ through the 9^th^ centuries and are particularly numerous from the 7^th^ century; a layer of soot, ashes, and cracked stones appears across the entire inhabited zone and documents the sack of the fortress and settlement ca. 700; whether the destruction involved the Lombards, Avars and/or Slavs is at present unclear. Further finds, particularly from the first half of the 8^th^ century, indicate that the ruins were incorporated into new, poorer structures; finds become progressively rarer and a naval attack recorded in 876 probably marks the end. In the late Middle Ages, Venice constructed a castle at the eastern, sea end of the peninsula that was still visible in the 1920s.

**Summary of sampled materials**: Excavations in the area of the settlement between 2013 and 2021 identified a total of 16 graves: all were subadults. This remarkable circumstance is due to the fact that children were buried differently from adults, in or near domestic buildings: late antique graves were dug through the walkway stemming from the first phase of the settlement, while early medieval graves ^[80]^ were dug into the layer of rubble resulting from the destruction ca. 700 CE. The deviations from a west-east grave orientation generally reflect the constraints of the buildings within or near which the children were buried (Figure 24 in Milošević Zakić, 2022) ^[80]^.

**Sampled Graves:**

- **Genetic Identifier:** I26766. **Grave Identifier:** Grave 9, 2018. **Grave type:** semi-elaborated improvised construction in stone slabs, NW-SE orientation. **Skeletal information:** male, 1-3 months old (complete skeleton). **Grave goods:** a bone comb. **Dating**: stratigraphy: ca. 650-800 CE the child’s grave was dug in and therefore after the destruction layer dated ~700 CE, and presumably before the abandonment of the area after ca. 876 CE; typological similarity with Graves 5 and 6, whose individuals have been radiocarbon dated (radiocarbon results to be reported in a study in preparation appear not to contradict the archaeological dating). **Additional information**: the burial was found in Room 19; the bottom of the grave consisted of the collapse layer; the deceased child place was partially covered with unfinished stones and the burial was closed with a larger stone slab.

**Ancestry summary**: Balkans Iron Age+Anatolia Roman+Eastern Europe.

- **Genetic Identifier:** I26765. **Grave Identifier:** Grave 10, 2018. **Grave type:** semi-elaborated improvised construction of stone slabs, NW-SE orientation. **Skeletal information:** male, perinatal, **Grave goods:** none. **Dating:** stratigraphy: ca. 650-800 CE, the child’s grave was dug in and therefore after the destruction layer dated ~700 CE, and presumably before the abandonment of the area after ca. 876 CE; typological similarity with Graves 5 and 6, whose individuals have been radiocarbon dated (radiocarbon results to be reported in a study in preparation appear not to contradict the archaeological dating). **Additional information:** a complete skeleton of a perinate. The burial was found in the SW corner of Room 18; the bottom was the previous collapse layer and it was covered with unfinished stones.

**Ancestry summary**: Balkans Iron Age-related.

- **Genetic Identifier:** I26767. **Grave Identifier:** Grave 11, 2018. **Grave type:** regular construction in stone slab, N-S orientation. **Skeletal information:** male, perinate. **Grave goods:** none. **Dating**: stratigraphic context: 6^th^-7^th^ century CE; burial typology similar to Grave 4, radiocarbon dated (radiocarbon results to be reported in a study in preparation appear not to contradict the archaeological dating). **Additional information:** the grave chamber (cover slab, cladding and bottom) was constructed of thinner slate slabs laid in the shape of a chest; it was located in the layer of red soil.

**Ancestry summary:** Balkans Iron Age+Anatolia Roman+Eastern Europe.

**1.19 Trogir (Croatia)**

**Geographic Information:** Trogir is a on a small island in the narrow channel separating the Croatian mainland from the Adriatic island of Čiovo, athwart the ancient shipping route along the Dalmatian coast, 20 km west of Split.

**Brief settlement history**: Trogir (ancient *Tragurion/Tragurium*) in central Dalmatia, shows a history of habitation (at least) since the Early/Middle Bronze Age Period. From the beginning of the Iron Age and, especially in the pre-Hellenistic phase, the settlement was an important commercial center on the eastern Adriatic maritime trade route ^[81]^.During the last two centuries BCE, the city was closely related to the Adriatic Greek colony of *Issa* and integrated into the central Dalmatian commonwealth ^[81]^, which flourished as a Roman ally. With the loss of *Issa*’s domination during the civil war between Caesar and Pompey in 47 BC, *Tragurium* was soon integrated into Roman *Illyricum* under the provincial capital of *Salona* ^[82,83]^.The town’s Roman economy relied on trade, stone quarries and the villas which exploited the neighboring coastal plain. The end of Late Antiquity is marked by the withdrawal of the population into the fortified island city ^[83]^. In the early Middle Ages the city was among the towns of Dalmatia under Byzantine sovereignty but it passed into Croatian, Hungarian and, ultimately, Venetian control in the course of the Middle Ages ^[84]^. Ceramic evidence from the Roman and Late Roman periods attests the integration of Trogir into central Mediterranean shipping networks connected to North African and eastern Aegean production and trading centers well into the first half of the 6^th^ century^[81,83]^.

**Summary of sampled materials:** We sampled materials from 2 different necropolises: Dobrić and Dragulin.

**1.19.1 Dobrić necropolis**

Contact person(s): Maja Bilić, Lujana Paraman, Julianne M. Paige, Anna J. Osterholtz

**Geographic Information:** The site of Dobrić is viewed as a main cemetery of Roman *Tragurium*. Located on the mainland just opposite of the historic town, the area is marked by an intersection of ancient roads and pathways leading from the northern town gate to its coastal plain (*ager*), and further towards the hinterland and the Kaštela bay. The available data suggest that the necropolis was in use from the Early Roman to the end of the Late Antique Period with documented cremation urn burials between the 1^st^-2^nd^ centuries CE and different types of inhumation burials (pit, amphora, tile and cist graves, sarcophagi, vaulted tombs) between the 2^nd^-6^th^ centuries CE ^[85]^.

**Excavation History:** In 2011, during construction work on the sewage and water supply system of Kaštela-Trogir, a small segment of the cemetery was excavated at the beginning of Put Dragulina Street, just south of the modern police station. The rescue excavation, led by the firm Palisada Ltd., documented 28 graves in several layers, dating from the end of the 2^nd^ or 3^rd^ century CE probably to the 5^th^ or 6^th^ century CE ^[86]^. Buried individuals were mostly perinates and infants ^[87,88]^, found in re-used amphoras in all layers of the cemetery. Other documented graves were pit burials (18%) and tile burials (14%), recorded only in lower layers. Except for one tile burial of a child and one pit burial of a perinate, tile and pit burials featured adult individuals ^[87]^.

We sampled 7 graves from this excavation of the Dobrić cemetery:

- **Genetic Identifier:** I26709. **Grave Identifier:** Grave 8. **Grave type:** amphora burial of unknown type, unknown orientation. **Skeletal information:** female, 34-40 weeks old. **Grave goods:** none. **Dating**: stratigraphy: 4^th^-6^th^ centuries CE. **Additional information**: Grave 8 was found above grave 22, which was above grave 17. Grave 22 has an African amphora, possibly Africana 3A or 3B (or 2 C/D), dated to the 4th century.

**Ancestry summary:** Balkans Iron Age+Anatolia Roman+North Africa.

- **Genetic Identifier:** I26718. **Grave Identifier:** Grave 17. **Grave type:** possible grave architecture (undetermined), N-S orientation, head to the north, disturbed. **Skeletal information:** male, over 18 years old. **Grave goods:** near the legs (disturbed), a small jug, a plate, a lamp and three iron nails were found. **Dating**: stratigraphy: 2^nd^-4^th^ centuries CE. **Additional information:** The adult was laid on his back in a plain burial pit, with hands placed on the chest. Several larger stones and tile fragments found around the grave suggest the possibility of destroyed grave architecture. The skeleton was partly preserved and fragmented. Grave 17 was found underneath grave 22, which was underneath grave 8.

**Ancestry summary**: Balkans Iron Age+Anatolia Roman.

- **Genetic Identifier:** I26719. **Grave Identifier:** Grave 25. **Grave type:** deceased in grave with tile construction, W-E orientation. **Skeletal information:** female, over 18 years old. **Grave goods:** none. **Dating**: stratigraphy: 2^nd^-4^th^ century CE. **Additional information:** the skeleton is partly preserved. Grave 25 was underneath grave 26, which was found underneath grave 29.

**Ancestry summary:** Balkans Iron Age+Central/Northern Europe+Sarmatian-related.

- **Genetic Identifier:** I26714. **Grave Identifier:** Grave 26. **Grave type:** amphora burial (type: Africana 2D – late 3rd/4th c), E-W orientation, with head in the east. **Skeletal information:** female**,** 38-40 weeks old. **Grave goods:** none. **Dating**: amphora type (Africana 2D) , stratigraphy: 4^th^ c. CE. **Additional information**: The skeleton was well preserved. Grave 26 was found underneath grave 29, and was buried above grave 25.

**Ancestry summary**: Balkans Iron Age+Anatolia Roman.

- **Genetic Identifier:** I26717. **Grave Identifier:** Grave 13. **Grave type:** amphora burial (type Late Roman 1), E-W orientation, with head on the East. **Skeletal information:** sex unknown**,** 2 years old. **Grave goods:** none. **Dating**: amphora type, stratigraphy: 4th-6th centuries CE. **Ancestry summary**: excluded from analysis dataset based on low SNP count.
- **Genetic Identifier:** I26713. **Grave Identifier:** Grave 1. **Grave type:** tile burial, W-E orientation, with head on the West. **Skeletal information:** sex unknown**,** adult. **Grave goods:** none. **Dating**: stratigraphy: 2nd-5th centuries CE. **Additional information**: found below infant amphora burial grave 6.

**Ancestry summary**: excluded from analysis dataset based on low SNP count.

- **Genetic Identifier:** I26711. **Grave Identifier:** Grave 2. **Grave type:** amphora burial (type: Late Roman 4), W-E orientation, with head on the West. **Skeletal information:** sex unknown, 36-40 weeks old. **Grave goods:** none. **Dating**: amphora type, stratigraphy: 4^th^-6^th^ centuries CE.

**Ancestry summary**: excluded from analysis dataset based on low SNP count.

**1.19.2 Dragulin necropolis**

Contact person(s): Lujana Paraman, Anna J. Osterholtz, Julianne M. Paige

**Geographic Information:** The site of Dragulin is located in the western part of Trogir's Malo polje plain, about 400 m west of the main cemetery of Roman Tragurium in the Dobrić area.

**Excavation history:** Several cremation burials found in the 1960s and 1990s revealed the existence of an Early Roman cemetery in this area, next to the modern street of Put Dragulina which follows the route of one of the main Roman roads leading from Tragurium toward the coastal hinterland. It is still unclear whether this was a public cemetery or one connected to one of the unidentified *villae rusticae* in the western part of the Malo polje plain.

In 2016, a rescue excavation discovered a small segment of the cemetery after construction work on a private parking lot revealed several stone urns. The mechanical digging destroyed the upper layers of the cemetery, leaving untouched only the graves buried in the soil down to the bedrock. The rescue excavation led by the Trogir Town Museum documented 42 graves ^[89]^.

**Summary of sampled materials**: About 60% of graves were cremated individuals (mostly adults) buried in different containers: various ceramic pots and stone urns. The remaining 40% of graves were inhumations of perinates and infants ^[87,88,90]^ who were buried in small pits or in amphoras, with the exception of one burial in a miniature stone urn (grave 5). The finds and stratigraphy date ^[91]^ the cremated burials and most of the perinatal and infant burials from the beginning of the 1^st^ to the end of 2^nd^ century CE, with two amphora burials belonging to the 3^rd^ and 4^th^ centuries CE ^[89]^.

We sampled 6 graves, all dated to the 1^st^ and 2^nd^ centuries CE:

- **Genetic Identifier:** I26706. **Grave Identifier:** Grave 5. **Grave type:** urn burial, unknown orientation. **Skeletal information:** sex unknown, 9 months old. **Grave goods:** 5 glass *unguentaria* (1 burnt), 2 ceramic *unguentaria*, 2 ceramic cups. **Dating**: stratigraphy and grave goods: first half of the 1st century CE. **Additional information**: The infant was laid in a miniature stone urn, with four glass *unguentaria* placed on the infant’s body. The urn was set in a round burial pit with ash placed around the urn, and one burnt glass *unguentarium*, two ceramic *unguentaria* and two ceramic cups placed on top of the ash.

**Ancestry summary:** excluded from analysis dataset based on low SNP count.

- **Genetic Identifier:** I26708 and I26702. **Grave Identifier:** Grave 14 (1) and (2). **Grave type:** no visible burial container, S-N orientation with heads to the South; double burial. **Skeletal information:** (1): female, about 2 months old; (2) male, about 2 months old. **Grave goods:** none. **Dating**: stratigraphy: end of 1^st^–2^nd^ centuries CE. **Additional information**: shallow round pit burial with two infants, genetically siblings. They were laid on the side (infant 1 on the right, infant 2 on the left side), facing each other (infant 1 facing east, infant 2 facing west). The skeletons were partly damaged by the construction work, especially infant 2 (poorly preserved and fragmentary). Grave 14 was found above grave 31.

**Ancestry summary**: (1): Balkans Iron Age-related; (2): Balkans Iron Age-related.

- **Genetic Identifier:** I26707. **Grave Identifier:** Grave 21. **Grave type:** amphora burial, E-W orientation with the head in the east. **Skeletal information:** female, c. 1 year old. **Grave goods:** ceramic *unguentarium* (possibly a grave furnishing, disturbed) found SE of the amphora (type unknown) and a ceramic cup found *in situ,* placed by the NW side of the amphora. **Dating**: artifacts, stratigraphy: end of 1^st^–2^nd^ centuries CE. **Additional information:** the infant was buried in an amphora, laid on the right side, facing north. The amphora was set in an oval burial pit. The grave was partly damaged by construction work, and the skeleton was heavily fragmented.

**Ancestry summary**: Anatolia Roman-related.

- **Genetic Identifier:** I26704. **Grave Identifier:** Grave 25. **Grave type:** amphora burial, E-W orientation with the head in the east. **Skeletal information:** female, 2-3 months old. **Grave goods:** none. **Dating**: stratigraphy: 1^st^–2^nd^ centuries CE. **Additional information**: The infant was buried in an amphora (type unknown), laid on the left side, facing south. The amphora was set in an oval burial pit. Partly damaged by the construction work, good preservation of the skeleton. Grave 25 was found above grave 30.

**Ancestry summary**: Balkans Iron Age+Anatolia Roman.

- **Genetic Identifier:** I26703. **Grave Identifier:** Grave 28. **Grave type:** no visible burial container, W-E orientation with the head on the West. **Skeletal information:** female, c. 40 weeks old. **Grave goods:** none. **Dating**: stratigraphy: 1^st^–2^nd^ centuries CE. **Additional information**: The perinate was set in a shallow burial pit, laid on the back, head facing north. The grave was partly damaged by the construction work, but the skeleton was well preserved.

**Ancestry summary**: Balkans Iron Age-related.

- **Genetic Identifier:** I26705. **Grave Identifier:** Grave 31. **Grave type:** no visible burial container, W-E orientation, head in the north. **Skeletal information:** female, 38–40 weeks old. **Grave goods:** glass *unguentarium* placed east of the perinate's neck, with the vessel lip placed on the perinate's mandible. **Dating**: artifacts, stratigraphy: second half of the 1^st^–2^nd^ centuries CE. **Additional information**: The perinate was set in a shallow oval burial pit, laid on the left side, facing east. The skeleton was well preserved. Grave 31 was found underneath grave 14.

**Ancestry summary**: Balkans Iron Age-related.

**1.20 *Viminacium* (Serbia)**

Contact person(s): Ilija Mikić

**Geographic Information**: The site is located 12 km from the modern town of Kostolac in eastern Serbia, at the confluence of the Mlava river and the Danube, and lies on the Roman road referred to by scholars as the “Military Road” (*Heerstrasse*, *Via militaris*).

**Brief settlement history**: *Viminacium* was the capital of Upper Moesia, a Roman province largely in the territory of present-day Serbia. The military significance of *Viminacium* lay in its strategic position on the Danube River; it was the permanent camp for the Roman legion *Legio VII Claudia pia Fidelis*, and for a time the *Legio IV Flavia* as well. This town saw a period of prosperity during the 2^nd^ century and developed into the largest settlement in Upper Moesia. This development was in part due to *Viminacium’s* location at the mouth of the Mlava River, which made it a port for the export of the Mlava Valley’s agricultural and mining products along the Danube. The settlement achieved the status of *municipium*, probably during Emperor Hadrian’s rule. Under Emperor Gordian III (reigned 238-244 CE), the town received the status of colony, with the right to mint coins. *Viminacium* acted as a crossroads which connected the north of the Balkan peninsula with the Roman-controlled west and south. The Pannonia road also passed through the town, crossing the Danube and ending at the Black Sea ^[92,93]^. In the Middle Ages, the Byzantine fortress and bishopric of Braničevo, 30 km to the east, was the area’s main settlement and focal point of conflict between Byzantium, Serbia and Hungary ^[94]^.

**Excavation history:** Open-air mining of brown coal deposits have played an important role in uncovering the archaeological deposits around *Viminacium*. Multiple necropolises have been excavated in the area south of the Roman town beginning in the 19^th^ century. Physical anthropological analysis of the recovered individuals at *Viminacium’s* necropolises has pointed to morphologically diverse profiles ^[40,95,96]^. In some cases, grave orientation could be related to religious beliefs; pagan or Christian beliefs coexisted in *Viminacium*. A map of the *Viminacium* excavations can be found in Figure 2 below.

**Summary of sampled materials**: In what follows, we describe the archaeological context and inventory of the graves selected for ancient DNA analysis. Individuals were chosen from four necropolises within the very large burial zone south of the town (“southern cemeteries”) (Figure 2 below**)** based on archaeological context, dating and skeletal preservation. A catalogue of ceramic finds from *Viminacium* necropolises is currently in press and our contributors have made frequent use of its findings ^[97]^.

**
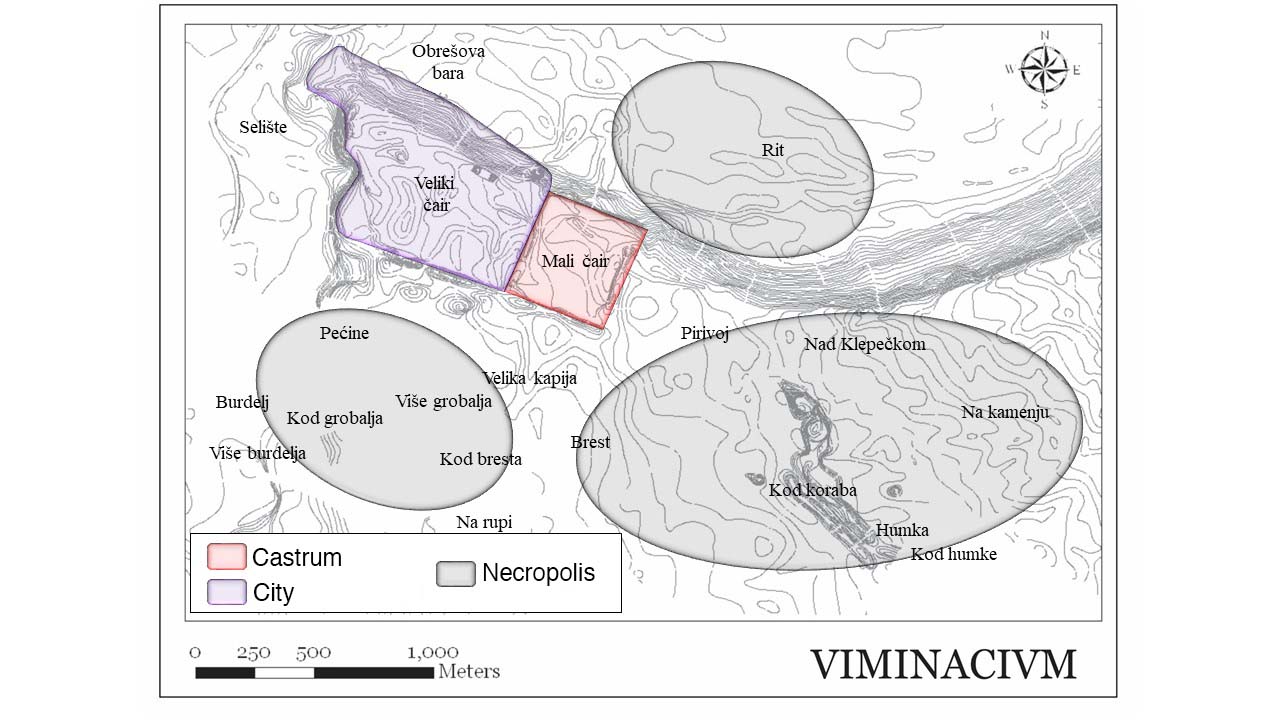
**

**Figure 2.** **Map of *Viminacium* archaeological site.**

**1.20.1 Pećine necropolises**

The necropolis of Pećine is the largest at *Viminacium*, and archaeological research is still ongoing ^[98]^. So far, over 6,000 graves from prehistoric periods (including a Celtic-associated cemetery) to the Middle Ages have been identified. For this project, we selected two sets of individuals, one from the large burial place of Pećine, with graves dated from the 1^st^ to 4^th^ century CE, and another at the site known as “Kod Groblja”, whose burials date to the Middle Ages ^[98–101]^.

**2.22.1.1 Kod Groblja (“At the Cemetery”)**

**Geographic Information:** This group of graves sits about 20 m east of the modern village cemetery ^[102]^, hence its name. They are located on a circular ridge that stands out from the surrounding plains, 750 m south of the southern rampart of the former Roman military camp of Viminacium, and about 800 m from the bank of the Danubian tributary of the Mlava River. In the Middle Ages, it was about 25 km west of the Byzantine fortress and bishopric of Braničevo on the contested frontier of the kingdoms of Serbia and Hungary.

**Excavation History:** The necropolis was completely excavated in 1982 and contained 27 graves dated to the 12th-13th centuries situated around the ruin of a late antique triapsidal structure ^[103]^, for which associated coins suggest a 4th-century date. Graves were scattered on the southern and eastern sides of the structure, and one grave was buried over the remains of its eastern wall. The southern part of the cemetery lies among the remains of secular architecture from the 3rd century. All the graves were buried in a layer of rubble from the aforementioned structure. In some cases, the skeletal remains were poorly preserved. The archaeological material from this necropolis consists of grave goods in the form of buttons, rings, earrings, coins.

We sampled 1 medieval grave:

- **Genetic Identifier**: I32303**. Grave Identifier**: grave 17 (G4-1883), T. I/J 1. **Grave type**: no visible burial container, W-E orientation with a deviation of 4° at the western end towards the north. **Skeletal information**: male, over 45 years old. **Grave goods**: Silver penny identified as issued by a king of Hungary and Croatia between Andrew II (1205-1235) and Ladislav IV (1272-1290). Obv: In a double circle of lines, a facing crowned head between two towers. Rev: crowned panther head (possibly a griffin) facing left. **Dating**: 13th century based on the coin and the general chronology of the necropolis. **Additional information:** the grave is located at an absolute elevation of 77.32 m. The deceased was buried in a gravel layer with brown earth, laid on his back. The arms were fully bent at the elbows with the fists on the shoulders, the left hand on the left shoulder, and the right hand on the right shoulder. The length of the skeleton is 1.70 m. The skeleton presented an injury on the left elbow and hand.

**Ancestry summary**: excluded from analysis dataset based on low SNP count.

**1.20.1.2 Pećine**

**Geographic Information**: south of the Roman town.

**Excavation History:** Although the burials here have been known since 1882, excavations began in 1973 and intensified between 1977 and 1988 in anticipation of the construction of a thermal power plant. So far some 6000 inhumations and 1150 cremation burials have been excavated dating from the 1^st^ to the 4^th^ century. Grave goods are generally modest and may indicate relatively low or middling economic status. Graves G-5757 and G-5762 (not sampled for ancient DNA) can be dated by the coins reportedly found in them ^[104]^ to in or after the 330s CE. Unfortunately the catalogue of coins from these burials reports many of the graves under a different identification system, making it impossible in several cases to offer further details ^[104]^. West-east and east-west burial orientations have been taken by the excavators to suggest that some individuals might have been Christians. Such a chronology would align with two radiocarbon dates from graves G-5665 (258-413 cal CE (1700±20 BP, PSUAMS-8554)); and G-5736 (246-365 cal CE (1745±15 BP, PSUAMS-8591)) from the same necropolis that stem from the same or a similar archaeological context. Another radiocarbon date from G-2771 (I15527; 70-208 cal CE (1910±20 BP, PSUAMS-8553)) yielded an earlier date within the 2^nd^ century CE (Data S2, Tables 1 and 9, including for the isotope values that do not appear to argue for a significant fish consumption that could result in freshwater reservoir effect ^[105,106]^). Burial practices are typical of the Roman period at *Viminacium* (graves with no construction, deceased in wooden coffins or graves constructed with bricks, including some stamped by the producing legion).

We sampled 12 graves from the Roman period:

- **Genetic Identifier:** I15531. **Grave Identifier:** G-5665. **Grave type:** no visible burial container, S-N orientation. **Skeletal information:** male, undetermined age. **Grave goods:** none. **Dating:** stratigraphy: 4^th^ century; radiocarbon: 258-413 cal CE (1700±20133BP, PSUAMS-8554).

**Ancestry summary:** Balkans Iron Age+Central/Northern Europe+Pontic-Kazakh Steppe.

- **Genetic Identifier:** I15532. **Grave Identifier:** G-5703. **Grave type:** no visible burial container, N-S orientation. **Skeletal information:** male, 35-40 years old. **Grave goods:** a bronze belt buckle was found on the inside of the left femur, and a narrow bronze piece of the belt in the shape of a stylised amphora was found on the inside of the right forearm. **Dating:** buckle: 4^th^ century CE ^[107]^.

**Ancestry summary:** Anatolia Roman-related.

- **Genetic Identifier:** I15533. **Grave Identifier:** G-5736. **Grave type:** no visible burial container, E-W orientation, the deceased was laid over the left leg of the skeleton of grave G-5735. **Skeletal information:** male, age undetermined. **Grave goods:** none. **Dating**: radiocarbon date 246-365 cal CE (1745±15 BP, PSUAMS-8591); stratigraphy: 3^rd^ century based on pottery fragments from the same stratigraphic layer.

**Ancestry summary:** Balkans Iron Age+Pontic-Kazakh Steppe.

- **Genetic Identifier:** I15527. **Grave Identifier** G-2771. **Grave type:** no visible burial container, W-E orientation. **Skeletal information:** male, adult under 45 years old. **Grave goods:** none present. **Dating:** stratigraphy: 2^nd^ century; radiocarbon: 70-208 cal CE (1910±20 BP, PSUAMS-8553; similar isotope values to I15527 discussed above, Excavation History.

**Ancestry summary:** Balkans Iron Age+Central/Northern Europe.

- **Genetic Identifier:** I15530. **Grave Identifier:** G-4661. **Grave type:** no visible burial container, burial in prehistoric pit, S-N orientation. **Skeletal information:** c. 23 years old**,** undetermined sex. **Grave goods:** none. **Dating**: 2^nd^-century stratigraphy.

**Ancestry summary:** excluded from analysis dataset based on low SNP count.

- **Genetic Identifier:** I15536. **Grave Identifier:** G-5924. **Grave type:** no visible burial container, NW-SE orientation. **Skeletal information:** undetermined sex and age (probably adult, but very poor preservation). **Grave goods:** none. **Dating**: 1-400 CE, based on the cemetery lifespan.

**Ancestry summary:** excluded from analysis dataset based on low SNP count.

- **Genetic Identifier:** I15535. **Grave Identifier:** G-5769. **Grave type:** two bricks over the skull imply a roof-like structure, NW-SE orientation. **Skeletal information:** female, undetermined age. **Grave goods:** a bronze coin reported as fourth-century by excavators ^[104]^ found next to the right side of the femur and 2 bricks. **Dating**: radiocarbon: 255-409 cal CE (1715±20 BP, PSUAMS-9564).

**Ancestry summary:** Balkans Iron Age+Pontic-Kazakh Steppe.

**Genetic Identifier:** I32304. **Grave Identifier:** G-139. **Grave type:** no visible burial container, only skulls found. **Skeletal information:** male (based on physical anthropology), 30-40 years old. **Grave goods:** none. **Dating**: surrounding graves date from the 2^nd^ and 3^rd^ centuries. **Additional information:** In the grave, two skulls were found leaning against each other and without the remains of postcranial skeletons.

**Ancestry summary:** North African.

- **Genetic Identifier:** I15534. **Grave Identifier:** G-5752. **Grave type:** metal nails indicate a wooden coffin, E-W orientation. **Skeletal information:** female, over 40 years old. **Grave goods:** a bronze coin reportedly dated to the 330s on the left side of the chest but which cannot be located under this Grave Identifier in the coin catalogue ^[104]^. **Dating**: coin and ceramic in the same stratigraphic layer: 4^th^ century; radiocarbon: 237-348 cal CE (1765±20 BP, PSUAMS-9563).

**Ancestry summary:** Balkans Iron Age-related.

- **Genetic Identifier:** I15529. **Grave Identifier:** G-3082. **Grave type:** metal nails indicate a wooden coffin, SE-NW orientation. **Skeletal information:** male, under 30 years old. **Grave goods:** fragmented red painted oil-lamp. The concave discus is separated from the shoulder part with two shallow gutters. It bears an image of a rosette. The handle is formed together with the upper half of the oil-lamp, and has been identified as the type Viminacium VIII – Variant R – Loeschcke VIII which includes 380 examples, dated from Titus to Marcus Aurelius ^[108]^. **Dating**: lamp and ceramics: 2^nd^ century CE.

**Ancestry summary:** Anatolia Roman-related.

- **Genetic Identifier:** I15528. **Grave Identifier:** G-3053. **Grave type:** metal nails indicate a wooden coffin, NE-SW orientation. **Skeletal information:** female, under 30 years old. **Grave goods:** three ceramic vessels: a censer, a pot and a jug. A red-painted ceramic lamp with illegible seal. The sunken discus bears an image of a tragic mask. The lamp type is: Viminacium X A; Fischbach A; Loeschcke X A; Iványi XVII; Ponsich V A; Dressel 5; Dressel – Lamboglia 5 c. ^[108]^. Next to the right shoulder, a bronze coin dated to Trajan (98-117) ^[104]^. **Dating**: coin: during or after 98-117 CE; vessels ^[109–112]^: censer dated to the early 2^nd^ century, pot dated century. 150-300, jug dated c. 150-250; lamp: 2^nd^ century ^[108,113,114]^; radiocarbon: 80-215 cal CE (1895±20 BP, PSUAMS-9562). Probable burial date: second or early third century.

**Ancestry summary:** Balkans Iron Age+Anatolia Roman.

**1.20.2 Pirivoj necropolis**

**Geographic Information:** The Pirivoj necropolis is located about 700 m east of *Viminacium* and, among other features, contains a mausoleum complex (measuring 20 x 20 m), constructed with stone blocks and ashlars decorated with columns. A tomb was found in the center of this construction (*bustum* burial type), which are generally very rare at Viminacium. The buried individual was cremated, and it appears to have been a person of great distinction in the Roman aristocracy ^[115–118]^.

**Excavation History:** The Pirivoj necropolis is named after the local name for the place east of the *Viminacium* legionary camp (Figure 2 above). Probe excavations were carried out in this area in 1997, and rescue excavations during 2003-2007, 2011 and 2013 resulted in the discovery of the eastern necropolis of *Viminacium*. A total of 413 inhumation graves and 68 cremated graves were uncovered. The necropolis was formed along the road that led from the eastern gate of the castrum to *Pincum* (Veliko Gradište), and which was archaeologically confirmed in three places at the Pirivoj site ^[119]^. In addition to the sepulchral elements characteristic of the site, a brick kiln was also discovered. The most recent work at the Pirivoj site was carried out on the area intended for the reconstruction and presentation of the antique tomb-*memoriae* discovered during the rescue campaign at the Pećine site, which was planned for relocation due to the threat of construction work on the new block of the Kostolac B thermal power plant. Two objects, apparently of funerary character, were investigated, as well as 36 graves containing 28 inhumations and eight cremations ^[120]^.

**Summary of sampled materials:** About 450 inhumed skeletons have been excavated and anthropologically analyzed. Archaeological finds suggest that the people sampled for this project are individuals of a middle/low class status who can be dated archaeologically from the 2^nd^ to the 4^th^ century, confirmed with radiocarbon dates of I15499/G-103 (80-215 cal CE (1895±20 BP, PSUAMS-8552)), I15517/G-402 (124-228 cal CE (1870±20 BP, PSUAMS-8590)) and I15492/G-47A (241-362 cal CE (1755±20 BP, PSUAMS-9557)) (Data S2, Tables 1 and 9).

Sampled graves:

- **Genetic Identifier:** I15486. **Grave Identifier:** G-12. **Grave type:** Tomb built with stone squares; disturbed (grave goods removed) **Skeletal information:** female, undetermined age. **Grave goods:** fragments of a glass vessel and of a ceramic vessel. **Dating**: stratigraphy and bowl: 3^rd^ century.

**Ancestry summary:** Balkans Iron Age+Anatolia Roman.

- **Genetic Identifier:** I15495. **Grave Identifier:** G-61. **Grave type:** no visible burial container, W-E orientation**. Skeletal information:** male, 50-60 years old. **Grave goods:** a bronze coin dating to the second half of the 1^st^ century, but possibly intrusive and belonging to a cremation grave that was intersected by G-61. **Dating**: stratigraphy: 2^nd^ century.

**Ancestry summary:** Balkans Iron Age-related.

- **Genetic Identifier:** I15511. **Grave Identifier:** G-322. **Grave type:** no visible burial container, S-N orientation**. Skeletal information:** male, undetermined age. **Grave goods:** pottery fragments from a beaker. **Dating**: stratigraphy: 3^rd^ century; ceramics: from the middle of the 2^nd^ century to the middle of 3^rd^ century.

**Ancestry summary:** excluded from individual ancestry analysis based on low SNP count.

- **Genetic Identifier:** I15510. **Grave Identifier:** G-314. **Grave type:** no visible burial container, W-E orientation**. Skeletal information:** male, undetermined age. **Grave goods:** three iron rings and a glass vessel. **Dating**: stratigraphy: 3^rd^ century.

**Ancestry summary:** Balkans Iron Age-related.

- **Genetic Identifier:** I15515. **Grave Identifier:** G-357. **Grave type:** no visible burial container, NE-SW orientation**. Skeletal information:** male, child. **Grave goods:** none. **Dating**: stratigraphy: 2^nd^ century.

**Ancestry summary:** Balkans Iron Age-related.

- **Genetic Identifier:** I15514. **Grave Identifier:** G-353. **Grave type:** no visible burial container, W-E orientation. **Skeletal information:** female, 40-60 years old. **Grave goods:** bronze coin (middle of the 3^rd^ century) on the chest. **Dating**: coin: after ca. 250 CE; stratigraphy: 3^rd^ century.

**Ancestry summary:** Balkans Iron Age-related.

- **Genetic Identifier:** I15498. **Grave Identifier:** G-101. **Grave type:** metal nails indicate a wooden coffin, W-E orientation**. Skeletal information:** male**,** 18-20 years old. **Grave goods:** red-painted oil lamp; Roman bronze coin: double as. **Dating**: stratigraphy: stratigraphically coincides with I15499/G-103; coin: 1^st^-2^nd^ century CE.

**Ancestry summary:** excluded from analysis dataset based on low SNP count.

- **Genetic Identifier:** I15501. **Grave Identifier:** G-104. **Grave type:** metal nails indicate a wooden coffin, NW-SE orientation. **Skeletal information:** male (based on physical anthropology)**,** 25-30 years old. **Grave goods:** none**. Dating**: uncertain: coins in surrounding and nearby graves dated from ca. 50 to 350 CE.

**Ancestry summary:** excluded from analysis dataset based on low SNP count.

- **Genetic Identifier:** I15499. **Grave Identifier:** G-103. **Grave type:** metal nails indicate a wooden coffin, N-S orientation (Figure 3 below). **Skeletal information:** male, 15-18 years old. **Grave goods:** oil lamp with spread-winged eagle on disc and palmette handle with rounded nozzle (cf. *Viminacium* type XI) ^[108]^ by right leg (Figure 2C in the main text), bronze coin, double as in the mouth, gray ceramic pot, two fragments of a glass vessel, fragments of various ceramic vessels. **Dating**: coin: 1^st^-2^nd^ century; pot: from the 2^nd^ century to the end of 3^rd^ century; lamp: ca. 120-280; radiocarbon: 80-215 cal CE (1895±20 BP, PSUAMS-8552); all of which places individual I15499 in the second or third century CE. **Additional Information:** this young man, whose ancestry probably lay in East Africa, also displayed high δ^15^N and more positive δ^13^C values that indicate the likely consumption of marine protein sources during his childhood ^[121]^ (Data S2, Table 9). The isotopic analysis was performed on the root of his lower left second premolar, which forms at the age of 12 ± 2.5 years ^[122]^.While the eagle would have been associated with Jupiter-Zeus in the Roman world ^[123]^, it was also singularly prominent in the iconography of Nabataean culture ^[124]^, which perhaps hints at a connection of this young man and/or the survivors who buried him with the Nabataean trading routes linking the Roman Empire to the Indian Ocean.

**Ancestry summary:** East African related ancestry.

- **Genetic Identifier:** I15502. **Grave Identifier:** G-105. **Grave type:** metal nails indicate a wooden coffin, E-W orientation. **Skeletal information:** male, 15-25 years old. **Grave goods:** none. **Dating**: radiocarbon: 215-326 cal CE (1800±20 BP, PSUAMS-13191).

**Ancestry summary:** Anatolia Roman-related.

- **Genetic Identifier:** I15517. **Grave Identifier:** G-402. **Grave type:** metal nails indicate a wooden coffin, NW-SE orientation. **Skeletal information:** male, undetermined age. **Grave goods:** none. **Dating**: radiocarbon: 124-228 cal CE (1870±20 BP, PSUAMS-8590).

**Ancestry summary:** Balkans Iron Age-related.

- **Genetic Identifier:** I15490 and I15491. **Grave Identifier:** G-36 (A and B). **Grave type:** brick construction, W-E orientation, dislocated and fragmented bones of several deceased, only one fully articulated. **Skeletal information:** two individuals were sampled, both male, A: over 50 years old; B: 4-7 years old. **Grave goods:** a mirror, bronze probe and ring. **Dating**: bronze objects: 3^rd^ century. **Additional information:** The deceased in this double burial were buried simultaneously.

**Ancestry summary**: both Balkans Iron Age-related.

- **Genetic Identifier:** I15492, I15493, and I15494. **Grave Identifier:** G-47 (A, B and D). **Grave type:** brick construction, W-E orientation, McCormick Type I ^[57]^, simultaneous, burial of at least three, possibly five individuals. **Skeletal information:** A: male, 35-45 years old; B: 4-7 years old, undetermined sex; D: female, 40-50 years old. **Grave goods:** glazed beaker and pottery fragments. **Dating**: beaker and ceramics: 4^th^ century; radiocarbon (individual A): 241-362 cal CE (1755±20 BP, PSUAMS-9557).

**Ancestry summary:** A: Balkan Iron Age-related; B: excluded from analysis dataset based on low SNP count; D: Balkans Iron Age-related.

- **Genetic Identifier:** I15509. **Grave Identifier:** G-288. **Grave type:** brick construction, N-S orientation. **Skeletal information:** female, 16-18 years old. **Grave goods:** bronze cylindrical box, silver ring and necklace composed of a jet pendant in the form of an anthropomorphic being and 68 glass, stone, amber and jet beads ^[125]^. **Dating**: stratigraphy, box, ring and beads: 3^rd^ century ^[125]^.

**Ancestry summary:** Balkans Iron Age related.

- **Genetic Identifier:** I15516. **Grave Identifier:** G-361. **Grave type:** metal nails indicate a wooden coffin, N-S orientation. **Skeletal information:** male, 25-30 years old. **Grave goods:** none. **Dating**: stratigraphy: 2^nd^ century; radiocarbon: 127-233 cal CE (1860±20 BP, PSUAMS-13192).

**Ancestry summary:** Balkans Iron Age+Central/Northern Europe.

- **Genetic Identifier:** I15512. **Grave Identifier:** G-341. **Grave type:** unknown. **Skeletal information:** male, adult. **Grave goods:** a ceramic pot. **Dating**: ceramic: 2^nd^-3^rd^ century.

**Ancestry summary:** excluded from analysis dataset based on low SNP count.


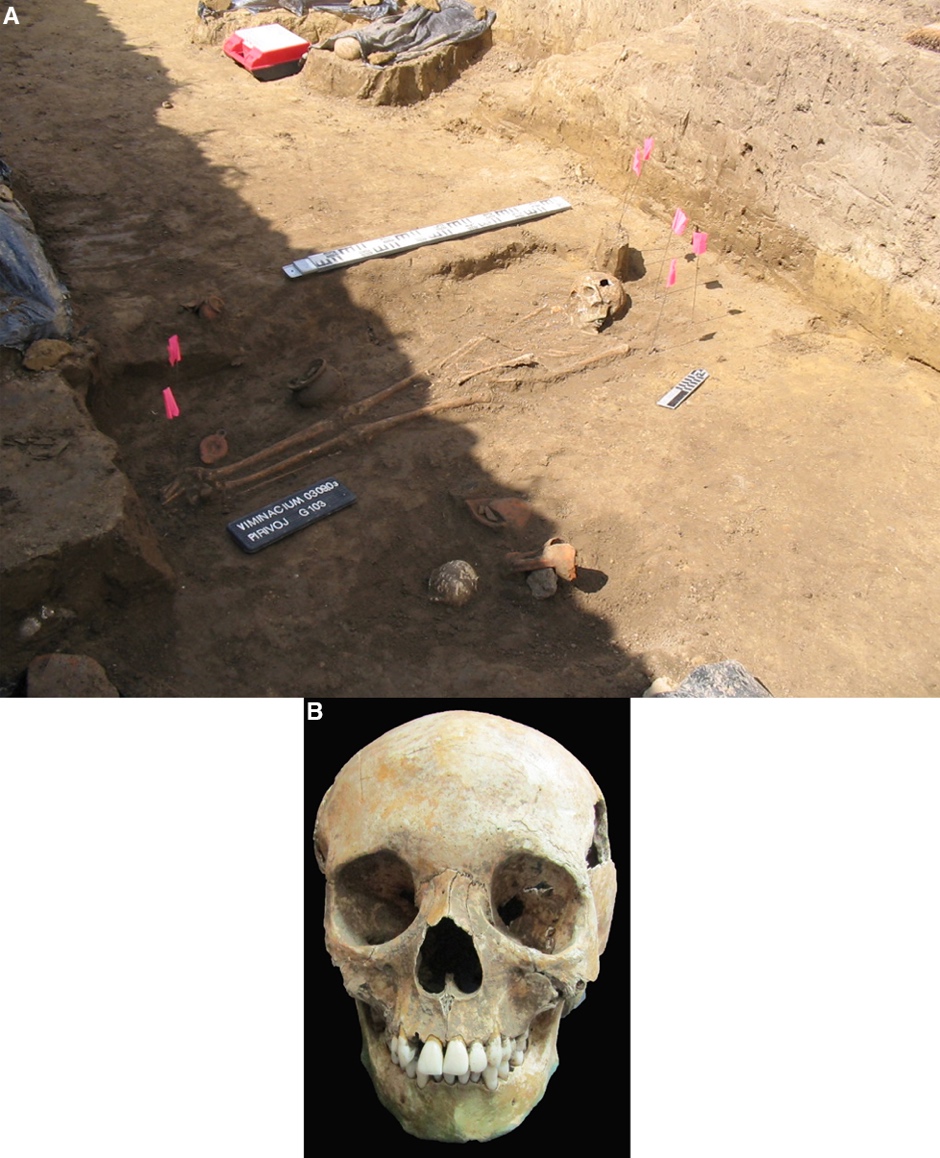


**Figure 3**. **Grave G-103 (I15499) from the Pirivoj necropolis, *Viminacium***. (A) Grave G-103 during excavation. (B) Frontal view of G-103’s skull.

**1.20.3 Rit necropolis**

**Geographic Information:** northeast of the Roman town.

**Excavation History:** The Rit necropolis was excavated as part of a settlement that contained Roman villas and other non-religious buildings.

**Summary of sampled materials:** A total of 150 skeletons, all inhumation burials, were recorded. In addition to burials that are typical of the Roman population at *Viminacium*, this necropolis also features burials such as two sarcophagi (G30 and G148) (Figure 4 below) each containing a male-female pair and rich grave goods that can be dated between the 1^st^ and the 3^rd^ century CE. Three out of four individuals found in these two sarcophagi yielded Near Eastern ancestry (see section 4 below).


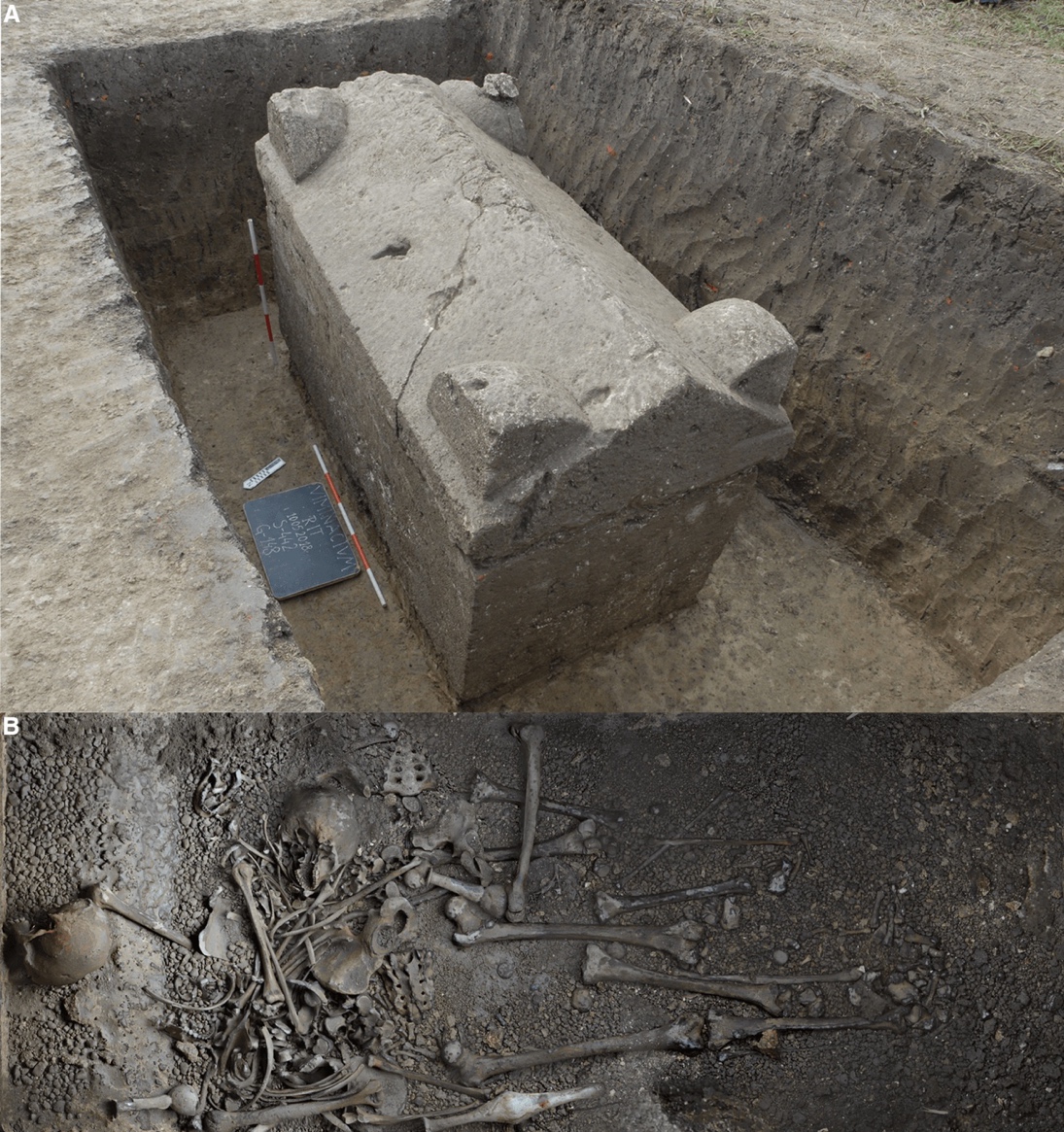


**Figure 4**. **Grave 148 from the Rit necropolis**. (A) Sarcophagus of grave 148. (B) Human remains of two individuals (I15507 and I15508) and grave goods found at grave 148.

Sampled graves:

- **Genetic Identifier:** I15497. **Grave Identifier:** G-81. **Grave type:** no visible burial container, W-E orientation **Skeletal information:** male, 10-11 years old. **Grave goods:** Ceramic pot (*Viminacium* type II/50) with flared rim, conical receptacle and flat bottom. Part of the rim and the door were damaged. The clay is a dark gray color of firing, poor texture. **Dating**: ceramics: from the end of 1^st^ to the beginning of 3^rd^ century; stratigraphy: 2^nd^ –3^rd^ century.

**Ancestry summary:** excluded from analysis dataset based on low SNP count.

- **Genetic Identifier:** I15504. **Grave Identifier:** G-112. **Grave type:** metal nails indicate a wooden coffin, S-N orientation. **Skeletal information:** male, over 40 years old. **Grave goods:** three fragmented jugs on the right side of the skull and a bronze coin (Antoninus Pius, 138-161 CE) under the lower jaw. **Dating**: ceramics, stratigraphy, numismatics: 3^rd^ century.

**Ancestry summary:** Balkans Iron Age-related.

- **Genetic Identifier:** I15487. **Grave Identifier:** G-23. **Grave type:** metal nails indicate a wooden coffin, W-E orientation. **Skeletal information:** female, 20-25 years old. **Grave goods:** 3^rd^ century bronze coin, bronze ring, beads and beaker pottery fragments ^[112]^. **Dating**: artifacts, stratigraphy: 3^rd^ century.

**Ancestry summary:** Balkans Iron Age-related.

- **Genetic Identifier:** I15506. **Grave Identifier:** G-147. **Grave type:** metal nails indicate a wooden coffin, W-E orientation. **Skeletal information:** sex unknown**,** 7-13 years old. **Grave goods:** bronze coin (Antoninus Pius, 138-161 CE). **Dating**: numismatics, stratigraphy: 2^nd^-3^rd^ century.

**Ancestry summary:** excluded from analysis dataset based on low SNP count.

- **Genetic Identifier:** I15485. **Grave Identifier:** G-11. **Grave type:** deceased in grave with brick construction, W-E orientation. **Skeletal information:** undetermined sex and age. **Grave goods:** none. **Dating**: stratigraphy: 2^nd^ - 3^rd^ century.

**Ancestry summary:** excluded from analysis dataset based on low SNP count.

- **Genetic Identifier:** I15519. **Grave Identifier:** G-84B. **Grave type:** deceased in grave with brick construction, W-E orientation. **Skeletal information:** female, 21-24 years old. **Grave goods:** none. **Dating**: stratigraphy: 2^nd^-3^rd^ century. Additional information: The grave contained another adult individual (G-84A).

**Ancestry summary:** excluded from analysis dataset based on low SNP count.

- **Genetic Identifier:** I15496. **Grave Identifier:** G-65. **Grave type:** deceased in grave with brick construction, W-E orientation. **Skeletal information:** female, 30-40 years old. **Grave goods:** bone hairpin and pottery sherds. **Dating**: ceramics, stratigraphy: 3^rd^ century.

**Ancestry summary:** excluded from individual ancestry analysis based on low SNP count.

- **Genetic Identifier:** I15505. **Grave Identifier:** G-123. **Grave type:** deceased in grave with brick construction, W-E orientation. **Skeletal information:** male, 4–8 years old. **Grave goods:** 2^nd^ century bronze coin. **Dating:** numismatics, stratigraphy: 2^nd^ - 3^rd^ century.

**Ancestry summary:** excluded from analysis dataset based on low SNP count.

- **Genetic Identifier:** I15500. **Grave Identifier:** G-103. **Grave type:** deceased in grave with brick construction, W-E orientation. **Skeletal information:** female under 45 years old. **Grave goods:** bronze ring, bone hairpin, iron knife and lead mirror. **Dating:** stratigraphy and items: 3^rd^ century; radiocarbon: 129-247 cal CE (1835±20 BP, PSUAMS-8560).

**Ancestry summary:** Balkans Iron Age-related.

- **Genetic Identifier:** I15488 and I15489. **Grave Identifier:** G-30. **Grave type:** sarcophagus, W-E orientation. **Skeletal information:** female, over 40 years old; male, 25-35 years old. **Grave goods:** Fragments of bricks. Opened and objects presumably removed in Roman times. **Dating:** stratigraphy: 3^rd^ century.

**Ancestry summary:** both individuals Anatolia Roman-related.

- **Genetic Identifier:** I15507 and I15508. **Grave Identifier:** G-148A and B. **Grave type:** sarcophagus, W-E orientation. **Skeletal information:** A: male, 30- 40 years old; B: female, 20-30 years old. **Grave goods:** 4 bronze coins (*Viminacium* mint), a glass unguentarium with slightly damaged thickened rim, a long cylindrical neck, a biconical recipient, and a rounded base, 2 glass balsamaria, a jet hairpin with a spindle-shaped circle-sectioned shank and a globular head, a bone hairpin, 2 golden earrings with “S”-shaped hooks used for fastening and with flower-shaped heads with six oval petals and a circle in the middle, a golden ring with a circular cross-section, angled highlighted shoulders and a carved retrograde inscription SIMP on the flat head, a silver circular, convex mirror with a horizontal handle applied to the back made of two thick silver wires braided to form a Herculean knot motif, a silver T-shaped brooch closed with a hinge mechanism ^[126]^, 151 gold beads, 126 pearl beads, 24 jet beads and 214 blue glass beads ^[127,128]^. **Dating:** artifacts**:** 3^rd^ century. **Additional information**: This grave had not been opened; it was found intact. G-148 is a stone sarcophagus discovered at a depth of 0.30 m. The cover is made of limestone in the form of gable roof decorated with acroteria at the corners, suggesting Anatolian influence. The *acroteria* are in the shape of a quarter of a sphere. The bones are mostly dislocated, except for the long bones of the lower extremities. It is not possible to determine whether the burials were simultaneous. There were several grave goods in the sarcophagus; the pair of golden earrings with glass inserts found close to the female were made in an Eastern Mediterranean tradition; bronze coins dated in the second half of the 3^rd^ century; the bronze brooch found by the male is of similar date. Signs of metabolic disorders (enamel hypoplasia, possible scurvy, cribra femora) and mild signs of physical work on the spine were observed for the male individual.

**Ancestry summary:** A: Balkan Iron-Age related and B: Anatolia Roman-related.

**1.20.4 Rudine necropolis (12^th^ -13^th^ century)**

**Geographic Information:** The site of Rudine is located between the old and new beds of the river Mlava, about 1 km west of the former Roman city, on a narrow ridge that stretches from the mountain foothills towards the eleventh-century Byzantine fortress and episcopal town of medieval Braničevo^8^ (not to be confused with the modern town of the same name, 25 km to the east), today the village of Stari (“Old”) Kostolac about 1 km southwest of Rudine. Rudine was an undefended eastern suburb of the main medieval settlement in the area, Braničevo, which overlooked the confluence of the Mlava and Danube Rivers, and was a suffragan see of the Bulgarian archbishopric of Ohrid; it defended the overland road from the Danube frontier to Constantinople and the Holy Land, and was well connected to markets in the Middle East. Braničevo was the focal point of wars between Byzantium, which restored it after the Hungarians destroyed it in 1127-1129, and the Hungarian and the Serbian kingdoms. Serbia took control of the town in 1378/1379. It was conquered by the Turks in 1459 ^[94,129,130]^.

**Excavation History:** The first small-scale archaeological investigations were undertaken in 1978: in 1985 they extended to an area of about 1050 square meters. They distinguished two cultural horizons at the Rudine site. The first has been identified as a Roman horizon, connected to *Viminacium*. The other one is linked to the medieval period when the settlement and necropolis at Rudine were formed. The excavations uncovered 26 graves in which skeletal remains were preserved slightly worse than in other necropolises around *Viminacium*.

Sampled graves:

- **Genetic Identifier:** I32302. **Grave Identifier:** G4-1. **Grave type:** no visible burial container, W-E orientation. **Skeletal information:** adult, undetermined sex. **Grave goods:** none. **Dating**: 12^th^-13^th^ century based on stratigraphy.

**Ancestry summary:** excluded from analysis dataset based on low SNP count.

- **Genetic Identifier:** I32301. **Grave Identifier:** G-4-2. **Grave type:** no visible burial container, W-E orientation. **Skeletal information:** female, 15-20 years old. **Grave goods:** none. **Dating**: 12^th^-13^th^ century based on stratigraphy.

**Ancestry summary:** Balkans Iron Age + Anatolia Roman + Eastern Europe.

**1.20.5 Svetinja necropolis (12^th^ -13^th^ century)**

**Geographic Information:** Svetinja is located about 500 meters east of the site of Rudine, that is, the suburb of the former town of medieval Braničevo ^[131]^. It was smaller in size than Rudine and is located about 650 meters south of the Danube.

**Excavation History:** Archaeological excavations began in 1981 and continued until 1987; they distinguished three chronologically strata. The first belongs to the earlier Roman period, while the second has been dated to the 6^th^ century. The most recent horizon belongs to the 12^th^ and 13^th^ century (notably by coins of Byzantine emperors John II Komnenos and Manual I Komnenos (1118-1180) and Bulgarian imitations), and is contemporaneous with the Rudine settlement. The Svetinja and Rudine are not spatially connected, as evidenced by the lack of a contiguous cultural layer from the Middle Ages between them.

**Summary of sampled materials:** A total of 29 graves were found in the late medieval strata of the necropolis, but there were five to six other graves that had been destroyed. Individuals were mostly buried in the earth without visible containers, although a few had bricks or stones placed around the head of the deceased. Grave goods were found only in two graves (nos. 15 and 29).

Sampled graves:

- **Genetic Identifier:** I32299. **Grave Identifier:** G-4-13. **Grave type:** no visible burial container, W-E orientation. **Skeletal information:** male, approximately 30 years old. **Grave goods:** none. **Dating**: 12^th^-13^th^ century based on stratigraphy.

**Ancestry summary:** Balkans Iron Age + Anatolia Roman + Eastern Europe

- **Genetic Identifier:** I32300. **Grave Identifier:** G-4-15. **Grave type:** no visible burial container, W-E orientation. **Skeletal information:** female, 25-35 years old. **Grave goods:** A bracelet made of quadruple twisted wire. **Dating**: 12^th^-13^th^ century based on stratigraphy. **Ancestry summary:** Anatolia Roman + Eastern Europe
- **Genetic Identifier:** I32298. **Grave Identifier:** G-4-16. **Grave type:** no visible burial container, W-E orientation. **Skeletal information:** female, 8-14 years old. **Grave goods:** none. **Dating**: 12^th^-13^th^ century based on stratigraphy.

**Ancestry summary:** Balkans Iron Age.

- **Genetic Identifier:** I32297. **Grave Identifier:** G-4-17. **Grave type:** no visible burial container, W-E orientation. **Skeletal information:** male, 2-4 years old. **Grave goods:** none. **Dating**: 12^th^-13^th^ century based on stratigraphy.

**Ancestry summary:** Excluded from ancestry analysis.

**1.20.6 Više Grobalja necropolis**

The necropolis of Više Grobalja is the second largest in *Viminacium* ^[132,133]^at the southern part of the archaeological complex. Archaeological excavations are still ongoing and about 2,850 inhumation graves have been excavated so far. As with Pećine necropolis, the excavations are rescue operations due to the mining activities needed to supply the Drmno thermal power plant. Both cremation and inhumation burials occurred there from the end of the 1^st^ century CE through the 4^th^ century CE; another group of burials stemming from the Migration period is dated to the 5^th^-6^th^ centuries CE^[126,132,134]^.

Sampled graves:

- **Genetic Identifier:** I15503. **Grave Identifier:** G-105. **Grave type:** no visible burial container, NE-SW orientation. **Skeletal information:** female, 40-45 years old. **Grave goods:** 2^nd^ century pottery fragments from a bowl and a jug. **Dating**: Ceramics, stratigraphy: 2^nd^ century.

**Ancestry summary:** excluded from individual ancestry analysis based on low SNP count.

- **Genetic Identifier:** I15518. **Grave Identifier:** G-597. **Grave type:** no visible burial container, W-E orientation. **Skeletal information:** male, undetermined age. **Grave goods:** multicoloured glass gaming chip and a bronze fibula datable c. 250-600 ^[135]^. **Dating**: stratigraphy, artifacts: 3^rd^ century; radiocarbon: 211-326 cal CE (1805±20 BP, PSUAMS-9558).

**Ancestry summary:** Balkans Iron Age-related.

- **Genetic Identifier:** I15520. **Grave Identifier:** G-916. **Grave type:** no visible burial container, N-S orientation. **Skeletal information:** male, 30-40 years old. **Grave goods: a** bronze coin (illegible, possibly Domitian) and a pendant (*bulla)*. **Dating**: stratigraphy: 3^rd^ century; radiocarbon: 207-326 cal CE (1810±20 BP, PSUAMS-9559).

**Ancestry summary:** Balkans Iron Age+Central/Northern Europe+Pontic-Kazakh Steppe.

- **Genetic Identifier:** I15523. **Grave Identifier:** G-1614. **Grave type:** no visible burial container, NW-SE orientation. **Skeletal information:** male, under 50 years old. **Grave goods:** iron ring. **Dating**: stratigraphy: 3^rd^ century.

**Ancestry summary:** excluded from individual ancestry analysis based on low SNP count.

- **Genetic Identifier:** I15521. **Grave Identifier:** G-962. **Grave type:** no visible burial container, NW-SE orientation. **Skeletal information:** male, under 30 years old, only the skull remained. **Grave goods:** none. **Dating**: radiocarbon: 130-237 cal CE (1850±15 BP, PSUAMS-9560).

**Ancestry summary:** Balkans Iron Age-related.

- **Genetic Identifier:** I32305. **Grave Identifier:** G-595. **Grave type:** no visible burial container, W-E orientation. **Skeletal information:** female, over 45 years old. **Grave goods:** nine glass beads (green, blue and white, cylindrical, biconical and hexagonal), three rings of bronze wire, bronze coin issued in 251-252 CE ^[136]^. **Dating**: stratigraphy: 2^nd^ century, coin: 251-252 CE ^[136]^; radiocarbon: 25-203 cal CE (1930±20 BP, PSUAMS-11950), indicating a probable date in the second or third century CE: see next. **Additional information**: The δ15N and δ13C values indicate that her diet was largely vegetarian with significant input from C4 plants (e.g., millet), and seems not to indicate a substantial fish consumption for this individual, which reduces the possibility of a significant reservoir effect ^[105,106]^. If the association of the coin with this burial is correct, this woman died and was buried no earlier than the second half of the third century. Assuming that the tooth used for dating is indeed from the correct individual, the radiocarbon plot suggests that so late a death date is unlikely, in which case the coin is intrusive in her burial and is not a reliable indicator of the date at or after which she died and was buried.

**Ancestry summary:** Eastern European-related.

- **Genetic Identifier:** I15526. **Grave Identifier:** G-2307. **Grave type:** metal nails indicate a wooden coffin, W-E orientation. **Skeletal information:** male, adult but undetermined age. **Grave goods:** ceramic bowl (local *terra sigillata*) found to the right of the skull. **Dating**: stratigraphy: 3^rd^ century; radiocarbon date 215-326 cal CE (1800±20 BP, PSUAMS-9561). **Additional information**: the biconical bowl was decorated with relief motifs. It is a variant of the local terra sigillata. Relief space decorated with ovaries, rosettes, large leaves and animal figures. The motif of large leaves belongs to a small group of motifs that do not have a prototype in Italian and southern Gallic ornamentation. This type of leaf first appears on Reginu products in Heiligenberg, and is used by the same master in the earliest period of the Rheinzabern workshop. The bowl is dated to the 2^nd^ century and belongs to the local production. So far it is the only specimen of this type found at Viminacium. *Terra sigillata* products are extremely rare in these graves, and this one has remained intact ^[110]^.

**Ancestry summary**: Anatolia Roman-related.

- **Genetic Identifier:** I15513. **Grave Identifier:** G-343. **Grave type:** metal nails indicate a wooden coffin, N-S orientation. **Skeletal information:** male, under 40 years old. **Grave goods:** bronze coin from Antoninus Pius (after 141 CE) and pottery fragments from one gray-fired pot (type II/3) and one baked pot (type II/145). **Dating**: stratigraphy, artifacts: second half of the 2^nd^ century.

**Ancestry summary:** Anatolia Roman-related.

- **Genetic Identifier:** I15524. **Grave Identifier:** G-2012. **Grave type:** metal nails indicate a wooden coffin, W-E orientation. **Skeletal information:** male, under 50 years old. **Grave goods:** bronze coin in the mouth and pottery fragments. **Dating**: stratigraphy: 3^rd^ century.

**Ancestry summary:** Anatolia Roman-related.

- **Genetic Identifier:** I15525. **Grave Identifier:** G-2292. **Grave type:** deceased in a wooden coffin, E-W orientation. **Skeletal information:** male, undetermined age. **Grave goods:** none. **Dating**: stratigraphy: 2^nd^-3^rd^ century.

**Ancestry summary:** Balkans Iron Age + Northern Levant.

- **Genetic Identifier:** I15522. **Grave Identifier:** G-1428. **Grave type:** deceased in grave with brick construction, W-E orientation. **Skeletal information:** female, under 40 years old. **Grave goods:** Oil lamp with volutes and a rounded nozzle ^[108]^. **Dating**: stratigraphy: 3^rd^ century; brick with seal: from the 1^st^ to the 4^th^ century; lamp: from the first half of the 1^st^ to the end of the 2^nd^ century ^[108]^. **Additional information**: Bricks from the tomb construction depict a LEGVIICL seal.

**Ancestry summary:** Balkans Iron Age+Central/Northern Europe+Pontic-Kazakh Steppe.

**1.21 Výčapy-Opatovce, Nitra district (Slovakia)**

Contact persons**:** Alena Šefčáková, Juraj Bartík, Zdeněk Farkaš, Pavol Jelínek

**Geographic Information:** The Výčapy-Opatovce site is a cemetery on the right (western) bank of the Nitra river between the villages Výčapy and Opatovce in the Nitra district.

**Excavation History:** The Výčapy-Opatovce site is one of the first systematic excavations of the Early Bronze Age Nitra culture burial grounds. Between 1951 and 1954, 311 graves were examined here, although the complete burial ground could not be examined (Točík, 1979, 65). Sporadic settlement finds from the Neolithic, La Tène period and the Migration period have been reported (Točík, 1979) at Výčapy-Opatovce.

**Summary of sampled materials:** At the burial ground, radiocarbon dating of individuals from some graves (59J, 245 and 153) revealed that they date from the Early Middle Ages. Such findings are very rare in this area. Apart from the so-called Old Hungarian cemeteries (end of the 9^th^ – 10^th^ century) in the Nitra district and directly in Nitra, uncovered in 1949 during the construction of the highway close to Výčapy-Opatovce, unique grave finds of a silver earring and an iron knife datable to the Great Moravian period were documented (pers. inf. from M. Čurný). In the nearby location of Cseterdüllő, located also at Výčapy-Opatovce, two isolated graves dating according to pottery to the 8^th^–10^th^ centuries have been found at the older cremation Slavic burial ground ^[137,138]^. Together with the ones at the Nitra cemetery, they could be part of a larger Early Medieval burial ground.

We sampled one Early Medieval grave:

- **Genetic Identifier:** I13458. **Grave Identifier:** Grave 153 (Inv. No. 2438). **Grave type:** burial pit, E-W orientation. **Skeletal information:** female, 40-50 years old. **Grave goods:** none. **Dating**: radiocarbon date 772-893 cal CE (1180±15 BP, PSUAMS-10088). **Additional information**: She was buried in a crouched position on the left side. About 156 cm high ^[139]^.

**Ancestry summary**: Eastern European-related ancestry.

**1.22 Zadar, ancient *Iader***

Contact person(s): Timka Alihodžić

**Geographic Information**: Zadar is a town situated on the eastern Adriatic coast in modern-day Croatia. The necropolis in Relja town district was located about 0.5 km from the town walls along the road leading to the southeast (to the provincial capital of Salona).

**Brief settlement history**: Julius Caesar founded Zadar as a Roman colony in 48 BC (*Colonia Iulia Iader*) although the town was inhabited at least from the early Iron Age by the local population called the Liburni. *Iader* was part of the Roman province of Dalmatia. The town was situated on the peninsula and organized on Roman principles of urbanism with major streets intersected at right angles and surrounded by massive stone walls. Zadar was primarily an agricultural colony with a large seaport, baths, a public square (*forum*), an elevated *capitolium* with a temple, and a 40 km-long aqueduct bringing drinking water to the town. In 481, Dalmatia became part of the kingdom of Italy under Odoacer and later the Ostrogoths, apparently a period of decline for the town. At the beginning of the 6^th^ century it was hit by an earthquake, which destroyed entire complexes in the town ^[140–145]^.

**Excavation History**: Research history of the necropolis of *Iader* at Relja dates back to the late 19^th^ and beginning of the 20^th^ century when this necropolis was explored for the first time. Since then, a large number of graves have been discovered due to agricultural work, earth moving and several archaeological excavations. In recent times, due to construction work, rescue excavations at the site of Vrt Relja were carried by the Archaeological Museum of Zadar between 2005 and 2006, unearthing 406 graves. The final excavations at Relja were performed during 2013-2014. Together with previous excavations, the total number of recovered burials is around 2,500 (inhumations and cremations) thus making this site one of the largest Roman period cemeteries on the Adriatic ^[144,145]^.

**Summary of sampled materials**: The samples analyzed here belong to the 1998-1999 excavations at Polačišće Street (Relja district), which unearthed 46 graves, and to the 2013-2014 excavation at the site of Poliklinika (Relja district), which uncovereded 45 graves. Inhumation burials from this site are dated between the 1^st^ and 5^th^/6^th^ centuries CE ^[140–145]^.

**Sampled Graves:**

- **Genetic Identifier:** I26776. **Grave Identifier:** Poliklinika, 2013, grave 10. **Grave type:** no visible burial container, N-S orientation. **Skeletal information:** male**,** undetermined age. **Grave goods:** none. **Dating**: stratigraphy: 3^rd^-4^th^ century. **Additional information**: a large number of rocks were piled on the top of the skeleton. This grave destroyed an older cremation burial.

**Ancestry summary**: Balkans Iron Age-related.

- **Genetic Identifier:** I26775. **Grave Identifier:** Ulica Polačišće, 1998, grave 31. **Grave type:** no visible burial container, NE-SW orientation. **Skeletal information:** male, 20-30 years old. **Grave goods:** one ceramic jug was found by the legs, of a type dated between the end of the 1^st^ and the beginning of the 3^rd^ century CE. **Dating**: stratigraphy, ceramics: 2^nd^-3^rd^ centuries. **Ancestry summary:** Anatolia Roman+North Africa.

1. **Y-chromosome patterns**

We comment below on several interesting patterns about the paternal lineages found in our dataset:

-By far, the most common paternal lineage in our transect is E1b-V13, a subclade of E1b-M78 that is common in the Balkans since the Iron Age ^[146,147]^, reaching frequencies of ~30% in some present-day populations of the area ^[146,147]^. Consistent with these observations, we find this lineage mainly in males with full local Balkan Iron Age profile, but also in two males with Anatolian/Levantine ancestry and in two medieval individuals with Balkan Iron Age-related ancestry in admixed form, again showing that the arrival of Eastern European ancestry in the Early Medieval period did not drive this lineage to extinction.

-We also detect other lineages such as J2b-Z585, J2a-Z6057, R1b-PF7562 and R1b-P312-U152 which likely derive from Balkan Iron Age populations, as they were present in ancient individuals from the Balkans before the Roman period ^[19,147,148]^. The weak presence of R1b-U152 (n=2) in our Balkan transect is relevant given that this lineage was the predominant one in Iron Age individuals from the Italian Peninsula, present at frequencies above 30% ^[149–151]^.

-Five individuals belonged to R1b-Z2103. This lineage was present in the Balkans during the Bronze Age and Iron Age ^[19,147,148,152]^, and thus some of these individuals could be patrilineal descendants of Balkans Iron Age men. However, by the Late Iron Age, R1b-Z2103 was widely distributed in Eastern Europe, the Eurasian Steppe and Armenia, which means that they could also derive from new arrivals into the Balkans during the 1^st^ millennium CE. In fact, two individuals who belonged to R1b-Z2103 were clear outliers based on the autosomal data, I15551 with a Near Eastern autosomal profile and I35014 with Eastern European-related ancestry.

-Lineages T1a-L131, E1b-M123, I2a−Y16419, J1a-Z2217 were mainly associated with individuals with Anatolian genetic origin in our dataset. This is consistent with the presence of this lineages in Near Eastern males before the Roman Period ^[147,153–155]^, and with its absence or low frequency in the Balkans in this period

Lineages G2a-PF3147 and G2a-L30 were relatively common in our dataset, but their presence in Anatolia and the Balkans as well as in other European regions during the Neolithic, Bronze Age and Iron Age ^[19,147,148,156]^, hinders our ability to establish a possible proximal origin for the patrilineal ancestry in these Balkan individuals.

-Lineages with a clear Central/Northern European (I1 or R1b-U106) or Steppe (R1a-Z93) distribution ^[157–160]^ appear in the period 0-500 CE, strongly associated with autosomal ancestry derived from Central/Northern European and Pontic-Kazakh Steppe populations (see section 4 below and Figure 5 below).

-In our dataset, I2a-L621 and R1a-Z282 only appear after 700 CE (Figure 5 below; Data S2, Table 1), always associated with Eastern European related ancestry in the autosomal genome, which supports that these lineages were introduced in the Balkans by Eastern European migrants during the Early Medieval period. Lineage R1a-Z282 shows highest frequencies in Slavic-speaking groups from Central and Eastern Europe ^[160]^. Lineage I2a-L621 reaches highest frequencies in present-day populations from Bosnia-Herzegovina, Croatia and Serbia ^[161]^, and it is also present in other Slavic-speaking groups such as Russians or Ukrainians.


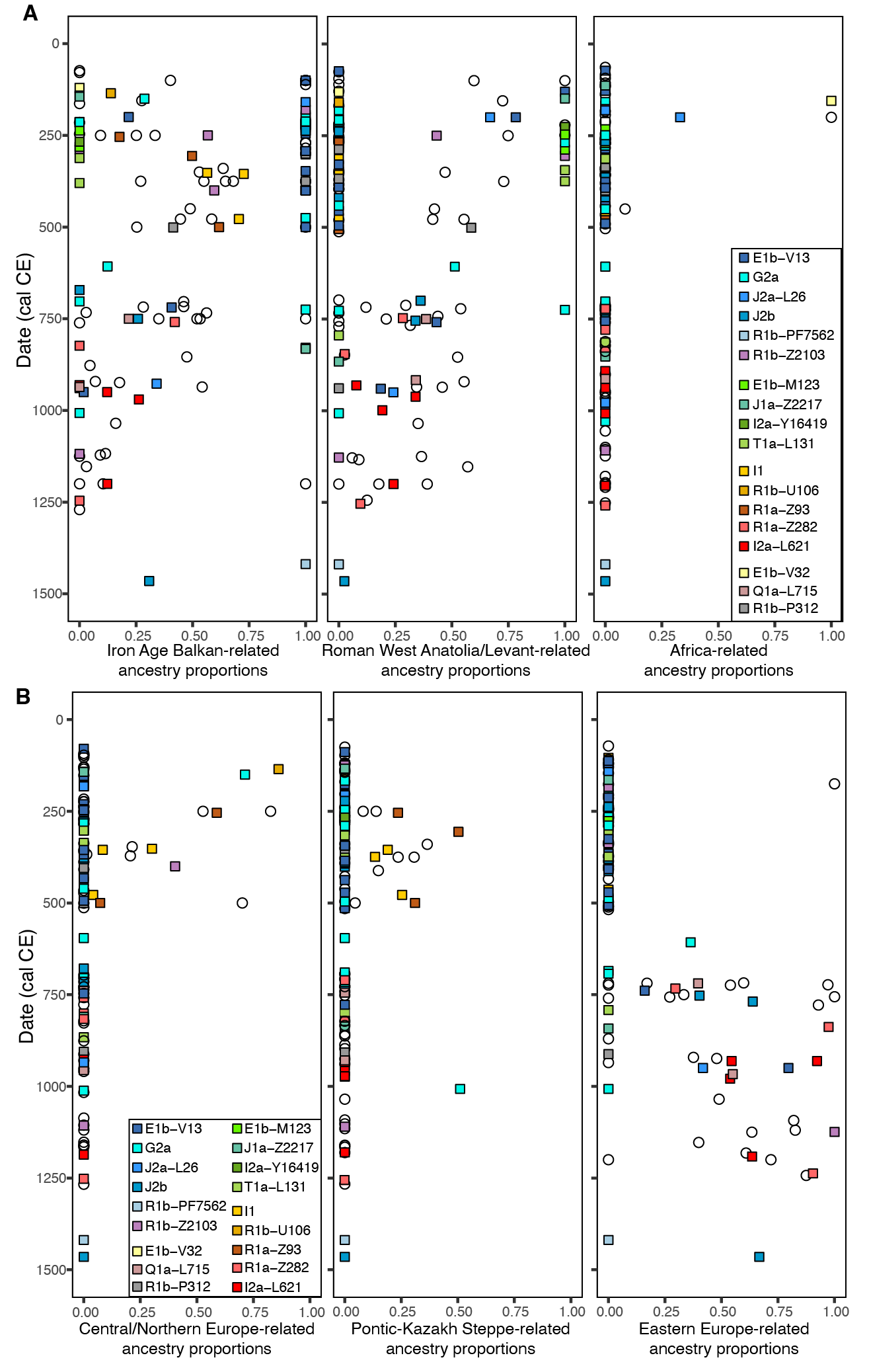


**Figure 5.** **Changes in ancestries proportions over time, correlated to Y-chromosome haplogroups**. (A) Iron Age Balkan, West Anatolia/Levant, and Africa-related ancestries. (B) Central/Northern Europe, Pontic-Kazakh Steppe and Eastern European-related ancestries.

1. **Analysis of kinship relationships within archaeological sites**

We tested for kinship relationships among pairs of newly reported individuals included in our study. For this purpose, we used the Relationship Estimation from Ancient DNA (READ) program implemented by Monroy Kuhn *et al.* ^[162]^ which can infer family relationships up to second degree even from samples with very low coverage (Data S2, Table 4). Degrees of kinship classification in a population must be independent of within-population diversity, and thus the proportion of non-matching alleles (P0) needs to be normalised before classifying relationships between pairs of individuals. This was achieved by using the expected value for a randomly chosen pair of unrelated individuals from the same population. Both window size and median pairwise P0 default options were used. We found five close kinship relationships: four first-degree pairs, one second-degree relative pair, and a pair of identical twins.

The closest kinship relationship was between I15538 (Grave 2) and I15539 (Grave 3) from Kuline Necropolis at Ravna/*Timacum Minus* (Serbia) who were found to be identical twins. In order to validate this result and rule out any possible mix-up during sampling or laboratory analysis, we took a new sample from each individual (different teeth from the initial sampling) targeting the same dental element in both individuals to minimize the possibility of sampling two teeth from the same skeleton. The new sample from Grave 3 (I26846) was again determined to be a genetic duplicate of I15538 and I15539 using READ. The new sample from Grave 2 (I26845) belonged to the same rare mtDNA haplogroup H1e1a6 (not found in any of the hundreds of available ancient individuals from the Balkans) as the other three samples, and yielded a very low number of autosomal markers in the 1240k capture. We computed the mismatch rate P0 at 1240k sites between I26845 and the merged data including the other three samples (I15538, I15539, I26846), obtaining 158 overlapping SNPs and a P0 value of 0.173 (95%CI: 0.101–0.244), a range that includes the P0 value expected for genetically identical samples but not the one expected for unrelated individuals (Data S2, Table 4). Altogether, this evidence strongly suggests that the individuals from Graves 2 and 3 are indeed identical twins.

The first-degree relationships found were between I26702 with I26708; I28390 with I34800; and I35012 with I34980.

- The I26702 and I26708 pair were found in the Dragulin cemetery at Tragurium (Croatia) and shared the same tomb (G14). The remains of both samples belonged to extremely young individuals (newborns, maximum 2 months old). I26702 was a male, while I26708 was a female, but both shared the same mitochondrial haplogroup (H-152). The archaeological information indicating similar age and the sharing of the same mitochondrial haplogroup indicates a brother and sister kinship relationship.
- I28390 (adult female; Grave 52) and I34800 (juvenile male; Grave 50-A) were found at Nuštar Dvorac necropolis (Croatia). Both shared the mtDNA lineage. The distribution of P0 values along the genome strongly indicates a parent-offspring relationship, in this case a mother-son relationship.
- Gornji Kosinj, Saint Ana I35012 (Grave 1; male) and I34980 (Grave 17; female) were found at the site of Saint Ana, Gornji Kosinj. are son-mother). Both shared the mtDNA lineage. The distribution of P0 values along the genome strongly indicates a parent-offspring relationship, very likely a mother-son relationship.

As for the second-degree relationships, we found two individuals (I15490 and I15491) from Pirivoj Necropolis (Viminacium, Serbia) buried in the same double grave G-36 who are likely second-degree relatives. Given that they have different mtDNA lineages and the same the Y-chromosome lineage, they could be paternal half-brothers, nephew and paternal uncle, or grandson and paternal grandfather.

1. ***qpAdm* admixture modelling of ancient individuals**

We modelled the ancestry of the newly reported Balkan individuals, as well as 15 previously published ^[147]^ individuals from similar historical and geographic context (Data S2, Table 1), using the *f-*statistics framework implemented in the *qpAdm* software from AdmixTools v.6. ([https://github.com/dReichLab/AdmixTools](https://github.com/DReichLab/AdmixTools)). We performed the analyses on the ‘1240k dataset’ and set the “allsnps: YES” option. When choosing populations to act as sources and outgroups in the models, we avoided including individuals with shotgun data and/or without UDG treatment whenever possible, to avoid biases that appear when different types of data are co-analyzed ^[163]^.

Given the very high ancestry heterogeneity observed in PCA, even within the same archaeological sites and time periods, we decided not to group individuals for analysis with the goal of allowing as much granular analysis as possible. The disadvantage of this strategy is that it decreases the power to reject non-fitting models, as compared to an approach where samples are grouped into populations or clusters whose ancestry is then modelled. To mitigate this problem of reduced statistical power, we excluded individuals with fewer than 40,000 SNPs for this *qpAdm* analysis and merge the data for the two individuals who were genetically identical (I15538 and I15539) (STAR methods).

We began by testing a 5-way model featuring five distal ancestries that make up the vast majority of ancestry in European and Near Eastern ancient populations ^[164]^. We used Caucasus hunter-gatherers (*CHG*), Eastern European hunter-gatherers (*EHG*), hunter-gatherers from the Iron Gates region in present-day Serbia and Romania (*Iron_Gates_HG*), Neolithic individuals from the Levant (*Levant_N*) and Neolithic individuals from Anatolia (*Anatolia_N*), as surrogates for these five distal ancestries. Individuals included under each population label are shown in Data S2, Table 3. For the outgroup set, we included:

-A group of ancient African hunter gatherer individuals from East and West Africa without evidence of Eurasian admixture as the “base” outgroup (*OldAfrica*).

-Six Neolithic and pre-Neolithic groups/individuals that provide leverage for teasing apart the five source populations because they are differentially related to them: Neolithic individuals from present-day Iran (*Iran_N*), Natufian individuals from present-day Israel (*Israel_Natufian*), the 24,000-year-old individual from Mal’ta in south-central Siberia (*MA1*), an Epipaleolithic individual from Anatolia (*Anatolia_Epipaleolithic*), Western European hunter-gatherers (*WHG*) and Mesopotamian Pre-Pottery Neolithic individuals (*Mesopotamia_PPNA*).

-One North African ancient group (*Morocco_Iberomaurusian*) and one East Asian Early Iron Age group (*Mongolia_EIA*) that will make the models fail if our test individuals harbor East Asian-related and North African-related ancestry.

Most individuals can be modelled as a mixture of the five distal source populations, but with proportions from each of the five sources varying widely across individuals (Data S2, Table 5). This confirms the observations in PCA that these individuals are genetically heterogeneous and largely descend from West Eurasian populations. A small subset of individuals, however, show a bad fit suggesting that part or all of their ancestry does not derive from any of the five West Eurasian source populations. The *qpAdm* output (when setting the “detailed: YES” option) contains information to identify the outgroup or outgroups that make the model fail. For instance, in the case of I26775, it is clear that the fitted model shares an excess of genetic drift with all the Eurasian outgroups as compared to the actual data, indicating the partial lack of non-African drift in this individual and suggesting the presence of some African ancestry that is unaccounted for by the 5-way model. In the case of I15533 or I2525, an excess of shared drift between the East Asian outgroup (*Mongolia_EIA*) and the real data as compared to the fitted model points to the presence of some East Asian ancestry in I15333 and I2525 that is not accounted for by the 5-way model.

The distal 5-way model, although useful to obtain a general view of the ancestry within the West Eurasian context of genetic variation, results in ancestry proportions derived from groups that lived more than five thousand years before our individuals of interest, and thus this model is difficult to interpret in terms of the admixture events taking place during our period of interest, roughly the 1^st^ millennium CE. We therefore tested more proximate models using source populations that are chronologically and geographically closer to our Balkan individuals from the 1^st^ millennium CE **(**Data S2, Table 6). We start with a set of outgroups containing groups distantly related (both geographically and chronologically) to our Balkan ancient individuals: *OldAfrica*, *EHG*, *Iron_Gates_HG*, *Anatolia_N*, *Iran_N*, and individuals from the Bronze Age Yamnaya, Poltavka and Afanasievo cultures (*Steppe_BA*); and more proximate groups that could provide additional leverage for teasing apart the source populations: Iron Age individuals from Iberia (*Iberia_IA*), Minoans from present-day (*Greece_Minoan*), Middle-Late Bronze Age individuals from present-day Croatia and Iron Age individuals from present-day Slovenia (*CroatiaMLBA_SloveniaIA*), Roman and Byzantine-period individuals from the Northern Levant in present-day Southeast Turkey (*SoutheastTurkey_Byzantine*), Bronze Age individuals from present-day Latvia and Lithuania (*Baltic_BA)*, Iron Age individuals from the Central and Eastern Steppe (*Steppe_IA*), and Middle Bronze Age-Iron Age individuals from present-day Netherlands (*Netherlands_MBA_IA*). Given the large number of models tested, we kept models with P-value > 0.01 to account for the multiple hypothesis testing problem.

In the West Eurasian PCA (Figure S1), Balkan populations from the Bronze Age and Iron Age appear on a cline running on PC1 with northern samples from present-day Croatia and Serbia at the low PC1 extreme of the cline, and southern samples from present-day Greece at the other with high PC1 values. To identify which of our test individuals derived entirely from local Balkan populations, we first tested models using Bronze and Iron Age groups from the Balkans as the only sources of ancestry: Iron Age individuals from Croatia (*Croatia_IA*), Bronze Age individuals from Serbia (*Serbia_BA*), Bronze Age and Iron Age individuals from Albania (*Albania_BA_IA*), Early Iron Age individuals from Bulgaria (*Bulgaria_EIA*) , and Bronze Age and Iron Age-Roman Period individuals from Greece or Greek colonies (e.g. Empúries) (*Aegean_BA_IA*). A total of 35 individuals can be modelled (P-value > 0.01) with 1-way models featuring *Croatia_IA*, *Serbia_BA, Albania_BA_IA*, *Bulgaria_EIA* or *Aegean_BA_IA*, while a two-way model featuring *Croatia_IA*+*Aegean_BA_IA* (Data S2, Table 6) works for 10 individuals. Grouping these 10 individuals to increase power and testing the same *Croatia_IA*+*Aegean_BA_IA* yields a good fit (Data S2, Table 7), confirming that the well-fitting by-individual models are not the result of a loss of statistical power to reject non-fitting models when modelling only one individual. Most of these 45 individuals date to the first half of the 1^st^ millennium CE, and we consider them as descendants of the populations inhabiting the Balkan region before the Roman Period.

Previous work has described a large-scale influx of Near Eastern ancestry in the city of Rome ^[150]^ more specifically of ancestry related to West Anatolian populations during the Roman and Byzantine periods ^[147]^. Since we observe the same shift in PCA in several individuals from *Viminacium* as well as from other sites, we tested if individuals from West Anatolia during the Roman and Byzantine periods (*WestAnatolia_Roman_Byzantine*) could be a good source of ancestry for the Balkan individuals not successfully modelled in the previous step, either as the only source or in 2-way models with “Croatia_IA” or “*Albania_BA_IA*” as the other source representing the local Balkan genetic component. The 1-way model with *WestAnatolia_Roman_Byzantine* was successful for 12 individuals (both individually and as one group; Data S2, Tables 6-7) most of whom lived during the period between 1-300 CE, while the two-way model was successful for 13 individuals (both individually and as one group) with West Anatolia-related ancestry proportions ranging from 42% to 75%, many living during the second half of the 1^st^ millennium CE. Three individuals (I15525, I15551 and I15529) who are located close to individuals from the *WestAnatolia_Roman_Byzantine* group in PCA could not be fitted using *WestAnatolia_Roman_Byzantine* as the only source or in combination with *Croatia_IA*. In the case of I15525, the *qpAdm* output indicated that the outgroup *Anatolia_N* shared significantly more drift with the fitted *Croatia_IA + WestAnatolia_Roman_Byzantine* model than with the data, suggesting that *WestAnatolia_Roman_Byzantine* harbours too much *Anatolia_N-*related ancestry to be a good source for I15525. Consequently, we removed *SoutheastTurkey_Byzantine* (a group geographically closer to Levantine populations) from the outgroup set and used it as a source instead of *WestAnatolia_Roman_Byzantine*. This improved the P-value from 7.2E-08 to 0.046 and resulted in 78% *SoutheastTurkey_Byzantine*-related ancestry (Data S2, Table 6). For the other two individuals (I15551 and I15529), the model *Croatia_IA + SoutheastTurkey_Byzantine* did not yield a good fit. Based on the PCA position of I15551, we tried 1-way models with either *SoutheastTurkey_Byzantine, SoutheastTurkey_MLBA* (Middle-Late Bronze Age individuals from Northern Levant at the site of Alalakh) or *Lebanon_Roman*, finding good fits for I15551 with both *SoutheastTurkey_MLBA* and *Lebanon_Roman* (Data S2, Table 6)*.* This suggests that this individual’s ancestry derives entirely from Northern Levantine-related populations. For the remaining individual, I15529, given his PCA position and given that he belongs to Y-lineage J1a-Z18463, which is present in ancient individuals from Southwest and Southeast Turkey, including the oldest appearance of this lineage in the paleogenomic record (I2495; 2560-2295 calBCE), the most plausible scenario is that this individual derives a large portion of ancestry from Anatolian populations. However, we caution that we were not able to obtain models with P-values > 0.01 (the best fitting model is a *WestAnatolia_Roman_Byzantine* 1-way model with P-value=0.0042).

Among the remaining 59 individual whose ancestry could not be fit with any of the previous models, three of them, I26775 from the site of Zadar, I32304 from the Pećine necropolis at *Viminacium* and I15499 from the Pirivoj necropolis at *Viminacium*, were clearly shifted to or located within African populations in PCA (Figure S2A) and as mentioned above, the *qpAdm* output suggests the presence of African ancestry that is unaccounted for by models with only Eurasian sources. Furthermore, although displaying borderline fit (P-value= 0.014) for the model *Croatia_IA + WestAnatolia_Roman_Byzantine,* individual I26709 from Dobrić necropolis at Tragurium also showed a small shift towards African populations in PCA and evidence of African ancestry not accounted by the *Croatia_IA + WestAnatolia_Roman_Byzantine* model in *qpAdm*. Based on these observations, we added to the initial outgroup set three additional groups that could provide additional leverage for discriminating African and Levantine drift: the Mota Ethiopian hunter-gatherer, Iberomaurusian individuals from present-day Morocco (*Morocco_Iberomaurusian*) and Middle-Late Bronze Age individuals from present-day Israel (*Israel_MLBA*). Adding a North African group (Copper Age individuals excavated in Sardinia and Iberia with full North African ancestry profile; *North_Africa_CA*) as a source yielded well-fitting models (Data S2, Table 6). For I26709 this was 49% *Croatia_IA + 42% WestAnatolia_Roman_Byzantine + 9% North_Africa_CA*; for I32304 this was 100% *North_Africa_CA*; and for I26775 this was *67% WestAnatolia_Roman_Byzantine+33%North_Africa_CA.* For I15499, we were able to obtain a well-fitting model without any non-African group, by combining two East African populations: Iron Age individuals from Kenya (*Kenya_IA_Pastoral*) and individuals from Northern Sudan during the Early Christian period (*Sudan_EarlyChristian*). This confirms the evidence from PCA analysis (Figure S2A) and demonstrates that individuals fully descending from African populations lived at *Viminacium* during the Early Roman Empire.

The twin individuals from the Kuline necropolis at Ravna/*Timacum Minus* could not be modelled with any of the previous models. They displayed a different ancestry makeup as compared to the other individuals from the same necropolis, with a close position to southwestern European Iron Age groups in PCA (Figure S1). A southwestern European genetic origin is also supported by their mtDNA lineage, H1e1a6, which was initially discovered in Iberian present-day populations ^[165]^ and has only been found in five other ancient individuals so far: I19987 and I19989 from Iron Age Iberia ^[148]^, I3579 from Early Medieval Iberia ^[166]^, an early 20^th^ century CE individual from Iberia ^[167]^, and MS10585 from 5^th^ century BCE Sardinia ^[168]^. Consistent with a southwestern European ancestral origin, we obtained a good fit (Data S2, Table 6) for these individuals with a 1-way model featuring Iron Age individuals from southeastern France.

At this stage, 54 individuals remained to be successfully modelled. Of these, 36 individuals were clearly shifted in the West Eurasian PCA (Figure S1) towards the present-day European cline (lower values in PC1) as compared to individuals with Balkan Iron Age-related ancestry, and shifted toward either present-day Germanic-speaking populations from Central/Northern Europe or present-day Balto-Slavic-speaking populations from Eastern Europe in the Central/Northern/Eastern European PCA (Figure S3). Finding good ancient proxies for the introduction of Central/Northern European ancestry in the Balkans that could act as sources of ancestry in *qpAdm* models is relatively easy, given the availability of previously published genomic data from ancient individuals from the relevant areas dated to the Bronze/Iron Age and Roman/Early Medieval periods, such as *Netherlands_MBA_IA* or *CNE_EarlyMedieval* (individuals from two Langobard-associated cemeteries in Hungary and Northern Italy displaying Central/Northern European-related ancestry). However, the lack of available genomic data from these periods in the Eastern European areas that are commonly identified as the Slavic homeland, such as present-day Ukraine, Belarus and Eastern Poland ^[169]^, forces us to use proxies from other areas to study the introduction of Eastern European ancestry in the Balkans. One such proxy is *CEE_EarlyMedieval,* a group of 8 Central/Eastern European Early Medieval individuals (Data S2, Tables 1 and 3) plotting within present-day Balto-Slavic-speaking populations in PCA (Figure S3), and excavated in Western Hungary (previously published), Czech Republic (previously published), Eastern Austria (newly reported in this study) and Western Slovakia (newly reported in this study). These individuals, although not excavated at the proposed Slavic homeland, lived in regions that were likely already Slavicized, displayed very similar ancestry profiles to that observed for some of the most extreme Balkan individuals in PC1 of Figure 3A in the main text and Figure S3, and belonged to Y-chromosome R1a-Z282, a lineage that we see appearing in our transect after 600 CE (Figure 5 above).

To disambiguate between Central/Northern European and Eastern European genetic affinities, we computed statistics of the form *f*_4_ (*OldAfrica, Test; Eastern European-related, Central/Northern European-related*) (Data S2, Table 12) with the ADMIXTOOLS software ^[170,171]^, with *Test* being each of the 36 Balkan individuals, *Central/Northern European-related* being a group containing *Netherlands_MBA_IA* and *CNE_EarlyMedieval*, and *Eastern European-related* being a group containing *Baltic_BA* and *CEE_EarlyMedieval.* Next, we plotted this *f*_4_-statistic against the PC1 and PC2 coordinates from the Central/Northern/Eastern European PCA in Figure S3. This figure (Figure 6 below) shows two groups of individuals, one with 23 individuals having more affinity to *Eastern European-related* groups with lower *f*_4_ values, higher PC1 and lower PC2 values, corresponding to individuals after 700 CE (with one exception, I32305). The other with more affinity to the *Central/Northern European-related* groups with higher *f*_4_ values, lower PC1 and higher PC2 values, corresponding to 13 individuals before 700 CE. Based on this separation, we grouped the 36 individuals into two clusters and tested the same statistic *f*_4_ (*OldAfrica, Test; Eastern European-related, Central/Northern European-related*) at the cluster level (Data S2, Table 12). The cluster of Balkan individuals with Eastern European shift yielded Z = -3.41, while the other cluster yielded Z = 1.99, confirming the differential affinity observed at the individual level. Furthermore, the statistic *f*_4_ (*Balkans Eastern European shift, Balkans Central/Northern European shift; Eastern European-related, Central/Northern European-related*) with Z=8.244 again shows the excess affinity between the cluster of Balkan individuals before 700 CE (n=13) with Central/Northern European populations, and between the cluster of Balkan individuals after 700 CE (n=23) with Eastern European populations.


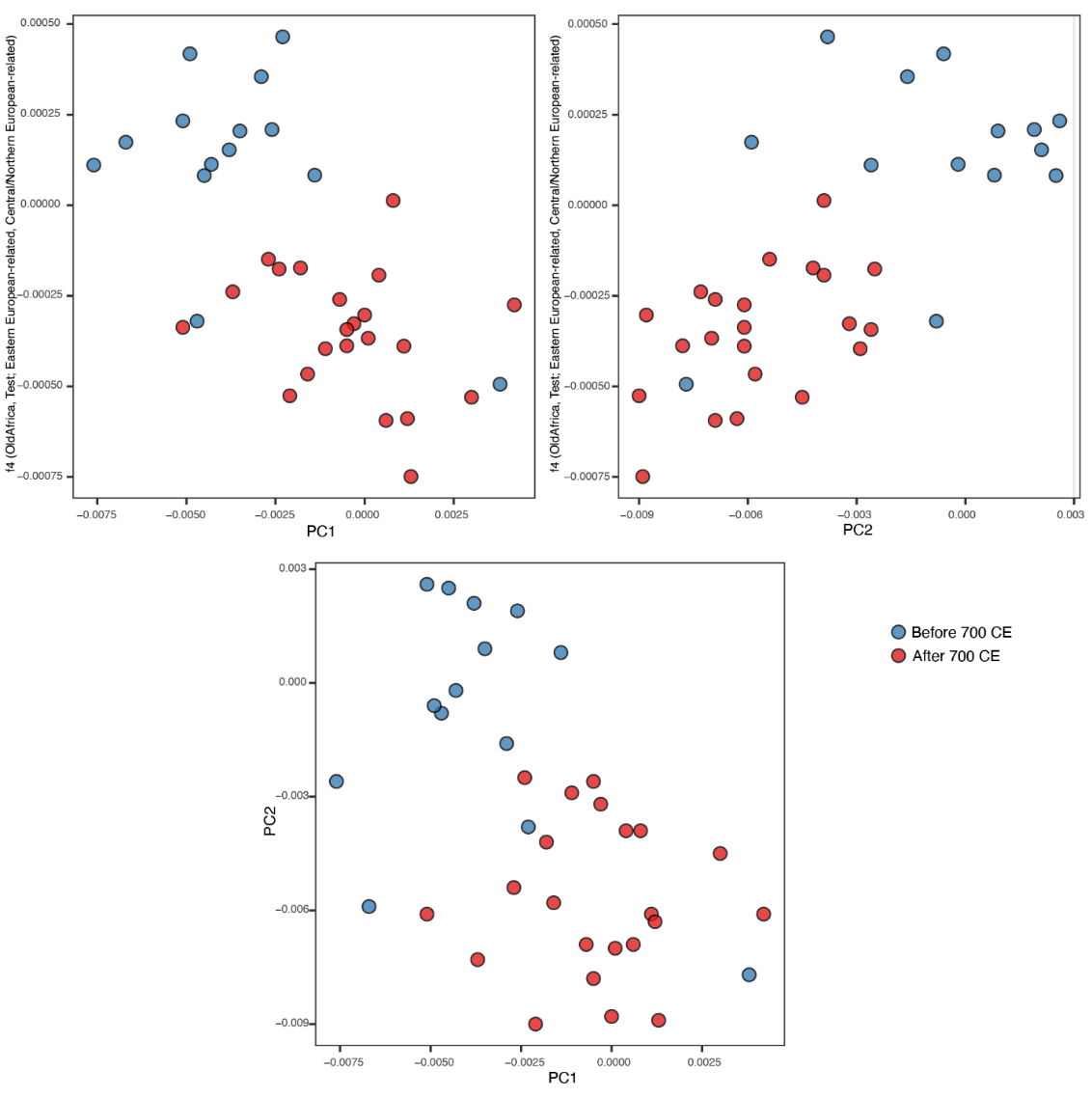


**Figure 6.** **Two-dimensional plots with PC1 and PC2 values obtained in the Central/Northern/Eastern European PCA (Figure S3), and the statistic of the form *f*_4_ (*OldAfrica, Test; Eastern European-related, Central/Northern European-related*).** *Test* represents each of the 36 ancient Balkan individuals with the strongest affinity to Central/Northern/Eastern European populations.

Next, taking advantage of the increased power to reject models, we attempted to find well-fitting *qpAdm* models at the cluster level that we could later apply separately to each individual within the cluster. For the cluster with Eastern European affinities, we found a reasonable fit (P-value=0.049) for a three-way model featuring 7% *Croatia_IA* + 16% *WestAnatolia_Roman_Byzantine+* 77% *CEE_EarlyMedieval* (Data S2, Table 7)*.* Conversely, this model displayed a very poor fit (P-value=5.04E-09) for the cluster with Central/Northern European affinities, which could instead be modelled with a three-way model featuring 42% *Croatia_IA* + 42% *CNE_EarlyMedieval +* 16% *Kazakhstan_Sarmatian_IA* (Iron Age individuals from Eastern Kazakhstan associated with the Sarmatian culture ^[172]^ that we refer to as Pontic-Kazakh Steppe-related ancestry). The later model displayed a very poor fit for the cluster with Eastern European affinities (P-value=2.70E-15). These results corroborate the distinction observed with the *f*_4_-statistics and demonstrate the presence of two different groups:

- One group (n=23) with Eastern European genetic affinities including individuals who date from the 7^th^ century CE onwards (with one exception), which is plausibly associated with the arrival of Slavic-speaking populations to Southeastern Europe and their admixture with preestablished populations.
- One group (n=13) with both Central/Northern European-related ancestry and ancestry related to Iron Age nomadic groups from the Kazakh-Pontic Steppe, themselves carrying substantial proportions of Eastern Eurasian-related ancestry. Individuals from this group mostly date to the first half of the 1^st^ millennium CE, and likely represent the known interaction between Germanic-speaking groups with Steppe populations before reaching the territories of the Roman Empire in the Balkans and admixing with local Balkan populations.

Next, we ran the model *Croatia_IA*+*WestAnatolia_Roman_Byzantine+CEE_EarlyMedieval* for each individual in the group with Eastern European affinities, and the model *Croatia_IA*+*CNE_EarlyMedieval+Kazakhstan_Sarmatian_IA* for each individual in the group with Central/Northern European affinities (Data S2, Table 6). We obtained well-fitting models for all individuals except for I32305, I15543, I35014, I34980 and I26747. In the first case, a clear outlier in PCA and dated to the Roman Period unlike the other individuals, we were able to improve the fit above 0.01 with a model featuring *CEE_EarlyMedieval+Russia_Ingria_IA* (Iron Age individuals from Ingria in Northeastern Europe), probably meaning that this individual needed more Eastern European-related ancestry than that present in *CEE_EarlyMedieval*. Similarly, for I15543 we were able to improve the fit above 0.01 with *Croatia_IA*+*WestAnatolia_Roman_Byzantine+Russia_Ingria_IA.* For I26747, I34980 and I35014, both with very high Eastern European-related ancestry, we were not able to improve fit, possibly a consequence of the lack of good proxies for the introduction of Eastern European-related ancestry.

The 18 individuals that remained to be modelled were:

-Two individuals from Sipar (I26766 and I26767) that could be modelled, both as a group and individually, with the model *Croatia_IA* + *WestAnatolia_Roman_Byzantine +* *CEE_EarlyMedieval* but with a lower proportion (33%) of *CEE_EarlyMedieval*-related ancestry as compared to the main cluster with Eastern European affinities (71%), which explains the lack of a strong shift in PCA for these two individuals*.*

-Two individuals from Pećine at *Viminacium* (I15533 and I15535) that are clearly shifted towards Steppe Iron Age populations in PCA. When we modelled them as a group, we found a good fit for 55% *Croatia_IA* + 45% *Kazakhstan_Sarmatian_IA.* This model also worked for each individual separately (Data S2, Table 6) These results again demonstrate gene-flow into the Balkans from the Steppe during the 3^rd^-4^th^ centuries CE.

-One individual from Pogorelek (I26761) that could be modelled as *Croatia_IA* + *CNE_EarlyMedieval* + *Kazakhstan_Sarmatian_IA,* like two other individuals from the same site.

-Seven individuals from Dvorac-Nuštar that could be modelled, both individually and as a group, with a local Balkan source (*Croatia_IA)* + *WestAnatolia_Roman_Byzantine +* an Eastern European-related group (*CEE_EarlyMedieval* or *Russia_Ingria_IA),* but with a substantially lower proportion of *CEE_EarlyMedieval*-related ancestry as compared to the outlier female (I28390) from the same site who had 93% of this ancestry.

-One individual from Gornji Kosinj (I35008) that could be modelled as *Croatia_IA* + *WestAnatolia_Roman_Byzantine +* *Russia_Ingria_IA,* with 53% Eastern European-related ancestry.

-Three individuals from Rudine and Svetinja (I32299, I32300 and I32301 that could be modelled with *Croatia_IA* + *WestAnatolia_Roman_Byzantine +* *CEE_EarlyMedieval* or *Russia_Ingria_IA.*

-Two Medieval individuals from Bulgaria and Romania (I2525 and I10495) with clear evidence in PCA of East Eurasian-related ancestry (Figure S2B). To successfully model these individuals, a group from the Eastern Steppe was needed as a source. We used Xiongnu individuals from Buryatia close to the Russia-Mongolia frontier (*Russia_Buryatia_Xiongnu*), but other groups from the Eastern steppe with similar ancestry profiles also provided good fits. In the case of I2525, the model 34% *WestAnatolia_Roman_Byzantine +* 55% *CEE_EarlyMedieval +* 11% *Russia_Buryatia_Xiongnu* provided a good fit the ancestry. For I10495, instead, a ~50/50 mixture between a Pontic-Kazakh Steppe group *(Kazakhstan_Sarmatian_IA)* and an Eastern Steppe group (*Russia_Buryatia_Xiongnu*) was sufficient to obtain a good fit (Data S2, Table 6), with no need of local Balkan or Near Eastern populations.

Finally, we tested the presence of sex biased admixture in the group with high Eastern European genetic affinity (n=23), by comparing ancestry proportions on the autosomes with those computed on the X-chromosome. If the incoming Eastern European related groups admixing with the local population were heavily male biased, we would expect to observe a lower proportion of Eastern European related ancestry in the X-chromosome. Previously in this section, we modelled this group as a 3-way mixture of *Croatia_IA* and *WestAnatolia_Roman_Byzantine* representing the ancestry components present in the Balkans during the first half of the 1^st^ millennium CE, and the groups *CEE_EarlyMedieval* and *Russia_Ingria_IA* representing the Eastern European-related ancestry detected in our transect starting ~700 CE. Given the small number of markers available on the X-chromosome as compared to all the autosomes, X-chromosome ancestry proportions tend to have very high standard errors, especially when the number of ancestry sources is higher than two. Thus, we tried instead a two-way model with *CEE_EarlyMedieval* or *Russia_Ingria_IA* as one of the sources, and as the other source either we merged *Croatia_IA* and *WestAnatolia_Roman_Byzantine* under the same label for this analysis, or we used the group of Roman-period Balkan individuals (*Balkans admixed*; n=13) that can be modelled as a mixture of *Croatia_IA+WestAnatolia_Roman_Byzantine* (Data S2, Table 7). We outline the results below:

Autosomes: 23.8±1.8% Local (*Croatia_IA+WestAnatolia_Roman_Byzantine*) + 76.1±1.8% *CEE_EarlyMedieval;* P-value=0.077

X-chr: 9.9±9.4% Local (*Croatia_IA+WestAnatolia_Roman_Byzantine*) + 90.1±9.4% *CEE_EarlyMedieval;* P-value=0.55

Z-score for the autosomal/X-chromosome difference: -1.45

Autosomes: 25.7±1.9% Local (*Balkans admixed*) + 74.3±1.9% *CEE_EarlyMedieval;* P-value=0.092

X-chr: 13.5±17.4% Local (*Balkans admixed*) + 86.5±17.4% *CEE_EarlyMedieval;* P-value=0.54

Z-score for the autosomal/X-chromosome difference:-0.70

Autosomes: 39.9±1.3% Local (*Croatia_IA+WestAnatolia_Roman_Byzantine*) + 60.1±1.3% *Russia_Ingria_IA;* P-value=0.051

X-chr: 36.5±14.2% Local (*Croatia_IA+WestAnatolia_Roman_Byzantine*) + 63.5±14.2% *Russia_Ingria_IA;* P-value=0.004

Z-score for the autosomal/X-chromosome difference:-0.24

Autosomes: 42.2±1.4% Local (*Balkans admixed*) + 57.8±1.4% *Russia_Ingria_IA;* P-value=0.059

X-chr: 59.1±10.5% Local (*Balkans admixed*) + 40.8±10.5% *Russia_Ingria_IA;* P-value=0.17

Z-score for the autosomal/X-chromosome difference:1.60

We do not find any clear evidence of sex biased admixture, although the large standard errors in the X-chromosome highlights the need for more data to revisit this question with higher resolution.

1. ***qpAdm* admixture modelling of present-day Balkan and Aegean populations**

With the knowledge gained through the ancestry analyses of the ancient Balkan individuals (see section 4 above), we modelled the ancestry of present-day populations from the Balkans and the Aegean, using *qpAdm*.

As documented in the main text and in previous sections, we found a significant influx of Eastern European ancestry into the Balkans from the 7^th^ century CE onwards, likely related to the arrival of Slavic-speaking populations. We therefore sought to study whether this signal persisted in present-day Balkan and Aegean populations, which would imply a long-term demographic impact in the region. The following analyses were performed using the ´HO´ dataset (Materials and Methods), after filtering out 366,668 SNPs (224,207 SNPs remained) known to produce biases when co-analyzing 1240k data (most of our ancient samples) with other types of data ^[163]^, in this case the present-day groups genotyped on Human Origins array (*64*). Unless stated otherwise, we used the same outgroups as in section 4: *OldAfrica, Steppe_BA, EHG, Iron_Gates_HG, Anatolia_N, Iran_N, Iberia_IA, Greece_Minoan, CroatiaMLBA_SloveniaIA, Netherlands_MBA_IA, Steppe_IA, Turkey_Southeast_Byzantine, Balto_BA*.

First, we attempted to model the ancestry of present-day Balkan and Aegean populations as one-way models with different Balkan Bronze Age-Iron Age populations or with later groups whose ancestry derived entirely from pre-Roman Balkan populations:

*Aegean_BA_IA*

*Croatia_IA*

*Serbia_BA*

*Albania_BA_IA*

*Bulgaria_EIA*

*CroatiaSerbia_RomanLocal*: Individuals newly reported in this study from Serbia and Croatia dated 1-500 CE and modelled as a mixture between *Croatia_IA* and *Aegean_BA_IA* in section 4 above (Data S2, Table 6-7).

If the 1-way models provided a good fit to the data, this would indicate genetic continuity in the Balkans since prior to the Roman period and no significant long-term demographic impact of the Slavic migration or other population movements in the region over the past ~2,000 years. However, all the models failed (Data S2, Table 8) with extremely low P-values (the highest p-value by far being 0.00059 for present-day Albanians modelled as *Albania_BA_IA*), strongly rejecting population continuity in the Balkans since pre-Roman times, and documenting a recent history of mixture.

Inspired by the results of the modelling of ancient individuals (section 4), we attempted to model the present-day groups with a 4-way model (Data S2, Table 8) featuring:

- A local Balkan Bronze Age-Iron Age-related source, either *Aegean_BA_IA, Albania_BA_IA, Bulgaria_EIA or CroatiaSerbia_RomanLocal*. For each present-day group, we used the geographically closest local source, inspired by the PCA which indicates some degree of local continuity.
- *CroatiaSerbia_RomanAnatolian*: Individuals newly reported in this study from Serbia and Croatia dated mostly 1-500 CE and whose ancestry derived entirely from *WestAnatolia_Roman_Byzantine* in section 4 (Data S2, Table 6-7).
- The Central/Eastern European Early Medieval individuals clustering with present-day Slavic-speaking populations *(CEE_EarlyMedieval).*
- Ottoman period individuals with Central Asian ancestry excavated in West Anatolia, Turkey (*WestAnatolia_Ottoman*) (Data S2, Table 3), to account for recent gene flow during the period of Ottoman rule in the Balkans.

In the cases where one of the sources yielded negative ancestry proportions, we removed that source and ran a 3-way model with the remaining sources. Similar to the Medieval individuals modeled in section 4, we found local Balkan Bronze Age-Iron Age-related, Anatolian Roman/Byzantine-related and Eastern European-related-ancestry in present-day Balkan populations (Data S2, Table 8). The Eastern European-related-ancestry, likely connected to the arrival of Slavic-speaking groups, decreases from north to south and it is still substantial in the Greek islands of Crete and the Cyclades, while minimal in the Dodecanese islands. We also found low proportions of Anatolia Ottoman-related ancestry, likely reflecting gene-flow over the past 500 years following Ottoman rule in the Balkans.

To ensure that the fitting models in this section were not a result of the SNP filter reducing power to reject non-fitting models, we repeated these models with a randomly down-sampled set of 224,207 SNPs. All the models yielded low p-values, indicating that ~224k SNPs are sufficient to reject models and that the applied SNP filter effectively allowed the co-analysis of 1240k and Human Origins data.
